# Supplementary material for: TRiCoLOR: tandem repeat profiling using whole-genome long-read sequencing data
Source: Gigascience. 2020 Oct 7;9(10):giaa101. doi: 10.1093/gigascience/giaa101 (PMC7539535; doi:10.1093/gigascience/giaa101)
Supplement: giaa101_GIGA-D-20-00168_Revision_1 [file giaa101_giga-d-20-00168_revision_1.pdf]

GigaScience

TRiCoLOR: tandem repeat profiling using whole-genome long-read sequencing data

--Manuscript Draft--

**Manuscript Number:**	GIGA-D-20-00168R1
**Full Title:**	TRiCoLOR: tandem repeat profiling using whole-genome long-read sequencing data
**Article Type:**	Technical Note
**Funding Information:**	GraphGenomes (031L0184C) — Dr. Jan O. Korbel<br>Associazione Italiana per la Ricerca sul Cancro (20307) — Dr. Alberto Magi
**Abstract:**	Background: Tandem repeat sequences are widespread in the human genome and their expansions cause multiple repeat-mediated disorders. Genome-wide discovery approaches are needed to fully understand their roles in health and disease but resolving tandem repeat variation accurately remains a very challenging task. While traditional mapping-based approaches using short-read data have severe limitations in the size and type of tandem repeats they can resolve, recent third-generation sequencing technologies exhibit substantially higher sequencing error rates which complicates repeat resolution.<br>Results: We developed TRiCoLOR, a freely-available tool for tandem repeat profiling using error-prone long reads from third-generation sequencing technologies. The method can identify repetitive regions in sequencing data without a prior knowledge of their motifs or locations and resolve repeats multiplicity and period size in a haplotype-specific manner. The tool includes methods to interactively visualize the identified repeats and to trace their Mendelian consistency in pedigrees.<br>Conclusions: TRiCoLOR demonstrates excellent performance and improved sensitivity and specificity compared to alternative tools on synthetic data. For real human whole-genome sequencing data, TRiCoLOR achieves high validation rates suggesting its suitability to identify tandem repeat variation in personal genomes.
**Corresponding Author:**	Davide Bolognini<br>University of Florence<br>Florence, Florence, Tuscany ITALY
**Corresponding Author Secondary Information:**	
**Corresponding Author's Institution:**	University of Florence
**Corresponding Author's Secondary Institution:**	
**First Author:**	Davide Bolognini
**First Author Secondary Information:**	
**Order of Authors:**	Davide Bolognini<br>Alberto Magi<br>Vladimir Benes<br>Jan O. Korbel<br>Tobias Rausch
**Order of Authors Secondary Information:**	
**Response to Reviewers:**	Bolognini et al., point-by-point response to reviewers<br><br>We would like to thank all the Reviewers and the Editor for their constructive input and careful assessment of our manuscript. Based on their comments, our revised TRiCoLOR method and manuscript now includes:

Powered by Editorial Manager® and ProduXion Manager® from Aries Systems Corporation

- A tandem repeat callset generated by TRiCoLOR for the Arabidopsis thaliana KBS-Mac-74 model organism
- Additional benchmarks of tools for haplotype phasing to create haplotype-tagged input files for TRiCoLOR
- Several clarifications in the main text and in the supplementary information

TRiCoLOR has also been added to SciCrunch.org (RID: SCR_018801) and to bio.tools (bio.tools ID: tricolor).

Reviewer #1

Remarks to the authors: The authors present TRiCoLOR, a tool for genotyping tandem repeats and identifying tandem repeat expansions. While long-read sequencing holds the promise to elucidate this challenging type of variant, the software ecosystem is sparse and has not fully matured. As such TRiCoLOR is a valuable development, especially given the feature of de novo detection of tandem repeats. Both the manuscript and the software documentation are well written and clear. Please find my minor comments below.

Response: We thank the reviewer for the positive assessment of our manuscript.

Comment 1: Could the authors add recommendations to either the documentation or the manuscript on how to create the phased bam input files? I see Alfred is used in the manuscript, but given that multiple tools exist (also HapCut, WhatsHap, LongShot) it would be interesting to know if a comparison has been performed to find the best tool prior to TRiCoLOR. In addition, it could be useful for users to have recommendations on how to obtain a phased SNV vcf for their data. I expect this preparation step to be rather critical to the performance of TRiCoLOR.

Response: We agree with the Reviewer that a benchmark of the available tools for long-read phasing could be interesting, as this will help readers identifying a proper strategy for preparing their data for TRiCoLOR. We have now included in our supplementary informations (Supplementary Note S1) a detailed benchmark of widely-used methods for haplotype phasing (WhatsHap, LongShot and HapCUT2). Alfred is solely used for haplotype-splitting based on phased SNVs.

Comment 2: I see the authors missed or omit tandem-genotypes (https://genomebiology.biomedcentral.com/articles/10.1186/s13059-019-1667-6), an alternative tool for sizing tandem repeats, however without haplotype-specific genotyping. Including this tool in the comparison could be informative, however, it requires the slow and cumbersome LAST alignment prior to genotyping and I am glad to see your tool is compatible with more commonly used aligners.

Response: We apologise for missing tandem-genotypes in the initial draft. We have now included tandem-genotypes in the comparison (Background section). As the Reviewer anticipated, we noticed that the required preprocessing steps (last-train and lastal) are unwieldy when it comes to generate whole-genome alignments (30 to 40 times slower than minimap2 for aligning the HGSVC ONT samples) which prevent its application at large scale.

Comment 3: The installation of the tool was without issues, but I noticed that running the executable without arguments raises an AttributeError where I would expect it to display help information. It would additionally be useful if the submodules' names could be made case-insensitive as this makes usage easier to remember.

Response: Thanks for noticing this bug. Now TRiCoLOR prints a help message when called without arguments. Following the Reviewer's suggestion, now TRiCoLOR's submodules (SENSoR, REFER, SAGE, ApP) are case-insensitive (for instance, the SENSoR module can be run as TRiCoLOR SENSoR or TRiCoLOR sensor interchangeably).

Reviewer #2

Remarks to the authors: Overall my impression is very positive. I believe the package addresses a practical use-case, and the evaluation uses reasonable experiments. However, in its current form there are several shortcomings in clarity that must be addressed. I think these can all be addressed without performing additional experiments. The following list is roughly in order of decreasing importance.

Response: We thank the reviewer for the generally positive assessment of our work. We have made several revisions based on the comments raised, as detailed below.

Comment 1: (Abstract, "The method can identify repetitive regions in sequencing data de novo ..."). I initially misinterpreted this, and it wasn't until the beginning of Methods that I figured it out. First, the use of the term de novo is easy to misunderstand, giving the reader the expectation that only sequenced reads are required. However, an assembled reference genome is required and the sequencing data has to be processed (e.g. by a haplotype-resolving aligner, not part of the package). I believe (as is mentioned at the end of Background, paragraph 4) the authors mean that the repeats themselves are discovered without any external definition of repeat motifs or locations. These distinctions should be made clearer in the abstract, and near the top of the Discussion section.

Response: We agree that the term "de novo" might create misunderstandings. We therefore now rephrased the abstract accordingly and the term is now introduced (and clarified) in the Background section (paragraph 5 and paragraph 13).

Comment 2: (Discussion "TRiCoLOR was primarily designed to profile ... microsatellites). This should be indicated in the abstract, as it will help clarify the intended use case.

Response: We have briefly reworded the Discussion section to clarify that TRiCoLOR profiles micro-satellites by default. We would like to clarify, however, that the regular expression algorithm can be tuned to profile mini-satellites as well (for instance, by setting the --size parameter to 10)

Comment 3: (Discussion, paragraph 2, "TRiCoLOR is technology agnostic and works with PB and ONT data"). Looking at the source code as well as the manuscript, the "technology agnostic" claim is a stretch. There are command line parameters that tune performance for either PB or ONT (e.g REFER readstype as mentioned in Note S7), and the evalution describes many settings to specify a technology. No evidence is presented to address anything other than PB or ONT technologies, hypothetical or real. The agnosticism claim should be justified or removed.

Response: We apologise for this inaccuracy and removed the agnosticism claim. We simply wanted to point out that TRiCoLOR works with both Oxford Nanopore Techonologies and Pacific Biosciences data.

Comment 3: (Benchmarking TRiCoLOR on real data, "...We calculated the number of TRs properly called by TRiCoLOR using a reference-free validation approach") A few sentences later it is revealed that a searchable FM-index of the human reference is used. I think the authors mean that it doesn't require a TR-annotated reference. But as it stands, the term reference-free is not how most readers would interpret it. This should be reworded to more accurately convey the concept.

Response: We thank the Reviewer for pointing this out, as it could have potentially been a source of misunderstanding for the readers. By "reference-free" we meant that the validation approach we used did not require the alignment of sequenced reads to an assembled reference genome (as this is potentially a source of biases for short-read sequencing datasets). We therefore reworded "reference-free" to "alignment-free", as this should help readers getting a clearer idea of our validation strategy.

Comment 4: (Discussion, paragraph 2, "... it can only deal with sequencing data from diploid individuals ...") It's not clear to me why this limitation exists, but a consequence of this is that it cannot identify repeats on human chromosome Y. This should be mentioned.

Powered by Editorial Manager® and ProduXion Manager® from Aries Systems Corporation

Response: We added this limitation in the Discussion section. Extending TRiCoLOR to non-diploid organisms is future work.

Comment 5: (Discussion) Because an assembled reference genome is required, the tool cannot identify repeats in genomic regions that are not assembled, such as chromosomes or telemeres. This should be mentioned with the other shortcomings.

Response: We thank the Reviewer for pointing out this oversight, we added this limitation in the Discussion section.

Comment 6: (Benchmarking TRiCoLOR on synthetic data, "... contractions/expansions of 7 motifs on average... [classification performance was] calculated allowing no discrepancies, 1 discrepancy or 2 discrepancies between the number of TRs in the ground truth and the number of TRs predicted by TRiCoLOR). It is not clear to me what a discrepancy is in this context. If the ground truth contained a TR with 10 copies of CAT, and TRiCoLOR reported a 12-copy CAT TR, is that "2 discrepancies"? My confusion may be because I think the authors are using TR and motif interchangebly here. But I have no alternative interpretation that is consistent with measuring the difference in the length of a particular TR. This description needs to be clarified.

Response: In this context we use the term "discrepancy" to indicate a different number of repeated motifs between the ground truth and TRiCoLOR's prediction. We changed "number of TRs" to "number of repeated motifs" in the text, which should help clarifying the results of our experiments.

Comment 7: (Benchmarking TRiCoLOR on real data, description of the process starting with "checks if the variant sequence appears at any position in the reference FM index") First, I think "the variant sequence is unique" is intended to mean "the variant sequence is unique in the reference". But I don't believe that is necessariy true. My interterpretation of "unique" is that the sequence occurs at one and only one position. If x is the variant sequence and it extends to, say, AxG, and AxG is not in the reference, this doesn't imply that Cx or xT is not in the reference. Nor does it imply that x occurs at only one position in the reference (though I believe the occurence count is something FM can give you).

Response: We would like to clarify a potential misunderstanding: The FM-Index is used to search all illumina reads efficiently (alignment-free). The additional comparison to the reference is only carried out to ensure that the tandem-repeat tagging sequence is long enough to not have any spurious matches in the reference. If such a tandem repeat tagging sequence does not occur in the reference but is supported by the illumina data we counted such events as true positives. This validation approach is of course bounded by the short read length, a limitation we already stated in our original submission.

Comment 8: (continuing) Second, in "up to 2 discrepancies", what is a discrepancy? Is this an edit distance of 2? In the Benchmarking on synthetic data section a discrepancy seemed to be the number of copies of the motif (I could have been wrong about that). I don't see any way to fit that meaning of discrepancy into the current context.

Response: We apologise for the misunderstanding and we indeed meant to use edit distance. We clarified this in the text and in the caption of Figure 1.

Comment 9: (Benchmarking TRiCoLOR on synthetic data, "... we compared TRiCoLOR to ... NCRF" and Figures 2 and S6). I have the same confusion here as in the previous paragraph. Figure 2 seems to make sense if the horizontal axis is the number of repeated motifs in a TR, and the vertical axis is the number of repeated motifs in the corresponding TR reported by the tool. It's difficult to evaluate this experiment as it is written. For example, I can't figure out if each of the 100 PB BAM files that contain expanded TRs contains only one TR or several.

Response: In all the simulations each BAM file harbours only one tandem repeat modification. We have highlighted this in the text and in the caption of Figure 2.

Comment 10. (Profiling repetitive regions, "... screened by a RegEx-based approximate string matching algorithm ...") I didn't find any detail about the regular expression-based approximate string matching algorithm. It's not clear whether this algorithm will find only perfectly repeated motifs or if it allows some error (a la Wu and Manber's 1992 agrep). If it only finds perfect repeats a phrase indicating that should be added to the manuscript. If it allows for errors, this should be described either in this paragraph or in a supplementary section.

Response: Thanks, we further clarified the RegEx-based approximate string matching algorithm in the Methods section.

Comment 11: (Benchmarking TRiCoLOR on synthetic data, error ratios 45:25:20 and 15:50:35). These ratios were derived from real sequencing data, as described in Note S3. A sentence or short phrase should be added, telling the reader these numbers are justified by real data and point her to S3.

Response: We have added the required informations in the text, thanks for the suggestion.

Comment 12: (Benchmarking TRiCoLOR on synthetic data, ~8000 bps). The 8K average read length is used in several simulations here, but I see no justification given. Figures S1 and S2 of Ono's 2013 paper suggest a much shorter mean length, and indeed the default for pbsim is 3K. Of course 2013 is ancient history and PB technology (probably) gives longer lengths now. I suspect the authors derived the 8K number by examining lengths in some real dataset; if so that should be mentioned in a supplementary note. (I suspect the authors may have derived it from the same data mentioned in note S3.) Moreover, one might expect ONT to given different lengths. I assume the use of 8K for both technologies is to eliminate a possible source of bias. But the authors should give some argument why the 8K value is reasonable to ONT.

Response: We derived the mean length of Oxford Nanopore Technology reads using real datasets from one of our previous works (Bolognini et al., PLoS One. 2019, Figure 2), which we have now added as a citation in the text. We indeed used the same length for ONT and PacBio to not introduce any length bias.

Comment 13: (Profiling repetitive regions, "With the haplotype-specific consensus sequences at hand ... Supplementary Note S4 ...)" Note S4 describes an evaluation of aligning raw reads to a reference, as opposed to aligning consensus sequences to reference. The conclusion that minimap2 is the winner may be true even for consensus sequences. But I'm concerned about what paramterization of minimap2 was used to map consensus to reference. I expect it should be either asm5 or asm10, and in particular the presets used for PB or ONT reads shouldn't be expected to give the best alignments for the consensuses. Consider adding a paragraph to S4, or a separate short supplemental note describing the minimap2 parameters used to align consensus to reference.

Response: We have now included in Supplementary Note S4 a benchmark of several of minimap2's presets of parameters (map-ont/map-pb, asm5, asm10 and asm20) that we used to align SPOA-generated consensus sequences to the reference genome. Although the assembly-to-reference parameters (asm5, asm10, asm20) are ideally well-suited to align consensus sequences to the reference genome, we did not notice any differences in terms of mapping accuracy between these parameters and those tuned for aligning noisy reads (map-ont/map-pb). For the time being, we are calling minimap2 from within TRiCoLOR using the map-ont/map-pb presets.

Comment 14: (Benchmarking TRiCoLOR on real data, "... the module identified ~160000, ~190000 and ~260000 low-entropy regions ...) Are these regions the same size as the SENSOR windows (i.e. 20 bp)? Or have neighboring windows been joined into longer intervals? If it is the former it would be clearer to use "window" instead of "region". If it's the latter, a statement about the average region length, or sum of regions lengths, would be appropriate. It really depends on what the author's want to convey by these numbers. If they are evidence that filtering for entropy and depth reduces the workload, the fraction of the genome excluded by these steps would be

worth knowing. If instead this is intended as a result of biological significance, the fraction of the genome covered by these regions would be interesting.

Response: Filtering on coverage depth improves the false discovery rate of TRiCoLOR and it also reduces the workload of the subsequent tandem repeat profiling step. As suggested by the reviewer, we included in the text the average length of the low-entropy regions identified by TRiCoLOR SENSoR (which resulted from merging nearby low-entropy windows).

Comment 15: (Benchmarking TRiCoLOR on real data, "...often impossible to accurately map or even assemble short reads originating from repetitive regions, thus generating errors in the available TR callsets"). "false negatives" would be clearer than "errors". Or something like "thus some TRs are missing from the available TR callsets."

Response: We thank the Reviewer of his suggestion and we changed the text accordingly.

Comment 16: (Background, paragraph 3, "... accurately deciphering TRs from long reads remains a considerable challenge due to their high error rates ...") This is ambiguous -- it's not clear (as written) whether it's long reads that have high error rates, or TRs, unless the reader has previous knowledge. Please reword it to make it clearer

Response: We reworded the text.

Comment 17: (Benchmarking TRiCoLOR on real data, "Using an Ubuntu 16.04.6 LTS desktop with Intel Xeon processors X5460, the module took ~4 hours ...") I'm used to seeing clock rates as part of the CPU specs in benchmarks like this. Is the CPU speed inherent to the processor model number?

Response: We have added the clock rate as part of our setting specification, both in the main text and in the supplementary informations.

Comment 18: (Benchmarking TRiCoLOR on real data, "we could visually confirm 42 of them in the assembly). It wasn't immediately clear to me what "assembly" means. Is the the human reference, or is it a separate assembly from HGSVC, prehaps an assembly of HG00733?

Response: We apologise for the misunderstanding. We were referring to the HGSVC de novo assembly for the HG00733 individual. We have now clarified this in the text.

Comment 19: (Note S4) PBSIM was designed to simulate PacBio error profiles. How confident should we be that just by changing the --accuracy-mean and --difference-ratio parameters that it will do a decent job of modelling ONT reads? For example, what model_qc setting is appropriate for ONT? But answering that would be beyond the scope of this paper.

Response: We independently evaluated simulated and real Oxford Nanopore Technology data sets and these settings yielded comparable insertion, deletion and mismatch error rates. Currently unknown ONT biases we may fail to model adequately but this is indeed beyond the scope of this manuscript, we believe.

Comment 20: (Note S3, "For ONT alignments, the mean error rate is ~11% and ...") It should be made clear that these are the error rates and ratios before correction. It it would also be interesting to know what the ratios are after correcton, if the authors have that information handy.

Response: We have added this information to Supplementary Note S3.

Reviewer #3

Remarks to the authors: The paper described a new method TRiCoLOR for

	discovering tandem repeat in the human genome by using long reads generated by PacBio or ONT. The authors demonstrated that it outperformed other alternative programs. The concern this reviewer has is that its application is rather limited. The application of this method could be valuable, if the authors can do the following:<br><br>Comment 1. Make it work for organisms other than human;<br><br>Response: TRiCoLOR is well suited to profile tandem repeats in any diploid organism. We agree with the Reviewer that a tandem repeat callset for an organism other than human is a valuable addition to the manuscript to convince readers of the wide applicability of our method. We therefore used TRiCoLOR for one Arabidopsis thaliana sample recently sequenced with ONT (https://www.nature.com/articles/s41467-018-03016-2). Further details of this tandem repeat callset generated by TRiCoLOR have been added in the supplementary information (Supplementary Note S8).<br><br>Comment 2. Compare its performance against assembly-alignment-based approach. Users can assemble PacBio or ONT reads into contigs, and identify tandem repeats via aligning contigs against reference genome sequences;<br><br>Response: We would like to clarify that such a comparison was part of our original manuscript, please see Table 1 in the main manuscript.<br><br>Comment 3. PacBio HIFI reads are of much improved accuracy and lengths, which may be of great value in identifying tandem repeats directly, limiting the value of TRiCoLOR.<br><br>Reponse: PacBio HIFI reads are expected to improve de novo assemblies at an albeit much higher cost per base. TRiCoLOR's RegEx-based algorithm likewise benefits directly from highly accurate reads. In addition, mapping-based approaches such as TRiCoLOR are expected to work at lower coverages in case of highly accurate reads, possibly providing cost-advantages compared to haplotype-specific assemblies that may require deeper coverage and additional data for long-range phasing information (HiC or Strand-Seq).
**Additional Information:**	
**Question**	**Response**
Are you submitting this manuscript to a special series or article collection?	No
**Experimental design and statistics**<br><br>Full details of the experimental design and statistical methods used should be given in the Methods section, as detailed in our Minimum Standards Reporting Checklist. Information essential to interpreting the data presented should be made available in the figure legends.<br><br>Have you included all the information requested in your manuscript?	Yes
**Resources**<br><br>A description of all resources used,	Yes

Powered by Editorial Manager® and ProduXion Manager® from Aries Systems Corporation

including antibodies, cell lines, animals and software tools, with enough information to allow them to be uniquely identified, should be included in the Methods section. Authors are strongly encouraged to cite Research Resource Identifiers (RRIDs) for antibodies, model organisms and tools, where possible.<br><br>Have you included the information requested as detailed in our Minimum Standards Reporting Checklist?	
**Availability of data and materials**<br><br>All datasets and code on which the conclusions of the paper rely must be either included in your submission or deposited in publicly available repositories (where available and ethically appropriate), referencing such data using a unique identifier in the references and in the "Availability of Data and Materials" section of your manuscript.<br><br>Have you have met the above requirement as detailed in our Minimum Standards Reporting Checklist?	Yes

LaTeX - Main Document (TeX file)

Click here to access/download;LaTeX - Main Document (TeX file);main.tex

Placeholder for OUP logo oup.pdf

Placeholder for journal logo gigascience-logo.pdf

GigaScience, 2020, 1–6

doi: xx.xxxx/xxxx
Manuscript in Preparation
Technical Note

TECHNICAL NOTE

TRiCoLOR: tandem repeat profiling using whole-genome long-read sequencing data

Davide Bolognini[1,3,*], Alberto Magi[2], Vladimir Benes[3], Jan O. Korbel[4] and Tobias Rausch[3,4]

[1]Department of Experimental and Clinical Medicine, University of Florence, Florence, 50134, Italy and [2]Department of Information Engineering, University of Florence, Florence, 50134, Italy and [3]European Molecular Biology Laboratory (EMBL), GeneCore, Heidelberg, 69117, Germany and [4]European Molecular Biology Laboratory (EMBL), Genome Biology Unit, Heidelberg, 69117, Germany

[*]davidebolognini7@gmail.com

Abstract

Background: Tandem repeat sequences are widespread in the human genome and their expansions cause multiple repeat-mediated disorders. Genome-wide discovery approaches are needed to fully understand their roles in health and disease but resolving tandem repeat variation accurately remains a very challenging task. While traditional mapping-based approaches using short-read data have severe limitations in the size and type of tandem repeats they can resolve, recent third-generation sequencing technologies exhibit substantially higher sequencing error rates which complicates repeat resolution.
Results: We developed TRiCoLOR, a freely-available tool for tandem repeat profiling using error-prone long reads from third-generation sequencing technologies. The method can identify repetitive regions in sequencing data without a prior knowledge of their motifs or locations and resolve repeats multiplicity and period size in a haplotype-specific manner. The tool includes methods to interactively visualize the identified repeats and to trace their Mendelian consistency in pedigrees.
Conclusions. TRiCoLOR demonstrates excellent performance and improved sensitivity and specificity compared to alternative tools on synthetic data. For real human whole-genome sequencing data, TRiCoLOR achieves high validation rates suggesting its suitability to identify tandem repeat variation in personal genomes.

Key words: long-read sequencing; tandem repeat variation; bioinformatics software

Background

Almost half of the human genome is estimated to be covered by repetitive sequences [1]. Among these, tandem repeats (TR) have been found to be involved in a range of functions such as DNA repair, chromatin organization, telomere maintenance, and regulation of gene expression [2]. Most importantly, more than 40 diseases, primarily neurological, are known to be related to TR expansions [3]. Despite their clinical importance, accurately resolving TRs remains challenging in sequencing data sets mainly because of insufficient read lengths failing to encompass entire expanded repeats or technological limitations, such as high sequencing error-rates.
Prior methods for TR profiling in short-read sequencing data sets can be broadly classified as reference-based [4, 5] or *de novo* [6, 7] approaches. While the former investigates only reads spanning known TRs, the latter can identify TRs regardless of whether their repeat motif is annotated or not in the reference. Short read methods are often inadequate to accurately resolve expanded TRs if the total repeat length is greater than the read length.
Long reads from third-generation sequencing technologies, namely Oxford Nanopore Technologies (ONT) and Pacific Biosciences (PB), have proved already invaluable for the discovery

Compiled on: August 7, 2020.
Draft manuscript prepared by the author.

of large structural variants [8] and are obvious candidates for broadening the scope of detectable TRs. However, long reads exhibit high sequencing error rates which make it difficult to accurately decipher TRs, especially in low complexity regions. Few TR detection methods for long-read sequencing data have been developed so far. Examples include PacmonSTR [9], NCRF [10], TideHunter [11], NanoSatellite [12] and Tandem-genotypes [13]. However, these tools have some limitations, either because they are technology-specific (PacmonSTR and NanoSatellite), because they are not intended to be used genome-wide (NCRF, TideHunter and NanoSatellite) or because they require substantial preprocessing steps preventing their large-scale use (Tandem-genotypes). Some tools also lack genotyping capabilities (NCRF and TideHunter) and none of the aforementioned methods is capable to profile TRs *de novo* in regions that have previously not been annotated as harboring a TR.
TRiCoLOR addresses these shortcomings of existing, alignment-based tools by allowing users to rapidly identify and genotype TRs from haplotype-resolved long-read alignments. Once low-entropy repetitive regions have been identified in sequenced long reads, TRiCoLOR exploits partial order alignment (POA) [14] to compute haplotype-specific low-error consensus sequences [15] that are further processed by means of a fast regular expression (RegEx)-based approximate string matching algorithm to resolve repeat motif and multiplicity of the discovered TRs. Detected TRs can be interactively visualized within their haplotype-specific sequence context for manual exploration of expanded or contracted repeats. For trio sequencing studies, TRiCoLOR additionally allows to trace Mendelian inheritance patterns across TR genotypes.

Methods

TRiCoLOR (Tandem Repeats Caller for LOng Reads) requires haplotype-resolved long-read alignments as input (Supplementary Note S1). It then runs a series of modules to identify and genotype TRs as described in detail below. A manual containing an in-depth explanation of how to install TRiCoLOR and run its various modules is available at https://davidebolo1993.github.io/tricolordoc, including use case examples.

Identifying repetitive regions *de novo*

TRiCoLOR can identify repetitive regions in haplotype-resolved BAM files *de novo*. This is achieved using the SENSoR (Shannon ENtropy ScanneR) module, which uses the Shannon entropy of DNA sequences to identify candidate repetitive segments in genomic sequences [16]. TRiCoLOR SENSoR scans in parallel the haplotype-specific BAM files and computes, for each sequencing read, its Shannon entropy content in non-overlapping, sliding windows of a pre-trained size (20 bps, by default). Genomic coordinates of windows in which multiple reads ($\geq$5, by default) support an entropy drop ($\leq$ 1.23, by default) are stored and those nearby are merged (those falling within 100 bps intervals, by default). The default entropy treshold of 1.23 efficiently discriminates between repetitive and non-repetitive DNA sequences using synthetic ONT and PB reads as shown in Supplementary Figure S1 (see Supplementary Note S2). All candidate repetitive regions identified with this approach are eventually outputted in BED format. This pre-filtering of repetitive regions is fairly fast even in deep-coverage whole-genome data (see also *Findings*) and drastically reduces the computational time required for the subsequent TR profiling.

Profiling repetitive regions

TRiCoLOR can profile TRs in haplotype-resolved BAM files through the REFER (REpeats FindER) module. The input of REFER is a BED file generated by TRiCoLOR SENSoR. Alternatively, the BED file can be provided by the user based on prior knowledge of clinically relevant TRs, for instance.
For each region in the BED file, REFER first fetches from the haplotype-specific BAM files the sequencing reads spanning the selected region and trims them, so that the length of each read is approximately the size of the region. Let $R = [S, E]$ be a region from the BED file, ranging from a start coordinate S to an end coordinate E for a given chromosome. Each sequencing read entirely spanning R is fetched and trimmed so that the actual sequence REFER stores is that included between S and E, which significantly improves the runtime of the subsequent POA algorithm to generate a consensus sequence.
Once the sequencing reads of interest have been collected and trimmed, TRiCoLOR uses SPOA [17], a single-instruction multiple-data accelerated version of the robust POA framework, to compute highly accurate consensus sequences with an approximate error reduction of ~77% and ~88% for ONT and PB, respectively (Supplementary Note S3 and Supplementary Figure S2).
With the haplotype-specific consensus sequences at hand, REFER aligns these to the reference genome using minimap2 [18], which compared favorably to alternative aligners on syntethic data, both in terms of speed and mapping accuracy (Supplementary Note S4 and Supplementary Figure S3). The reference-aligned low-error consensus sequences are then screened by a RegEx-based approximate string matching algorithm, which has three processing steps: (1) identifying motifs (motifs of length $\leq$ 6, by default) that are perfectly repeated a minimum number of times (5, by default); (2) looking for approximate repetitions of the identified motifs to account for remaining consensus errors, that is imperfect repeated motifs up to a user-defined edit distance ($\leq$ 1, by default); (3) in case of multiple overlapping approximate repetitions, resolving these competing tandem repeat predictions using an N-gram model that favors the most frequently occurring perfect repeat motif.
Together with the haplotype-specific consensus sequences, the corresponding reference is screened in a similar manner, with few differences being noteworthies: (1) the algorithm assumes the reference does not contain errors and does not look for approximate repetitions of the motifs identified; (2) among overlapping repetitions, the longest repeat is taken.
TRs (those $\geq$ 50 bps, by default) varying between the haplotypes or the reference are eventually stored in BCF-compliant format. TRiCoLOR REFER also stores in the output folder several BED files describing the TRs identified (both for the reference and each haplotype) and haplotype-specific BAM files containing the aligned consensus sequences.

Visualizing identified repeats

The TRs profiled using TRiCoLOR REFER can be interactively visualized through the ApP (Alignment Plotter) module. This module takes as inputs the BED and the BAM files generated by TRiCoLOR REFER together with an additional BED file describing one or more regions to plot.
TRiCoLOR ApP produces a static HTML file illustrating the alignment between the reference and the individual's haplotypes at single base resolution, highlighting the TRs detected. (Supplementary Note S5 and Supplementary Figures S4A-S4C).

Tracing Mendelian inheritance patterns of identified repeats

In pedigree studies, assigned genotypes can be either Mendelian consistent or inconsistent. TRiCoLOR enables genotype consistency checks for TRs identified in the index child when haplotype-resolved long-read alignments for both parents are available. This is achieved through the SAGE (SAmple GEnotyper) module with special emphasis on the common situation that parents have been sequenced at low depth.
Using the same aforementioned TRiCoLOR REFER approach, SAGE computes haplotype-specific consensus alignments for each child TR in each parent. Next the module checks whether the parental TRs are more similar (*i.e.*, have a lower edit distance) to the reference or to the TR identified in the child and assigns them the most likely genotype. Knowing the genotype of both parents, the module eventually flags each TR as Mendelian consistent or inconsistent with the −*mendel* parameter enabled. The output of TRiCoLOR SAGE is a multi-sample BCF file that contains the genotypes for the index child and both parents.

Findings

We benchmarked TRiCoLOR using both synthetic data generated with VISOR [19] and real, publicly available data from the Human Genome Structural Variation Consortium (HGSVC) [8].

Benchmarking TRiCoLOR on synthetic data

We used the TR simulator VISOR to generate synthetic ONT and PB alignments containing TR contractions and expansions. First, we simulated haplotype-resolved ONT and PB BAM files (the average length of simulated reads was set to 8000 bps based on statistics derived from recent ONT sequencing runs [20]; the substitution:insertion:deletion ratio was set to ~45:25:30 for the synthetic ONT reads and to ~15:50:35 for the synthetic PB reads, in accordance with findings described in Supplementary Note S3) exhibiting variable error rates (accuracy of reads ~0.85, ~0.90 and ~0.95) and depth of coverage (haplotype-specific depth of coverage 5X-10X and 10X-20X), with each BAM file harboring a heterozygous contraction or expansion of a known, randomly chosen, TR. At this stage, we simulated small TR contractions/expansions (contractions/expansions of 7 motifs on average) in order to evaluate the capability of our method to spot even minor changes in the TR multiplicity of the 2 haplotypes. For each group, we simulated 200 haplotype-resolved BAM files. Then, we evaluated the performances of TRiCoLOR in terms of precision (P), recall (R), and F1 score (F1) (Supplementary Note S6). In particular, P, R and F1 values were calculated allowing no discrepancies, 1 discrepancy or 2 discrepancies between the number of repeated motifs in the ground truth and the number of repeated motifs predicted by TRiCoLOR. Figure 1 shows these findings for synthetic TR contractions (panel A) and expansions (panel B). TRiCoLOR demonstrated high P and R in all the simulated groups: our method always achieved an F1 close to 1 when allowing a single-motif discrepancy between simulated and predicted TRs and hit P ~1 and R ~1 when allowing up to 2 motif discrepancies. For both contractions and expansions the F1 depends on the coverage and input read accuracy as expected. In all the simulated TR contractions and expansions, TRiCoLOR was also able to properly identify the correct repeated motif, few times shifted (*e.g.*, a repeated TG instead of a repeated GT). Supplementary Figure S5 illustrates these findings for the same simulated groups of Figure 1, averaged over the different accuracy levels.
Furthermore, as a proof of concept, we compared TRiCoLOR to a TR caller for long reads recently published, namely NCRF. Using the same approach described above, we simulated 100 ONT and 100 PB BAM files (accuracy of reads ~0.90, depth of coverage for each haplotype 5X-10X), each harboring a small TR contraction/expansion and we run both TRiCoLOR and NCRF on these data. As NCRF cannot deal with BAM input, we slightly modified TRiCoLOR to store in FASTA format the sequences used for the consensus computation step, which could be processed through NCRF (Supplementary Note S7). Figure 2 shows the correlation results between the number of repeated motifs in the ground truth and the number of repeated motifs predicted by TRiCoLOR and NCRF for the simulated TR contractions (panel A) and expansions (panel B). For both TR contractions and expansions, TRiCoLOR got excellent R scores (R = 0.97 for contractions and R = 0.86 for expansions), outperforming NCRF (R = 0.87 for contractions and R = 0.74 for expansions). We next evaluated exceptionally long TR expansions because these have been implicated in several neurological disorders. For instance, the common Fragile-X Syndrome is related to a CGG-repeat usually consisting of $\leq$55 repeated motifs that expands to $\geq$ 200 repeated motifs. Following the simulation schema described above, we generated 100 ONT and 100 PB synthetic BAM files harboring TRs expanded by 200 motifs and we run both TRiCoLOR and NCRF on these data. Supplementary Figure S6 shows the correlation results between the number of repeated motifs in the ground truth and the number of repeated motifs predicted by TRiCoLOR and NCRF for the simulated long TR expansions. As above, TRiCoLOR achieved the best R score (R = 0.73), outperforming NCRF (R = 0.53).

Benchmarking TRiCoLOR on real data

We applied TRiCoLOR to call TRs *de novo* on publicly available ONT and PB human whole-genome sequencing data from the HGSVC project. In particular, we used the ONT sequencing data for HG00514 (Han Chinese), HG00733 (Puerto Rican) and NA19240 (Yoruban Nigerian) and the PB sequencing data for HG00731 (Puerto Rican, father), HG00732 (Puerto Rican, mother) and HG00733 (son).
We aligned the ONT FASTQ files to the human GRCh38 reference genome using minimap2 and we merged the chromosome-specific PB alignments using samtools [21]. We then split the ONT and PB alignments by haplotype with Alfred [22] using phased single-nucleotide variants from the HGSVC project. We calculated the coverage of the initial and the haplotype-resolved BAM files using mosdepth [23]. For all the ONT samples, we identified an initial ~20X coverage (HG00733 ~21X, HG00514 ~23X and NA19240 ~24X), slightly reduced after splitting by haplotype due to some unassigned reads (HG00733 ~8X, HG00514 ~9X and NA19240 ~10X for each haplotype). For the PB samples, we identified a ~42X coverage for HG00733 and ~21X coverage for HG00731 and HG00732, reduced after splitting the data by haplotype (HG00733 ~14X, HG00731 and HG00732 ~8X for each haplotype).
We then run TRiCoLOR SENSoR using the default parameter settings on the HG00733 (ONT and PB), HG00514 and NA19240 individuals. Using an Ubuntu 16.04.6 LTS desktop with Intel®Xeon®processors X5460 (clock rate 2.93 GHz), the module took ~4 hours to scan the ONT samples and ~8 hours to scan the PB sample, which reflects the higher coverage available for PB. For the HG00733, HG00514 and NA19240 ONT individuals the module identified ~160000, ~190000 and ~260000 low-entropy regions (average length of the regions ~900 bps), which were reduced to ~70000, ~100000 and ~160000 respectively after filtering for regions with average coverage > 8.

For the HG00733 PB individual the module identified ~380000 low-entropy regions (average length of the regions ~850 bps), which were reduced to ~150000, after filtering for regions with average coverage > 10. For HG00733, ~97% of the low-entropy regions originally identified in the ONT individual overlapped those in the PB one; due to the different coverage distributions, this percentage was reduced to ~31% after filtering.

We run TRiCoLOR REFER on the samples processed by TRiCoLOR SENSoR using the default parameter settings. With 7 processors on our Ubuntu desktop, the module took ~10-12 hours to profile TRs on the ONT individuals and ~14 hours to profile TRs on the PB individual.

We calculated the number of TRs properly called by TRiCoLOR using an alignment-free validation approach. Current benchmarks for TR calling in human genomes are mainly based on short-read sequencing and are biased towards regions of the genome that are easy-to-call with such a technology [24]. It has been shown that it is often impossible to accurately map or even assemble short reads originating from repetitive regions [25], and as a consequence of this, some TRs are missing from the available TR callsets. Following the idea from Dolle *et al.* [26] we first built full-text searchable FM indexes [27] both for the GRCh38 human reference FASTA and the high-quality Illumina FASTQ files of the HG00733, HG00514 and NA19240 individuals. Then, for each individuals' variant identified by TRiCoLOR REFER, the validation algorithm: (1) checks if the variant sequence appears one or more times in the reference FM index: if so, using the consensus BAM files stored by TRiCoLOR REFER, the variant sequence is extended by 1 bp to the left and 1 bp to the right and step 1 is repeated; if not, the algorithm proceeds to the next step; (2) checks if the variant appears one or more times in the corresponding Illumina FM index: if so, the variant is considered a valid call; if not, the variant is considered an invalid call. Taking into account possible errors both in the consensus sequences generated by TRiCoLOR and in the Illumina sequences, we counted as valid calls also variants that are found in the Illumina FM indexes with up to 2 bp discrepancies (*i.e.* their edit distance is ≤ 2). Limited by the length of the available Illumina sequences, using this approach we could not validate variant TRs longer than 124 bps. Overall, we got high validation ratios (ratios between the valid calls and the number of calls that could be assessed using short reads): ~82% for HG00733 (ONT and PB), ~85% for HG00514 and ~86% for NA19240 (Supplementary Figure S7).

We eventually run TRiCoLOR SAGE on the Puerto Rican PB trio HG00731, HG00732 and HG00733, with the default parameter settings and the *–mendel* parameter enabled to check the Mendelian consistency of the TRs identified in HG00733. With 7 processors on our Ubuntu desktop, the module took ~2 hours to complete the analysis. Filtering for variants differing from the reference for at least 10 bps and for multi-allelic variants differing from each other by the same distance, we identified ~80% of Mendelian consistent TRs, which is low compared to trio-based single-nucleotide variant and InDel Mendelian consistency rates, but above reported genotype agreement rates for structural variants in repetitive regions [28].

Among the Mendelian consistent TRs called by TRiCoLOR on the HG00733 PB individual, we identified 32 long TRs (≥ 150 bps) that were absent in the HGSVC ground truth for the same individual. In order to identify the cause of these apparent discrepancies, we aligned the HG00733 phased contigs from HGSVC to the GRCh38 human reference genome with minimap2, using the assembly-to-reference alignment mode and the parameters suggested by QUAST-LG [29] and we manually inspected the discordant TRs in the aligned contigs using IGV [30]. As shown in Table 1, out of 58 non-reference TR alleles identified by TRiCoLOR, we could visually confirm 42 (~75%) of them in the HGSVC assembly, which means that both TRiCoLOR and the HGSVC predicted the same variant type (deletion or insertion) and the predicted variant size is roughly similar (*i.e.* the difference does not exceed 50 bps). However, for the other 16 variants (~25%), the HGSVC assembly either did not contain the allele predicted by TRiCoLOR or did not cover the investigated region, which suggests that mapping-based and assembly-based approaches can be complementary for TR detection using long reads.

Table 1. Comparison between TRiCoLOR's mapping-based and HGSVC's assembly-based approaches for Mendelian consistent long TRs identified by TRiCoLOR on the HG0733 PB individual.

chromosome	start	end	HGSVC assembly*	TRiCoLOR call*
chr1	23703657	23703893	DEL;INS	DEL;INS
chr1	223672571	223672681	INS;INS	INS;INS
chr10	69539376	69539572	INS;INS	INS;INS
chr11	79190887	79191145	**REF**;**REF**	**DEL**;**INS**
chr11	128436913	128437081	INS;INS	INS;INS
chr14	84276747	84276903	**REF**;DEL	**INS**;DEL
chr15	70364402	70364587	INS;**NA**	INS;**INS**
chr16	3529535	3529854	REF;**DEL**	LC;**INS**
chr17	27525992	27526118	INS;INS	INS;INS
chr18	44544809	44545037	INS;INS	INS;INS
chr18	59081301	59081379	INS;**INS**	INS;**INS**
chr18	71198388	71198450	REF;**NA**	REF;**INS**
chr2	160426201	160426342	INS;INS	INS;INS
chr2	211860947	211861156	DEL;**NA**	DEL;**INS**
chr21	35063465	35063588	INS;INS	INS;INS
chr22	46174187	46174274	REF;INS	REF;INS
chr3	13856835	13857013	DEL;INS	DEL;INS
chr4	13807826	13807982	REF;**REF**	REF;**INS**
chr4	18837113	18837320	INS;DEL	INS;DEL
chr4	81637241	81637408	DEL;DEL	DEL;DEL
chr5	54513584	54513735	**REF**;INS	**INS**;INS
chr6	25450910	25450975	REF;**INS**	REF;**INS**
chr6	55543085	55543393	INS;INS	INS;INS
chr6	106945844	106946002	DEL;**DEL**	DEL;**INS**
chr7	38610247	38610412	**NA**;DEL	**INS**;DEL
chr7	71847696	71847865	INS;INS	INS;INS
chr7	109663557	109663744	INS;DEL	INS;DEL
chr7	131933466	131933651	INS;INS	INS;INS
chr9	82850174	82850347	DEL;DEL	DEL;DEL
chr9	91622218	91622365	**NA**;NA	**INS**;REF
chr9	91634814	91634973	**NA**;**NA**	**DEL**;**INS**
chr9	116632126	116632280	INS;INS	INS;INS

* DEL indicates a deletion; INS indicates an insertion; REF indicates a reference allele; NA indicates that the region is not covered by the assembly or mis-assembled; LC indicates that TRiCoLOR could not generate a consensus sequence for the allele due to the low coverage in the region. The 2 alleles are separated by a semicolon.

Discussion

TRiCoLOR is a comprehensive TR caller for long reads that supports the *de novo* identification of TRs in whole-genome sequencing data. TRiCoLOR profiles TRs through an efficient POA algorithm combined with a RegEx-based string matching search, facilitating a robust and accurate discovery of the full spectrum of expanded and contracted TRs in personal genomes.

In comparison to previous tools, TRiCoLOR works with ONT and PB data seamlessly. TRiCoLOR also identifies TRs *de novo* and does not require *a priori* knowledge of annotated TR regions. The unique combination of features for genome-wide, *de novo* discovery and genotyping of TRs in ONT and PB data is to the best of our knowledge unmet by any other TR caller for long-read data. Besides the detection of TRs, TRiCoLOR visualizes TRs in their haplotype context and it can infer parental genotypes using low-coverage parental sequencing data.

TRiCoLOR has been designed for diploid organisms (Supplementary Note S8) and future work includes extending its fea-

ture set to polyploid species and haploid chromosomes (human Y chromosome). As a mapping-based approach, TRiCoLOR cannot identify repeats in unassembled regions of the genome (*e.g.*, human centromeres and telomeres). Furthermore, the entropy threshold and window size for the *de novo* identification of repetitive stretches that we empirically estimated is well-suited for short repeated motifs (2-3 bps) but may need adjustments for long motifs of higher nucleotide complexity. Lastly, by default TRiCoLOR profiles TRs with motif lengths $\leq$ 6 bps (also known as micro-satellites), excluding those with motif lengths $\geq$7 bps (also known as mini-satellites), which are less abundant in diploid organisms [31]. The RegEx algorithm can be also tuned to profile mini-satellites (*i.e.*, by extending the *–size* parameter) but TRiCoLOR has been extensively applied so far only to micro-satellites.
Given these limitations, future work will focus on extending TRiCoLOR to other ploidies, broadening the size spectrum of detectable repeat motif lengths and taking advantage of improved sequencing read accuracy (*e.g.*, high-fidelity long reads from PB). The latter directly improves the RegEx-based identification of repeats employed by TRiCoLOR and we thus believe TRiCoLOR is well-suited to characterize the TR landscape in present and future long-read data sets, making it an instrumental tool to robustly decipher the multiplicity of TRs in repeat-mediated clinical disorders.

Availability of source code and requirements

- Project name: TRiCoLOR (SciCrunch RRID: SCR_018801; bio.tools ID: tricolor)
- Project home page: https://github.com/davidebolo1993/TRiCoLOR. A dockerized version of TRiCoLOR is available at https://hub.docker.com/r/davidebolo1993/tricolor. Online documentation is available at https://davidebolo1993.github.io/tricolordoc.
- Operating system: Unix
- Programming languages: Python, Bash, C++
- Other requirements: Python 3.6 or higher, GCC 4.8 or higher and CMake 3.2 or higher.
- License: GNU Lesser General Public License 3.0

Availability of supporting data and materials

HGSVC whole-genome long-read sequencing data are available on the HGSVC website (https://www.internationalgenome.org/human-genome-structural-variation-consortium). Specifically:

- ONT FASTQ files: http://ftp.ebi.ac.uk/1000g/ftp/data_collections/hgsv_sv_discovery/working/20181210_ONT_rebasecalled
- PB alignments: http://ftp.ebi.ac.uk/1000g/ftp/data_collections/hgsv_sv_discovery/working/20180102_pacbio_blasr_reheader
- Phased single-nucleotide variants: http://ftp.ebi.ac.uk/1000g/ftp/data_collections/hgsv_sv_discovery/working/20170323_Strand-seq_phased_FB%2BGATK_VCFs
- Illumina FASTQ files: http://ftp.ebi.ac.uk/1000g/ftp/data_collections/hgsv_sv_discovery/illumina_wgs.sequence.index
- HG00733 phased contigs: http://ftp.ebi.ac.uk/1000g/ftp/data_collections/hgsv_sv_discovery/working/20180227_PhasedSVGenomes
- HG00733 ground truth of structural variant calls: http://ftp.ebi.ac.uk/1000g/ftp/data_collections/hgsv_sv_discovery/working/20180627_PanTechnologyIntegrationSet/HG00733.merged_nonredundant.vcf

The GRCh38 human reference genome used for alignments is available at http://ftp.ebi.ac.uk/1000g/ftp/technical/reference/GRCh38_reference_genome/GRCh38_full_analysis_set_plus_decoy_hla.fa. The corresponding annotated TRs can be accessed through the UCSC Table Browser tool (http://genome.ucsc.edu).
A whole-genome ONT FASTQ file of the *Arabisopsis thaliana* KBS-Mac-74 is available at ftp://ftp.sra.ebi.ac.uk/vol1/fastq/ERR217/003/ERR2173373/ERR2173373.fastq.gz. The TAIR10 reference genome for *Arabidopsis thaliana* can be downloaded through the Arabidopsis Information Resource database (https://www.arabidopsis.org/index.jsp) Several scripts used to perform the analyses described in this paper and the TR calls generated by TRiCoLOR for the HGSVC individuals and the *Arabisopsis thaliana* KBS-Mac-74 are available through the GitHub code repository of TRiCoLOR (https://github.com/davidebolo1993/TRiCoLOR). More in detail:

- the https://github.com/davidebolo1993/TRiCoLOR/tree/master/paper/data folder contains the BED file with annotated TRs from the GRCh38 human reference genome (`GRCh38.TRs.bed`), a bash script that illustrates how to haplotype-resolve a long-read alignment using phased single-nucleotide variants (`prepare.sh`), a python script used for the Shannon entropy simulations (`entropy.py`), a python script used to calculate precision, recall and F1 scores of TRiCoLOR on synthetic data (`pr.py`) and a couple of C++ source code files (`fmindex.cpp` and `validate.cpp`) for validating TRiCoLOR calls on real human data.
- the https://github.com/davidebolo1993/TRiCoLOR/tree/master/paper/samples folder contains TRiCoLOR calls for the HGSVC individuals and the *Arabisopsis thaliana* KBS-Mac-74 in standard BCF format.

Declarations

List of abbreviations

TR: tandem repeat; ONT: Oxford Nanopore Technologies; PB: Pacific Biosciences; POA: partial order alignment; RegEx: regular expression; HGSVC: Human Genome Structural Variation Consortium; P: precision; R: recall; F1: F1 score.

Competing Interests

The authors declare that they have no competing interests.

Funding

JOK is supported by GraphGenomes grant 031L0184C. AM is supported by AIRC grant 20307. The funders had no role in study design, data collection and analysis, decision to publish, or preparation of the manuscript.

Author's Contributions

DB and TR designed and benchmarked the software. DB wrote the code. TR supervised the work. DB and TR co-wrote the manuscript draft. AM, VB and JOK contributed to the interpretation of the results, provided critical feedback and helped to write the manuscript. All the authors read and approved the manuscript.

Acknowledgements

The authors thank HGSVC for data access, and EMBL GeneCore and IT for technical support.

References

1. de Koning APJ, Gu W, Castoe TA, Batzer MA, Pollock DD. Repetitive elements may comprise over Two-Thirds of the human genome. PLoS Genetics 2011;.
2. Hannan AJ, Tandem repeats mediating genetic plasticity in health and disease; 2018.
3. Paulson H. Repeat expansion diseases. In: Handbook of Clinical Neurology; 2018.
4. Gymrek M, Golan D, Rosset S, Erlich Y. lobSTR: A short tandem repeat profiler for personal genomes. Genome Research 2012;.
5. Highnam G, Franck C, Martin A, Stephens C, Puthige A, Mittelman D. Accurate human microsatellite genotypes from high-throughput resequencing data using informed error profiles. Nucleic Acids Research 2013;.
6. Doi K, Monjo T, Hoang PH, Yoshimura J, Yurino H, Mitsui J, et al. Rapid detection of expanded short tandem repeats in personal genomics using hybrid sequencing. Bioinformatics 2014;.
7. Velasco A, James BT, Wells VD, Girgis HZ, Elofsson A. Look4TRs: A de novo tool for detecting simple tandem repeats using self-supervised hidden Markov models. Bioinformatics 2020;.
8. Chaisson MJP, Sanders AD, Zhao X, Malhotra A, Porubsky D, Rausch T, et al. Multi-platform discovery of haplotype-resolved structural variation in human genomes. Nature Communications 2019;.
9. Ummat A, Bashir A. Resolving complex tandem repeats with long reads. Bioinformatics 2014;.
10. Harris RS, Cechova M, Makova KD, Birol I. Noise-cancelling repeat finder: Uncovering tandem repeats in error-prone long-read sequencing data. Bioinformatics 2019;.
11. Gao Y, Liu B, Wang Y, Xing Y. TideHunter: Efficient and sensitive tandem repeat detection from noisy long-reads using seed-and-chain. In: Bioinformatics; 2019. .
12. De Roeck A, De Coster W, Bossaerts L, Cacace R, De Pooter T, Van Dongen J, et al. NanoSatellite: Accurate characterization of expanded tandem repeat length and sequence through whole genome long-read sequencing on PromethION. Genome Biology 2019;.
13. Mitsuhashi S, Frith MC, Mizuguchi T, Miyatake S, Toyota T, Adachi H, et al. Tandem-genotypes: robust detection of tandem repeat expansions from long DNA reads. Genome Biology 2019;.
14. Lee C, Grasso C, Sharlow MF. Multiple sequence alignment using partial order graphs. Bioinformatics 2002;.
15. Lee C. Generating consensus sequences from partial order multiple sequence alignment graphs. Bioinformatics 2003;.
16. Tenreiro MacHado JA. Shannon entropy analysis of the genome code. Mathematical Problems in Engineering 2012;.
17. Vaser R, Sović I, Nagarajan N, Šikić M. Fast and accurate de novo genome assembly from long uncorrected reads. Genome Research 2017;.
18. Li H. Minimap2: Pairwise alignment for nucleotide sequences. Bioinformatics 2018;.
19. Bolognini D, Sanders A, Korbel JO, Magi A, Benes V, Rausch T. VISOR: A versatile haplotype-aware structural variant simulator for short-and long-read sequencing. Bioinformatics 2020;.
20. Bolognini D, Bartalucci N, Mingrino A, Vannucchi AM, Magi A. NANOR: A user-friendly R package to analyze and compare nanopore sequencing data. PLoS ONE 2019;.
21. Li H, Handsaker B, Wysoker A, Fennell T, Ruan J, Homer N, et al. The Sequence Alignment/Map format and SAMtools. Bioinformatics 2009;.
22. Rausch T, Hsi-Yang Fritz M, Korbel JO, Benes V. Alfred: Interactive multi-sample BAM alignment statistics, feature counting and feature annotation for long- and short-read sequencing. Bioinformatics 2019;.
23. Pedersen BS, Quinlan AR. Mosdepth: Quick coverage calculation for genomes and exomes. Bioinformatics 2018;.
24. Edge P, Bansal V. Longshot enables accurate variant calling in diploid genomes from single-molecule long read sequencing. Nature Communications 2019;.
25. Mantere T, Kersten S, Hoischen A, Long-read sequencing emerging in medical genetics; 2019.
26. Dolle DD, Liu Z, Cotten M, Simpson JT, Iqbal Z, Durbin R, et al. Using reference-free compressed data structures to analyze sequencing reads from thousands of human genomes. Genome Research 2017;.
27. Ferragina P, Manzini G. Opportunistic data structures with applications. In: Annual Symposium on Foundations of Computer Science - Proceedings; 2000. .
28. Giner-Delgado C, Villatoro S, Lerga-Jaso J, Gayà-Vidal M, Oliva M, Castellano D, et al. Evolutionary and functional impact of common polymorphic inversions in the human genome. Nature Communications 2019;.
29. Mikheenko A, Prjibelski A, Saveliev V, Antipov D, Gurevich A. Versatile genome assembly evaluation with QUAST-LG. In: Bioinformatics; 2018. .
30. Thorvaldsdóttir H, Robinson JT, Mesirov JP. Integrative Genomics Viewer (IGV): High-performance genomics data visualization and exploration. Briefings in Bioinformatics 2013;.
31. Richard GF, Kerrest A, Dujon B. Comparative Genomics and Molecular Dynamics of DNA Repeats in Eukaryotes. Microbiology and Molecular Biology Reviews 2008;.

Figures

Figure 1. TRiCoLOR's P (x-axis), R (y-axis) and F1 (dashed lines) on syntethic TR contractions (**A**) and expansions (**B**). ONT and PB reads exhibit variable error rates (accuracy ~0.85, red; accuracy ~0.90, blue; accuracy ~0.95, green) and were simulated using variable haplotype-specific depth of coverage. P, R and F1 were calculated allowing no motif discrepancies (circle symbol), 1 motif discrepancy (triangle symbol) or 2 motif discrepancies (rhombus symbol) between TRiCoLOR's predictions and the number of repeated motifs in the ground truth.

Figure 2. Correlation results between the number of repeated motifs in the ground truth (x-axis) and the number of repeated motifs predicted by TRiCoLOR and NCRF (y-axis) for synthetic TR contractions (**A**) and expansions (**B**). Each dot represents the synthetic contraction/expansion of a single TR. R is the Pearson's correlation coefficient, p is the p-value of the linear regression analysis, m is the slope of the regression line and the dashed line is the bisector of the first quadrant angle that marks the perfect correspondence between expected and predicted number of TRs.

Manuscript

Click here to access/download;Manuscript;TRiCoLOR_reference_manuscrip

OXFORD (GIGA)^n SCIENCE

GigaScience, 2020, 1–7

doi: xx.xxxx/xxxx
Manuscript in Preparation
Technical Note

TECHNICAL NOTE

TRiCoLOR: tandem repeat profiling using whole-genome long-read sequencing data

Davide Bolognini[1,3,*], Alberto Magi[2], Vladimir Benes[3], Jan O. Korbel[4] and Tobias Rausch[3,4]

[1]Department of Experimental and Clinical Medicine, University of Florence, Florence, 50134, Italy and [2]Department of Information Engineering, University of Florence, Florence, 50134, Italy and [3]European Molecular Biology Laboratory (EMBL), GeneCore, Heidelberg, 69117, Germany and [4]European Molecular Biology Laboratory (EMBL), Genome Biology Unit, Heidelberg, 69117, Germany

*davidebolognini7@gmail.com

Abstract

Background: Tandem repeat sequences are widespread in the human genome and their expansions cause multiple repeat-mediated disorders. Genome-wide discovery approaches are needed to fully understand their roles in health and disease but resolving tandem repeat variation accurately remains a very challenging task. While traditional mapping-based approaches using short-read data have severe limitations in the size and type of tandem repeats they can resolve, recent third-generation sequencing technologies exhibit substantially higher sequencing error rates which complicates repeat resolution.
Results: We developed TRiCoLOR, a freely-available tool for tandem repeat profiling using error-prone long reads from third-generation sequencing technologies. The method can identify repetitive regions in sequencing data without a prior knowledge of their motifs or locations and resolve repeats multiplicity and period size in a haplotype-specific manner. The tool includes methods to interactively visualize the identified repeats and to trace their Mendelian consistency in pedigrees.
Conclusions. TRiCoLOR demonstrates excellent performance and improved sensitivity and specificity compared to alternative tools on synthetic data. For real human whole-genome sequencing data, TRiCoLOR achieves high validation rates suggesting its suitability to identify tandem repeat variation in personal genomes.

Key words: long-read sequencing; tandem repeat variation; bioinformatics software

Background

Almost half of the human genome is estimated to be covered by repetitive sequences [1]. Among these, tandem repeats (TR) have been found to be involved in a range of functions such as DNA repair, chromatin organization, telomere maintenance, and regulation of gene expression [2]. Most importantly, more than 40 diseases, primarily neurological, are known to be related to TR expansions [3]. Despite their clinical importance, accurately resolving TRs remains challenging in sequencing data sets mainly because of insufficient read lengths failing to encompass entire expanded repeats or technological limitations, such as high sequencing error-rates.

Prior methods for TR profiling in short-read sequencing data sets can be broadly classified as reference-based [4, 5] or *de novo* [6, 7] approaches. While the former investigates only reads spanning known TRs, the latter can identify TRs regardless of whether their repeat motif is annotated or not in the reference. Short read methods are often inadequate to accurately resolve expanded TRs if the total repeat length is greater than the read length.

Long reads from third-generation sequencing technologies,

Compiled on: August 7, 2020.
Draft manuscript prepared by the author.

namely Oxford Nanopore Technologies (ONT) and Pacific Biosciences (PB), have proved already invaluable for the discovery of large structural variants [8] and are obvious candidates for broadening the scope of detectable TRs. However, long reads exhibit high sequencing error rates which make it difficult to accurately decipher TRs, especially in low complexity regions.
Few TR detection methods for long-read sequencing data have been developed so far. Examples include PacmonSTR [9], NCRF [10], TideHunter [11], NanoSatellite [12] and Tandem-genotypes [13]. However, these tools have some limitations, either because they are technology-specific (PacmonSTR and NanoSatellite), because they are not intended to be used genome-wide (NCRF, TideHunter and NanoSatellite) or because they require substantial preprocessing steps preventing their large-scale use (Tandem-genotypes). Some tools also lack genotyping capabilities (NCRF and TideHunter) and none of the aforementioned methods is capable to profile TRs *de novo* in regions that have previously not been annotated as harboring a TR.
TRiCoLOR addresses these shortcomings of existing, alignment-based tools by allowing users to rapidly identify and genotype TRs from haplotype-resolved long-read alignments. Once low-entropy repetitive regions have been identified in sequenced long reads, TRiCoLOR exploits partial order alignment (POA) [14] to compute haplotype-specific low-error consensus sequences [15] that are further processed by means of a fast regular expression (RegEx)-based approximate string matching algorithm to resolve repeat motif and multiplicity of the discovered TRs. Detected TRs can be interactively visualized within their haplotype-specific sequence context for manual exploration of expanded or contracted repeats. For trio sequencing studies, TRiCoLOR additionally allows to trace Mendelian inheritance patterns across TR genotypes.

Methods

TRiCoLOR (Tandem Repeats Caller for LOng Reads) requires haplotype-resolved long-read alignments as input (Supplementary Note S1). It then runs a series of modules to identify and genotype TRs as described in detail below. A manual containing an in-depth explanation of how to install TRiCoLOR and run its various modules is available at https://davidebolo1993.github.io/tricolordoc, including use case examples.

Identifying repetitive regions *de novo*

TRiCoLOR can identify repetitive regions in haplotype-resolved BAM files *de novo*. This is achieved using the SENSoR (Shannon ENtropy ScanneR) module, which uses the Shannon entropy of DNA sequences to identify candidate repetitive segments in genomic sequences [16]. TRiCoLOR SENSoR scans in parallel the haplotype-specific BAM files and computes, for each sequencing read, its Shannon entropy content in non-overlapping, sliding windows of a pre-trained size (20 bps, by default). Genomic coordinates of windows in which multiple reads ($\geq$5, by default) support an entropy drop ($\leq$ 1.23, by default) are stored and those nearby are merged (those falling within 100 bps intervals, by default). The default entropy treshold of 1.23 efficiently discriminates between repetitive and non-repetitive DNA sequences using syntethic ONT and PB reads as shown in Supplementary Figure S1 (see Supplementary Note S2). All candidate repetitive regions identified with this approach are eventually outputted in BED format. This pre-filtering of repetitive regions is fairly fast even in deep-coverage whole-genome data (see also *Findings*) and drastically reduces the computational time required for the subsequent TR profiling.

Profiling repetitive regions

TRiCoLOR can profile TRs in haplotype-resolved BAM files through the REFER (REpeats FindER) module. The input of REFER is a BED file generated by TRiCoLOR SENSoR. Alternatively, the BED file can be provided by the user based on prior knowledge of clinically relevant TRs, for instance.
For each region in the BED file, REFER first fetches from the haplotype-specific BAM files the sequencing reads spanning the selected region and trims them, so that the length of each read is approximately the size of the region. Let $R = [S, E]$ be a region from the BED file, ranging from a start coordinate S to an end coordinate E for a given chromosome. Each sequencing read entirely spanning R is fetched and trimmed so that the actual sequence REFER stores is that included between S and E, which significantly improves the runtime of the subsequent POA algorithm to generate a consensus sequence.
Once the sequencing reads of interest have been collected and trimmed, TRiCoLOR uses SPOA [17], a single-instruction multiple-data accelerated version of the robust POA framework, to compute highly accurate consensus sequences with an approximate error reduction of ~77% and ~88% for ONT and PB, respectively (Supplementary Note S3 and Supplementary Figure S2).
With the haplotype-specific consensus sequences at hand, REFER aligns these to the reference genome using minimap2 [18], which compared favorably to alternative aligners on syntethic data, both in terms of speed and mapping accuracy (Supplementary Note S4 and Supplementary Figure S3). The reference-aligned low-error consensus sequences are then screened by a RegEx-based approximate string matching algorithm, which has three processing steps: (1) identifying motifs (motifs of length $\leq$ 6, by default) that are perfectly repeated a minimum number of times (5, by default); (2) looking for approximate repetitions of the identified motifs to account for remaining consensus errors, that is imperfect repeated motifs up to a user-defined edit distance ($\leq$ 1, by default); (3) in case of multiple overlapping approximate repetitions, resolving these competing tandem repeat predictions using an N-gram model that favors the most frequently occurring perfect repeat motif.
Together with the haplotype-specific consensus sequences, the corresponding reference is screened in a similar manner, with few differences being noteworthies: (1) the algorithm assumes the reference does not contain errors and does not look for approximate repetitions of the motifs identified; (2) among overlapping repetitions, the longest repeat is taken.
TRs (those $\geq$ 50 bps, by default) varying between the haplotypes or the reference are eventually stored in BCF-compliant format. TRiCoLOR REFER also stores in the output folder several BED files describing the TRs identified (both for the reference and each haplotype) and haplotype-specific BAM files containing the aligned consensus sequences.

Visualizing identified repeats

The TRs profiled using TRiCoLOR REFER can be interactively visualized through the ApP (Alignment Plotter) module. This module takes as inputs the BED and the BAM files generated by TRiCoLOR REFER together with an additional BED file describing one or more regions to plot.
TRiCoLOR ApP produces a static HTML file illustrating the alignment between the reference and the individual's haplotypes at single base resolution, highlighting the TRs detected. (Supplementary Note S5 and Supplementary Figures S4A-S4C).

Tracing Mendelian inheritance patterns of identified repeats

In pedigree studies, assigned genotypes can be either Mendelian consistent or inconsistent. TRiCoLOR enables genotype consistency checks for TRs identified in the index child when haplotype-resolved long-read alignments for both parents are available. This is achieved through the SAGE (SAmple GEnotyper) module with special emphasis on the common situation that parents have been sequenced at low depth.
Using the same aforementioned TRiCoLOR REFER approach, SAGE computes haplotype-specific consensus alignments for each child TR in each parent. Next the module checks whether the parental TRs are more similar (*i.e.*, have a lower edit distance) to the reference or to the TR identified in the child and assigns them the most likely genotype. Knowing the genotype of both parents, the module eventually flags each TR as Mendelian consistent or inconsistent with the −*mendel* parameter enabled. The output of TRiCoLOR SAGE is a multi-sample BCF file that contains the genotypes for the index child and both parents.

Findings

We benchmarked TRiCoLOR using both synthetic data generated with VISOR [19] and real, publicly available data from the Human Genome Structural Variation Consortium (HGSVC) [8].

Benchmarking TRiCoLOR on synthetic data

We used the TR simulator VISOR to generate synthetic ONT and PB alignments containing TR contractions and expansions. First, we simulated haplotype-resolved ONT and PB BAM files (the average length of simulated reads was set to 8000 bps based on statistics derived from recent ONT sequencing runs [20]; the substitution:insertion:deletion ratio was set to ~45:25:30 for the synthetic ONT reads and to ~15:50:35 for the synthetic PB reads, in accordance with findings described in Supplementary Note S3) exhibiting variable error rates (accuracy of reads ~0.85, ~0.90 and ~0.95) and depth of coverage (haplotype-specific depth of coverage 5X-10X and 10X-20X),with each BAM file harboring a heterozygous contraction or expansion of a known, randomly chosen, TR. At this stage, we simulated small TR contractions/expansions (contractions/expansions of 7 motifs on average) in order to evaluate the capability of our method to spot even minor changes in the TR multiplicity of the 2 haplotypes. For each group, we simulated 200 haplotype-resolved BAM files. Then, we evaluated the performances of TRiCoLOR in terms of precision (P), recall (R), and F1 score (F1) (Supplementary Note S6). In particular, P, R and F1 values were calculated allowing no discrepancies, 1 discrepancy or 2 discrepancies between the number of repeated motifs in the ground truth and the number of repeated motifs predicted by TRiCoLOR. Figure 1 shows these findings for synthetic TR contractions (panel A) and expansions (panel B). TRiCoLOR demonstrated high P and R in all the simulated groups: our method always achieved an F1 close to 1 when allowing a single-motif discrepancy between simulated and predicted TRs and hit P ~1 and R ~1 when allowing up to 2 motif discrepancies. For both contractions and expansions the F1 depends on the coverage and input read accuracy as expected. In all the simulated TR contractions and expansions, TRiCoLOR was also able to properly identify the correct repeated motif, few times shifted (*e.g.*, a repeated TG instead of a repeated GT). Supplementary Figure S5 illustrates these findings for the same simulated groups of Figure 1, averaged over the different accuracy levels.

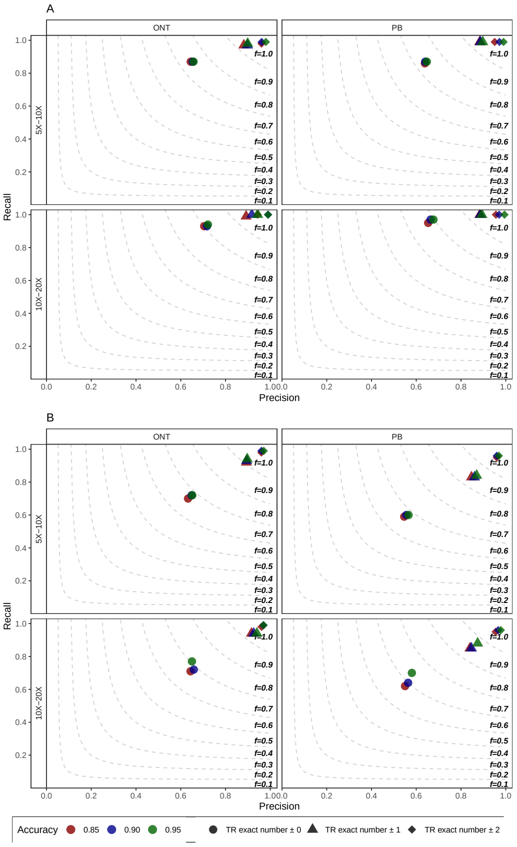

Figure 1. TRiCoLOR's P (x-axis), R (y-axis) and F1 (dashed lines) on syntethic TR contractions (**A**) and expansions (**B**). ONT and PB reads exhibit variable error rates (accuracy ~0.85, red; accuracy ~0.90, blue; accuracy ~0.95, green) and were simulated using variable haplotype-specific depth of coverage. P, R and F1 were calculated allowing no motif discrepancies (circle symbol), 1 motif discrepancy (triangle symbol) or 2 motif discrepancies (rhombus symbol) between TRiCoLOR's predictions and the number of repeated motifs in the ground truth.

Furthermore, as a proof of concept, we compared TRiCoLOR to a TR caller for long reads recently published, namely NCRF. Using the same approach described above, we simulated 100 ONT and 100 PB BAM files (accuracy of reads ~0.90, depth of coverage for each haplotype 5X-10X), each harboring a small TR contraction/expansion and we run both TRiCoLOR and NCRF on these data. As NCRF cannot deal with BAM input, we slightly modified TRiCoLOR to store in FASTA format the sequences used for the consensus computation step, which could be processed through NCRF (Supplementary Note S7). Figure 2 shows the correlation results between the number of repeated motifs in the ground truth and the number of repeated motifs predicted by TRiCoLOR and NCRF for the simulated TR contractions (panel A) and expansions (panel B). For both TR contractions and expansions, TRiCoLOR got excellent R scores (R = 0.97 for contractions and R = 0.86 for expansions), outperforming NCRF (R = 0.87 for contractions and R = 0.74 for expansions). We next evaluated exceptionally long TR expansions because these have been implicated in several neurological disorders. For instance, the common Fragile-X Syndrome is related to a CGG-repeat usually consisting of ≤55 repeated motifs that expands to ≥ 200 repeated motifs. Following the simulation schema described above, we generated 100 ONT and 100 PB synthetic BAM files harboring TRs expanded by 200 motifs and we run both TRiCoLOR and NCRF on these data. Supplementary

Figure S6 shows the correlation results between the number of repeated motifs in the ground truth and the number of repeated motifs predicted by TRiCoLOR and NCRF for the simulated long TR expansions. As above, TRiCoLOR achieved the best R score (R = 0.73), outperforming NCRF (R = 0.53).

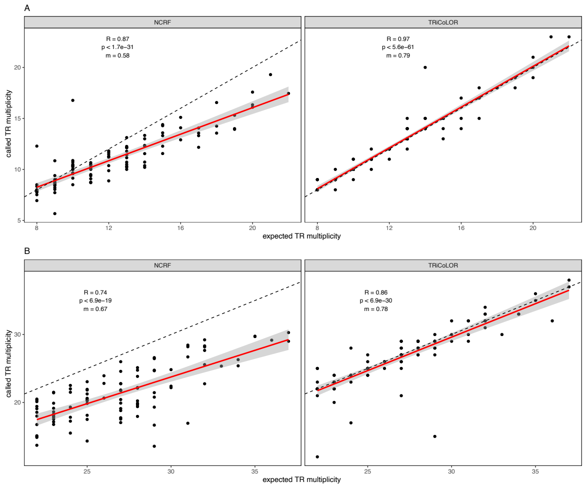

Figure 2. Correlation results between the number of repeated motifs in the ground truth (x-axis) and the number of repeated motifs predicted by TRiCoLOR and NCRF (y-axis) for syntethic TR contractions (**A**) and expansions (**B**). Each dot represents the synthetic contraction/expansion of a single TR. R is the Pearson's correlation coefficient, p is the p-value of the linear regression analysis, m is the slope of the regression line and the dashed line is the bisector of the first quadrant angle that marks the perfect correspondence between expected and predicted number of TRs.

Benchmarking TRiCoLOR on real data

We applied TRiCoLOR to call TRs *de novo* on publicly available ONT and PB human whole-genome sequencing data from the HGSVC project. In particular, we used the ONT sequencing data for HG00514 (Han Chinese), HG00733 (Puerto Rican) and NA19240 (Yoruban Nigerian) and the PB sequencing data for HG00731 (Puerto Rican, father), HG00732 (Puerto Rican, mother) and HG00733 (son).

We aligned the ONT FASTQ files to the human GRCh38 reference genome using minimap2 and we merged the chromosome-specific PB alignments using samtools [21]. We then split the ONT and PB alignments by haplotype with Alfred [22] using phased single-nucleotide variants from the HGSVC project. We calculated the coverage of the initial and the haplotype-resolved BAM files using mosdepth [23]. For all the ONT samples, we identified an initial ~20X coverage (HG00733 ~21X, HG00514 ~23X and NA19240 ~24X), slightly reduced after splitting by haplotype due to some unassigned reads (HG00733 ~8X, HG00514 ~9X and NA19240 ~10X for each haplotype). For the PB samples, we identified a ~42X coverage for HG00733 and ~21X coverage for HG00731 and HG00732, reduced after splitting the data by haplotype (HG00733 ~14X, HG00731 and HG00732 ~8X for each haplotype).

We then run TRiCoLOR SENSoR using the default parameter settings on the HG00733 (ONT and PB), HG00514 and NA19240 individuals. Using an Ubuntu 16.04.6 LTS desktop with Intel®Xeon®processors X5460 (clock rate 2.93 GHz), the module took ~4 hours to scan the ONT samples and ~8 hours to scan the PB sample, which reflects the higher coverage available for PB. For the HG00733, HG00514 and NA19240 ONT individuals the module identified ~160000, ~190000 and ~260000 low-entropy regions (average length of the regions ~900 bps), which were reduced to ~70000, ~100000 and ~160000 respectively after filtering for regions with average coverage > 8. For the HG00733 PB individual the module identified ~380000 low-entropy regions (average length of the regions ~850 bps), which were reduced to ~150000, after filtering for regions with average coverage > 10. For HG00733, ~97% of the low-entropy regions originally identified in the ONT individual overlapped those in the PB one; due to the different coverage distributions, this percentage was reduced to ~31% after filtering.

We run TRiCoLOR REFER on the samples processed by TRiCoLOR SENSoR using the default parameter settings. With 7 processors on our Ubuntu desktop, the module took ~10-12 hours to profile TRs on the ONT individuals and ~14 hours to profile TRs on the PB individual.

We calculated the number of TRs properly called by TRiCoLOR using an alignment-free validation approach. Current benchmarks for TR calling in human genomes are mainly based on short-read sequencing and are biased towards regions of the genome that are easy-to-call with such a technology [24]. It has been shown that it is often impossible to accurately map or even assemble short reads originating from repetitive regions [25], and as a consequence of this, some TRs are missing from the available TR callsets. Following the idea from Dolle *et al.* [26] we first built full-text searchable FM indexes [27] both for the GRCh38 human reference FASTA and the high-quality Illumina FASTQ files of the HG00733, HG00514 and NA19240 individuals. Then, for each individuals' variant identified by TRiCoLOR REFER, the validation algorithm: (1) checks if the variant sequence appears one or more times in the reference FM index: if so, using the consensus BAM files stored by TRiCoLOR REFER, the variant sequence is extended by 1 bp to the left and 1 bp to the right and step 1 is repeated; if not, the algorithm proceeds to the next step; (2) checks if the variant appears one or more times in the corresponding Illumina FM index: if so, the variant is considered a valid call; if not, the variant is considered an invalid call. Taking into account possible errors both in the consensus sequences generated by TRiCoLOR and in the Illumina sequences, we counted as valid calls also variants that are found in the Illumina FM indexes with up to 2 bp discrepancies (*i.e.* their edit distance is ≤ 2). Limited by the length of the available Illumina sequences, using this approach we could not validate variant TRs longer than 124 bps. Overall, we got high validation ratios (ratios between the valid calls and the number of calls that could be assessed using short reads): ~82% for HG00733 (ONT and PB), ~85% for HG00514 and ~86% for NA19240 (Supplementary Figure S7).

We eventually run TRiCoLOR SAGE on the Puerto Rican PB trio HG00731, HG00732 and HG00733, with the default parameter settings and the *−mendel* parameter enabled to check the Mendelian consistency of the TRs identified in HG00733. With 7 processors on our Ubuntu desktop, the module took ~2 hours to complete the analysis. Filtering for variants differing from the reference for at least 10 bps and for multi-allelic variants differing from each other by the same distance, we identified ~80% of Mendelian consistent TRs, which is low compared to trio-based single-nucleotide variant and InDel Mendelian consistency rates, but above reported genotype agreement rates for structural variants in repetitive regions [28].

Among the Mendelian consistent TRs called by TRiCoLOR on the HG00733 PB individual, we identified 32 long TRs (≥ 150 bps) that were absent in the HGSVC ground truth for the same individual. In order to identify the cause of these apparent discrepancies, we aligned the HG00733 phased contigs from HGSVC to the GRCh38 human reference genome with minimap2, using the assembly-to-reference alignment mode and the parameters suggested by QUAST-LG [29] and we manually inspected the discordant TRs in the aligned contigs using IGV

Table 1. Comparison between TRiCoLOR's mapping-based and HGSVC's assembly-based approaches for Mendelian consistent long TRs identified by TRiCoLOR on the HG0733 PB individual.

chromosome	start	end	HGSVC assembly*	TRiCoLOR call*
chr1	23703657	23703893	DEL;INS	DEL;INS
chr1	223672571	223672681	INS;INS	INS;INS
chr10	69539376	69539572	INS;INS	INS;INS
chr11	79190887	79191145	**REF;REF**	**DEL;INS**
chr11	128436913	128437081	INS;INS	INS;INS
chr14	84276747	84276903	**REF**;DEL	**INS**;DEL
chr15	70364402	70364587	INS;**NA**	INS;**INS**
chr16	3529535	3529854	REF;**DEL**	LC;**INS**
chr17	27525992	27526118	INS;INS	INS;INS
chr18	44544809	44545037	INS;INS	INS;INS
chr18	59081301	59081379	INS;**INS**	INS;**INS**
chr18	71198388	71198450	REF;**NA**	REF;**INS**
chr2	160426201	160426342	INS;INS	INS;INS
chr2	211860947	211861156	DEL;**NA**	DEL;**INS**
chr21	35063465	35063588	INS;INS	INS;INS
chr22	46174187	46174274	REF;INS	REF;INS
chr3	13856835	13857013	DEL;INS	DEL;INS
chr4	13807826	13807982	REF;**REF**	REF;**INS**
chr4	18837113	18837320	INS;DEL	INS;DEL
chr4	81637241	81637408	DEL;DEL	DEL;DEL
chr5	54513584	54513735	**REF**;INS	**INS**;INS
chr6	25450910	25450975	REF;**INS**	REF;**INS**
chr6	55543085	55543393	INS;INS	INS;INS
chr6	106945844	106946002	DEL;**DEL**	DEL;**INS**
chr7	38610247	38610412	**NA**;DEL	**INS**;DEL
chr7	71847696	71847865	INS;INS	INS;INS
chr7	109663557	109663744	INS;DEL	INS;DEL
chr7	131933466	131933651	INS;INS	INS;INS
chr9	82850174	82850347	DEL;DEL	DEL;DEL
chr9	91622218	91622365	**NA**;NA	**INS**;REF
chr9	91634814	91634973	**NA;NA**	**DEL;INS**
chr9	116632126	116632280	INS;INS	INS;INS

* DEL indicates a deletion; INS indicates an insertion; REF indicates a reference allele; NA indicates that the region is not covered by the assembly or mis-assembled; LC indicates that TRiCoLOR could not generate a consensus sequence for the allele due to the low coverage in the region. The 2 alleles are separated by a semicolon.

[30]. As shown in Table 1, out of 58 non-reference TR alleles identified by TRiCoLOR, we could visually confirm 42 (~75%) of them in the HGSVC assembly, which means that both TRiCoLOR and the HGSVC predicted the same variant type (deletion or insertion) and the predicted variant size is roughly similar (*i.e.* the difference does not exceed 50 bps). However, for the other 16 variants (~25%), the HGSVC assembly either did not contain the allele predicted by TRiCoLOR or did not cover the investigated region, which suggests that mapping-based and assembly-based approaches can be complementary for TR detection using long reads.

Discussion

TRiCoLOR is a comprehensive TR caller for long reads that supports the *de novo* identification of TRs in whole-genome sequencing data. TRiCoLOR profiles TRs through an efficient POA algorithm combined with a RegEx-based string matching search, facilitating a robust and accurate discovery of the full spectrum of expanded and contracted TRs in personal genomes.

In comparison to previous tools, TRiCoLOR works with ONT and PB data seamlessly. TRiCoLOR also identifies TRs *de novo* and does not require *a priori* knowledge of annotated TR regions. The unique combination of features for genome-wide, *de novo* discovery and genotyping of TRs in ONT and PB data is to the best of our knowledge unmet by any other TR caller for long-read data. Besides the detection of TRs, TRiCoLOR visualizes TRs in their haplotype context and it can infer parental genotypes using low-coverage parental sequencing data.

TRiCoLOR has been designed for diploid organisms (Supplementary Note S8) and future work includes extending its feature set to polyploid species and haploid chromosomes (human Y chromosome). As a mapping-based approach, TRiCoLOR cannot identify repeats in unassembled regions of the genome (*e.g.*, human centromeres and telomeres). Furthermore, the entropy threshold and window size for the *de novo* identification of repetitive stretches that we empirically estimated is well-suited for short repeated motifs (2–3 bps) but may need adjustments for long motifs of higher nucleotide complexity. Lastly, by default TRiCoLOR profiles TRs with motif lengths $\leq$ 6 bps (also known as micro-satellites), excluding those with motif lengths $\geq$7 bps (also known as mini-satellites), which are less abundant in diploid organisms [31]. The RegEx algorithm can be also tuned to profile mini-satellites (*i.e.*, by extending the –*size* parameter) but TRiCoLOR has been extensively applied so far only to micro-satellites.

Given these limitations, future work will focus on extending TRiCoLOR to other ploidies, broadening the size spectrum of detectable repeat motif lengths and taking advantage of improved sequencing read accuracy (*e.g.*, high-fidelity long reads from PB). The latter directly improves the RegEx-based identification of repeats employed by TRiCoLOR and we thus believe TRiCoLOR is well-suited to characterize the TR landscape in present and future long-read data sets, making it an instrumental tool to robustly decipher the multiplicity of TRs in repeat-mediated clinical disorders.

Availability of source code and requirements

- Project name: TRiCoLOR (SciCrunch RRID: SCR_018801; bio.tools ID: tricolor)
- Project home page: https://github.com/davidebolo1993/TRiCoLOR. A dockerized version of TRiCoLOR is available at https://hub.docker.com/r/davidebolo1993/tricolor. Online documentation is available at https://davidebolo1993.github.io/tricolordoc.
- Operating system: Unix
- Programming languages: Python, Bash, C++
- Other requirements: Python 3.6 or higher, GCC 4.8 or higher and CMake 3.2 or higher.
- License: GNU Lesser General Public License 3.0

Availability of supporting data and materials

HGSVC whole-genome long-read sequencing data are available on the HGSVC website (https://www.internationalgenome.org/human-genome-structural-variation-consortium). Specifically:

- ONT FASTQ files: http://ftp.ebi.ac.uk/1000g/ftp/data_collections/hgsv_sv_discovery/working/20181210_ONT_rebasecalled
- PB alignments: http://ftp.ebi.ac.uk/1000g/ftp/data_collections/hgsv_sv_discovery/working/20180102_pacbio_blasr_reheader
- Phased single-nucleotide variants: http://ftp.ebi.ac.uk/1000g/ftp/data_collections/hgsv_sv_discovery/working/20170323_Strand-seq_phased_FB%2BGATK_VCFs
- Illumina FASTQ files: http://ftp.ebi.ac.uk/1000g/ftp/data_collections/hgsv_sv_discovery/illumina_wgs.sequence.index
- HG00733 phased contigs: http://ftp.ebi.ac.uk/1000g/ftp/data_collections/hgsv_sv_discovery/working/20180227_PhasedSVGenomes
- HG00733 ground truth of structural variant calls:

http://ftp.ebi.ac.uk/1000g/ftp/data_collections/hgsv_sv_discovery/working/20180627_PanTechnologyIntegrationSet/HG00733.merged_nonredundant.vcf

The GRCh38 human reference genome used for alignments is available at http://ftp.ebi.ac.uk/1000g/ftp/technical/reference/GRCh38_reference_genome/GRCh38_full_analysis_set_plus_decoy_hla.fa. The corresponding annotated TRs can be accessed through the UCSC Table Browser tool (http://genome.ucsc.edu).
A whole-genome ONT FASTQ file of the *Arabisopsis thaliana* KBS-Mac-74 is available at ftp://ftp.sra.ebi.ac.uk/vol1/fastq/ERR217/003/ERR2173373/ERR2173373.fastq.gz. The TAIR10 reference genome for *Arabidopsis thaliana* can be downloaded through the Arabidopsis Information Resource database (https://www.arabidopsis.org/index.jsp) Several scripts used to perform the analyses described in this paper and the TR calls generated by TRiCoLOR for the HGSVC individuals and the *Arabisopsis thaliana* KBS-Mac-74 are available through the GitHub code repository of TRiCoLOR (https://github.com/davidebolo1993/TRiCoLOR). More in detail:

- the https://github.com/davidebolo1993/TRiCoLOR/tree/master/paper/data folder contains the BED file with annotated TRs from the GRCh38 human reference genome (`GRCh38.TRs.bed`), a bash script that illustrates how to haplotype-resolve a long-read alignment using phased single-nucleotide variants (`prepare.sh`), a python script used for the Shannon entropy simulations (`entropy.py`), a python script used to calculate precision, recall and F1 scores of TRiCoLOR on synthetic data (`pr.py`) and a couple of C++ source code files (`fmindex.cpp` and `validate.cpp`) for validating TRiCoLOR calls on real human data.
- the https://github.com/davidebolo1993/TRiCoLOR/tree/master/paper/samples folder contains TRiCoLOR calls for the HGSVC individuals and the *Arabisopsis thaliana* KBS-Mac-74 in standard BCF format.

Declarations

List of abbreviations

TR: tandem repeat; ONT: Oxford Nanopore Technologies; PB: Pacific Biosciences; POA: partial order alignment; RegEx: regular expression; HGSVC: Human Genome Structural Variation Consortium; P: precision; R: recall; F1: F1 score.

Competing Interests

The authors declare that they have no competing interests.

Funding

JOK is supported by GraphGenomes grant 031L0184C. AM is supported by AIRC grant 20307. The funders had no role in study design, data collection and analysis, decision to publish, or preparation of the manuscript.

Author's Contributions

DB and TR designed and benchmarked the software. DB wrote the code. TR supervised the work. DB and TR co-wrote the manuscript draft. AM, VB and JOK contributed to the interpretation of the results, provided critical feedback and helped to write the manuscript. All the authors read and approved the manuscript.

Acknowledgements

The authors thank HGSVC for data access, and EMBL GeneCore and IT for technical support.

References

1. de Koning APJ, Gu W, Castoe TA, Batzer MA, Pollock DD. Repetitive elements may comprise over Two-Thirds of the human genome. PLoS Genetics 2011;.
2. Hannan AJ, Tandem repeats mediating genetic plasticity in health and disease; 2018.
3. Paulson H. Repeat expansion diseases. In: Handbook of Clinical Neurology; 2018.
4. Gymrek M, Golan D, Rosset S, Erlich Y. lobSTR: A short tandem repeat profiler for personal genomes. Genome Research 2012;.
5. Highnam G, Franck C, Martin A, Stephens C, Puthige A, Mittelman D. Accurate human microsatellite genotypes from high-throughput resequencing data using informed error profiles. Nucleic Acids Research 2013;.
6. Doi K, Monjo T, Hoang PH, Yoshimura J, Yurino H, Mitsui J, et al. Rapid detection of expanded short tandem repeats in personal genomics using hybrid sequencing. Bioinformatics 2014;.
7. Velasco A, James BT, Wells VD, Girgis HZ, Elofsson A. Look4TRs: A de novo tool for detecting simple tandem repeats using self-supervised hidden Markov models. Bioinformatics 2020;.
8. Chaisson MJP, Sanders AD, Zhao X, Malhotra A, Porubsky D, Rausch T, et al. Multi-platform discovery of haplotype-resolved structural variation in human genomes. Nature Communications 2019;.
9. Ummat A, Bashir A. Resolving complex tandem repeats with long reads. Bioinformatics 2014;.
10. Harris RS, Cechova M, Makova KD, Birol I. Noise-cancelling repeat finder: Uncovering tandem repeats in error-prone long-read sequencing data. Bioinformatics 2019;.
11. Gao Y, Liu B, Wang Y, Xing Y. TideHunter: Efficient and sensitive tandem repeat detection from noisy long-reads using seed-and-chain. In: Bioinformatics; 2019. .
12. De Roeck A, De Coster W, Bossaerts L, Cacace R, De Pooter T, Van Dongen J, et al. NanoSatellite: Accurate characterization of expanded tandem repeat length and sequence through whole genome long-read sequencing on PromethION. Genome Biology 2019;.
13. Mitsuhashi S, Frith MC, Mizuguchi T, Miyatake S, Toyota T, Adachi H, et al. Tandem-genotypes: robust detection of tandem repeat expansions from long DNA reads. Genome Biology 2019;.
14. Lee C, Grasso C, Sharlow MF. Multiple sequence alignment using partial order graphs. Bioinformatics 2002;.
15. Lee C. Generating consensus sequences from partial order multiple sequence alignment graphs. Bioinformatics 2003;.
16. Tenreiro MacHado JA. Shannon entropy analysis of the genome code. Mathematical Problems in Engineering 2012;.
17. Vaser R, Sović I, Nagarajan N, Šikić M. Fast and accurate de novo genome assembly from long uncorrected reads. Genome Research 2017;.
18. Li H. Minimap2: Pairwise alignment for nucleotide sequences. Bioinformatics 2018;.
19. Bolognini D, Sanders A, Korbel JO, Magi A, Benes V, Rausch T. VISOR: A versatile haplotype-aware structural variant simulator for short-and long-read sequencing. Bioinfor-

matics 2020;.
20. Bolognini D, Bartalucci N, Mingrino A, Vannucchi AM, Magi A. NANOR: A user-friendly R package to analyze and compare nanopore sequencing data. PLoS ONE 2019;.
21. Li H, Handsaker B, Wysoker A, Fennell T, Ruan J, Homer N, et al. The Sequence Alignment/Map format and SAMtools. Bioinformatics 2009;.
22. Rausch T, Hsi-Yang Fritz M, Korbel JO, Benes V. Alfred: Interactive multi-sample BAM alignment statistics, feature counting and feature annotation for long- and short-read sequencing. Bioinformatics 2019;.
23. Pedersen BS, Quinlan AR. Mosdepth: Quick coverage calculation for genomes and exomes. Bioinformatics 2018;.
24. Edge P, Bansal V. Longshot enables accurate variant calling in diploid genomes from single-molecule long read sequencing. Nature Communications 2019;.
25. Mantere T, Kersten S, Hoischen A, Long-read sequencing emerging in medical genetics; 2019.
26. Dolle DD, Liu Z, Cotten M, Simpson JT, Iqbal Z, Durbin R, et al. Using reference-free compressed data structures to analyze sequencing reads from thousands of human genomes. Genome Research 2017;.
27. Ferragina P, Manzini G. Opportunistic data structures with applications. In: Annual Symposium on Foundations of Computer Science - Proceedings; 2000. .
28. Giner-Delgado C, Villatoro S, Lerga-Jaso J, Gayà-Vidal M, Oliva M, Castellano D, et al. Evolutionary and functional impact of common polymorphic inversions in the human genome. Nature Communications 2019;.
29. Mikheenko A, Prjibelski A, Saveliev V, Antipov D, Gurevich A. Versatile genome assembly evaluation with QUAST-LG. In: Bioinformatics; 2018. .
30. Thorvaldsdóttir H, Robinson JT, Mesirov JP. Integrative Genomics Viewer (IGV): High-performance genomics data visualization and exploration. Briefings in Bioinformatics 2013;.
31. Richard GF, Kerrest A, Dujon B. Comparative Genomics and Molecular Dynamics of DNA Repeats in Eukaryotes. Microbiology and Molecular Biology Reviews 2008;.

Figure 1

Click here to access/download;Figure;Fig1.pdf

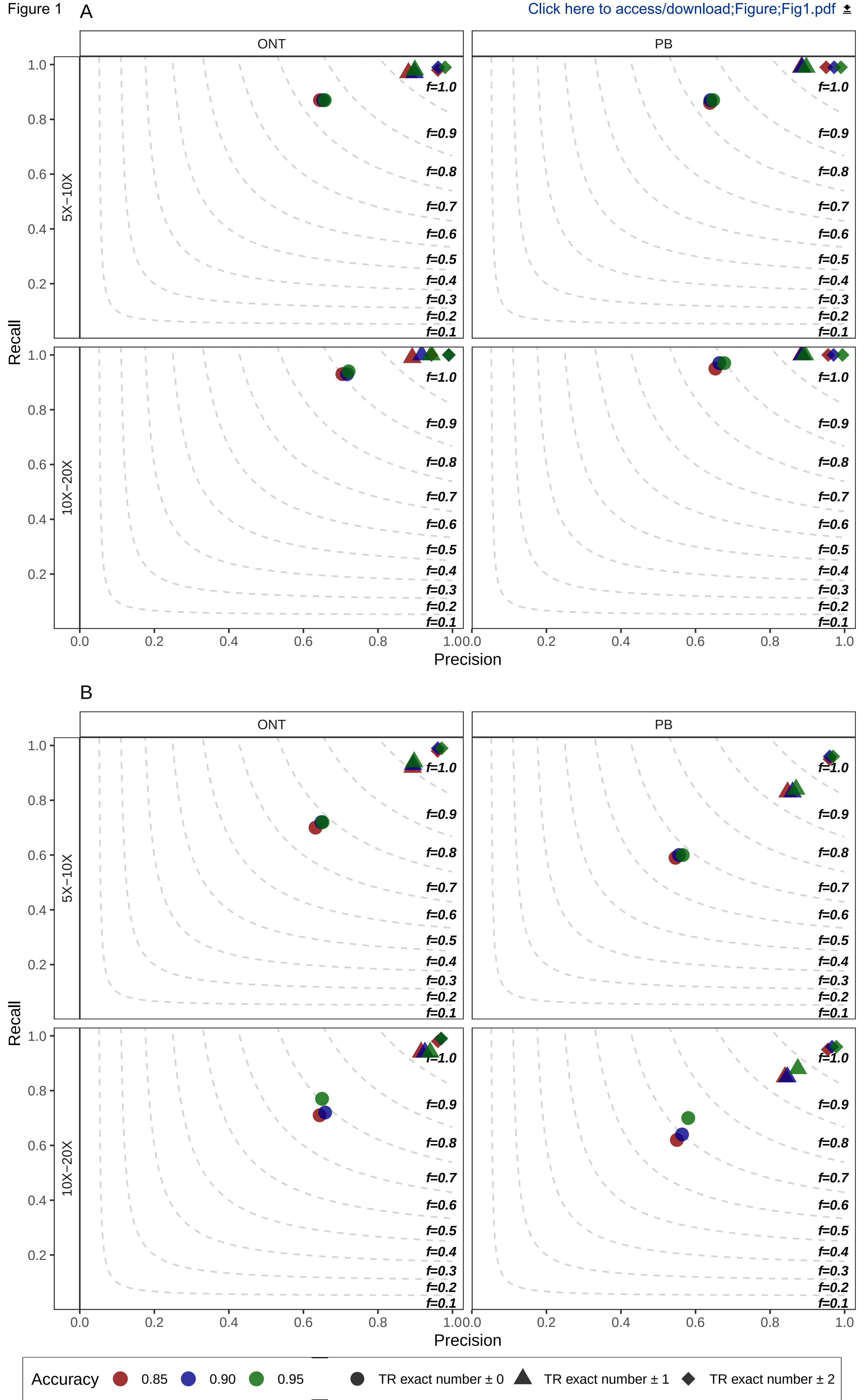

Click here to access/download;Figure;Fig2.pdf

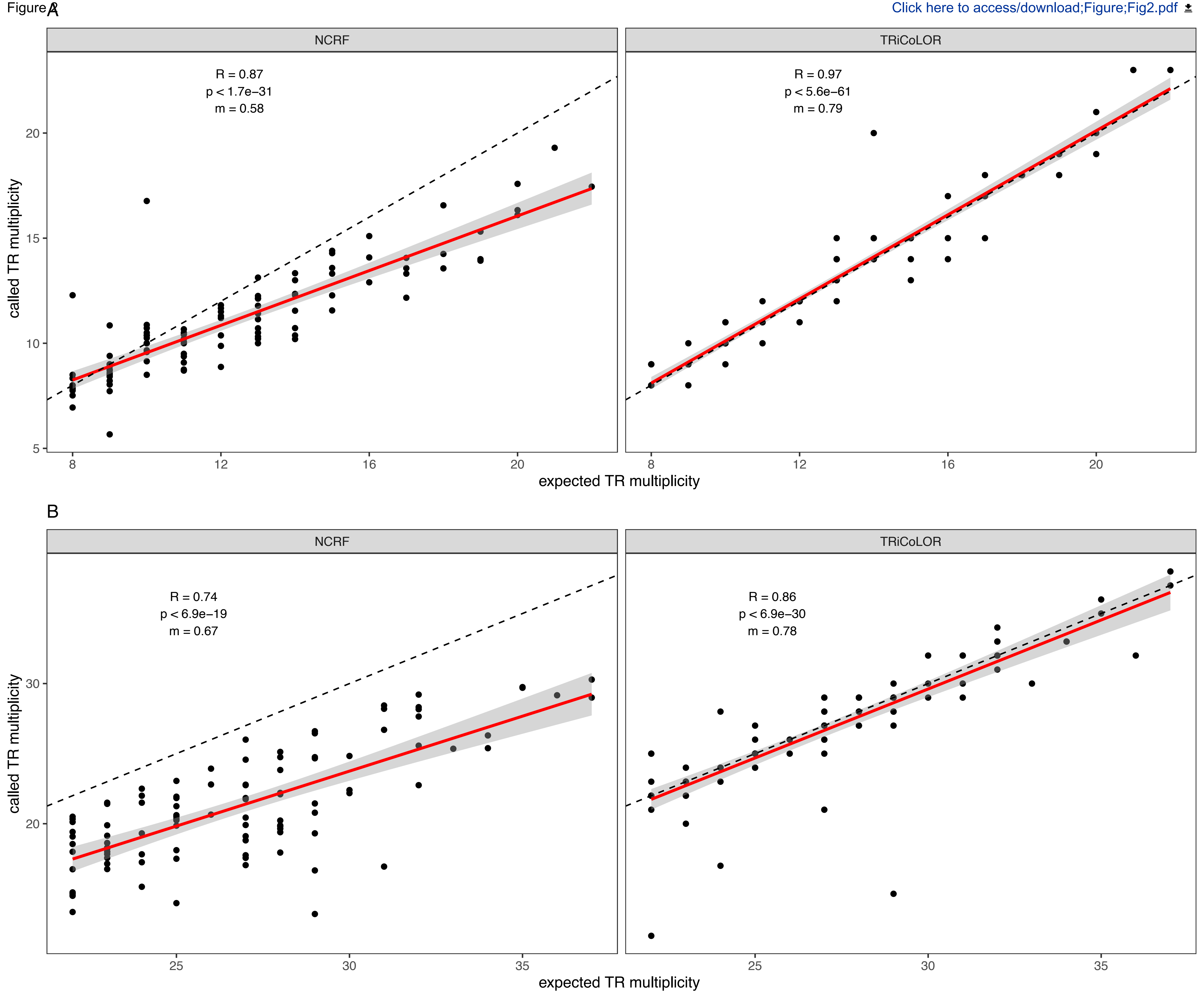

Click here to access/download
Supplementary Material
TRiCoLOR_supplementary_information.pdf

Personal Cover
Click here to access/download;Personal Cover;TRiCoLOR_cover.pdf

Date: August 7, 2020

Manuscript: GIGA-D-20-00168

Dear Editor,

please find enclosed the revised version of our manuscript "TRiCoLOR: tandem repeat profiling using whole-genome long-read sequencing data" for your kind consideration as a *GigaScience* Technical Note.
We appreciate the interest that the Editor and the Reviewers have taken in our manuscript and the largely positive feedback as well as the constructive criticisms they have given. We have addressed all comments of the Reviewers in our point-by-point response.
One comment raised by Reviewers was to improve the guidance regarding the processing steps required before using TRiCoLOR. We therefore decided to extend our supplementary informations with a detailed benchmark of several frameworks for haplotype phasing. The results presented should help readers to identify a proper strategy for preparing their input data prior to TRiCoLOR. We have also included in the revised manuscript a benchmark of the minimap2 presets for mapping consensus sequences to an assembled reference genome.
One Reviewer also requested the application of TRiCoLOR on a non-human organism. Thus, we applied TRiCoLOR to compute a tandem repeat callset for the *Arabidopsis thaliana* KBS-Mac-74 model organism, for which a whole-genome sequencing dataset from Oxford Nanopore Technologies was recently made available. This callset has been added in the revised results.
We hope that you do agree with us that our revisions together with the inclusion of a new tandem repeat callset for a non-human model organism address all comments raised by the reviewers. We envision that TRiCoLOR will facilitate the discovery of tandem repeats in regions which are difficult to be sequenced and interpreted with short read sequencing data sets and it will be an important tool to decipher repeat multiplicity in repeat-mediated clinical disorders.

Thank you for your kind consideration of our revised manuscript in *GigaScience*.

Best regards and on behalf of the authors,

Davide Bolognini
