# Supplementary material for: TRiCoLOR: tandem repeat profiling using whole-genome long-read sequencing data
Source: Gigascience. 2020 Oct 7;9(10):giaa101. doi: 10.1093/gigascience/giaa101 (PMC7539535; doi:10.1093/gigascience/giaa101)
Supplement: giaa101_GIGA-D-20-00168_Revision_3 [file giaa101_giga-d-20-00168_revision_3.pdf]

GigaScience

TRiCoLOR: tandem repeat profiling using whole-genome long-read sequencing data

--Manuscript Draft--

**Manuscript Number:**	GIGA-D-20-00168R3
**Full Title:**	TRiCoLOR: tandem repeat profiling using whole-genome long-read sequencing data
**Article Type:**	Technical Note
**Funding Information:**	GraphGenomes (031L0184C) — Dr. Jan O. Korbel<br>Associazione Italiana per la Ricerca sul Cancro (20307) — Dr. Alberto Magi
**Abstract:**	Background: Tandem repeat sequences are widespread in the human genome and their expansions cause multiple repeat-mediated disorders. Genome-wide discovery approaches are needed to fully understand their roles in health and disease but resolving tandem repeat variation accurately remains a very challenging task. While traditional mapping-based approaches using short-read data have severe limitations in the size and type of tandem repeats they can resolve, recent third-generation sequencing technologies exhibit substantially higher sequencing error rates which complicates repeat resolution.<br>Results: We developed TRiCoLOR, a freely-available tool for tandem repeat profiling using error-prone long reads from third-generation sequencing technologies. The method can identify repetitive regions in sequencing data without a prior knowledge of their motifs or locations and resolve repeats multiplicity and period size in a haplotype-specific manner. The tool includes methods to interactively visualize the identified repeats and to trace their Mendelian consistency in pedigrees.<br>Conclusions: TRiCoLOR demonstrates excellent performance and improved sensitivity and specificity compared to alternative tools on synthetic data. For real human whole-genome sequencing data, TRiCoLOR achieves high validation rates suggesting its suitability to identify tandem repeat variation in personal genomes.
**Corresponding Author:**	Davide Bolognini<br>University of Florence<br>Florence, Florence, Tuscany ITALY
**Corresponding Author Secondary Information:**	
**Corresponding Author's Institution:**	University of Florence
**Corresponding Author's Secondary Institution:**	
**First Author:**	Davide Bolognini
**First Author Secondary Information:**	
**Order of Authors:**	Davide Bolognini<br>Alberto Magi<br>Vladimir Benes<br>Jan O. Korbel<br>Tobias Rausch
**Order of Authors Secondary Information:**	
**Response to Reviewers:**	Dear Editor,<br><br>thanks for Your editorial work with our manuscript. We have addressed the minor edits required to meet GigaScience's formatting and reproducibility guidelines, as from Your decision letter. Thank You for publishing our revised manuscript in GigaScience.
**Additional Information:**	

Powered by Editorial Manager® and ProduXion Manager® from Aries Systems Corporation

Question	Response
Are you submitting this manuscript to a special series or article collection?	No
**Experimental design and statistics**<br><br>Full details of the experimental design and statistical methods used should be given in the Methods section, as detailed in our Minimum Standards Reporting Checklist. Information essential to interpreting the data presented should be made available in the figure legends.<br><br>Have you included all the information requested in your manuscript?	Yes
**Resources**<br><br>A description of all resources used, including antibodies, cell lines, animals and software tools, with enough information to allow them to be uniquely identified, should be included in the Methods section. Authors are strongly encouraged to cite Research Resource Identifiers (RRIDs) for antibodies, model organisms and tools, where possible.<br><br>Have you included the information requested as detailed in our Minimum Standards Reporting Checklist?	Yes
**Availability of data and materials**<br><br>All datasets and code on which the conclusions of the paper rely must be either included in your submission or deposited in publicly available repositories (where available and ethically appropriate), referencing such data using a unique identifier in the references and in the "Availability of Data and Materials" section of your manuscript.	Yes

Have you have met the above requirement as detailed in our Minimum Standards Reporting Checklist?	

LaTeX - Main Document (TeX file)

Click here to access/download;LaTeX - Main Document (TeX file);main.tex

Placeholder for OUP logo oup.pdf

Placeholder for journal logo gigascience-logo.pdf

GigaScience, 2020, 1–7

doi: xx.xxxx/xxxx
Manuscript in Preparation
Technical Note

TECHNICAL NOTE

TRiCoLOR: tandem repeat profiling using whole-genome long-read sequencing data

Davide Bolognini[1,3,*], Alberto Magi[2], Vladimir Benes[3], Jan O. Korbel[4] and Tobias Rausch[3,4]

[1]Department of Experimental and Clinical Medicine, University of Florence, Florence, 50134, Italy and [2]Department of Information Engineering, University of Florence, Florence, 50134, Italy and [3]European Molecular Biology Laboratory (EMBL), GeneCore, Heidelberg, 69117, Germany and [4]European Molecular Biology Laboratory (EMBL), Genome Biology Unit, Heidelberg, 69117, Germany

[*]davidebolognini7@gmail.com

Abstract

Background: Tandem repeat sequences are widespread in the human genome and their expansions cause multiple repeat-mediated disorders. Genome-wide discovery approaches are needed to fully understand their roles in health and disease but resolving tandem repeat variation accurately remains a very challenging task. While traditional mapping-based approaches using short-read data have severe limitations in the size and type of tandem repeats they can resolve, recent third-generation sequencing technologies exhibit substantially higher sequencing error rates which complicates repeat resolution.
Results: We developed TRiCoLOR, a freely-available tool for tandem repeat profiling using error-prone long reads from third-generation sequencing technologies. The method can identify repetitive regions in sequencing data without a prior knowledge of their motifs or locations and resolve repeats multiplicity and period size in a haplotype-specific manner. The tool includes methods to interactively visualize the identified repeats and to trace their Mendelian consistency in pedigrees.
Conclusions. TRiCoLOR demonstrates excellent performance and improved sensitivity and specificity compared to alternative tools on synthetic data. For real human whole-genome sequencing data, TRiCoLOR achieves high validation rates suggesting its suitability to identify tandem repeat variation in personal genomes.

Key words: long-read sequencing; tandem repeat variation; bioinformatics software

Background

Almost half of the human genome is estimated to be covered by repetitive sequences [1]. Among these, tandem repeats (TR) have been found to be involved in a range of functions such as DNA repair, chromatin organization, telomere maintenance, and regulation of gene expression [2]. Most importantly, more than 40 diseases, primarily neurological, are known to be related to TR expansions [3]. Despite their clinical importance, accurately resolving TRs remains challenging in sequencing data sets mainly because of insufficient read lengths failing to encompass entire expanded repeats or technological limitations, such as high sequencing error-rates.
Prior methods for TR profiling in short-read sequencing data sets can be broadly classified as reference-based [4, 5] or *de novo* [6, 7] approaches. While the former investigates only reads spanning known TRs, the latter can identify TRs regardless of whether their repeat motif is annotated or not in the reference. Short read methods are often inadequate to accurately resolve expanded TRs if the total repeat length is greater than the read length.
Long reads from third-generation sequencing technologies, namely Oxford Nanopore Technologies (ONT) and Pacific Biosciences (PB), have proved already invaluable for the discovery

Compiled on: September 3, 2020.
Draft manuscript prepared by the author.

of large structural variants [8] and are obvious candidates for broadening the scope of detectable TRs. However, long reads exhibit high sequencing error rates which make it difficult to accurately decipher TRs, especially in low complexity regions. Few TR detection methods for long-read sequencing data have been developed so far. Examples include PacmonSTR [9], NCRF [10], TideHunter [11], NanoSatellite [12] and Tandem-genotypes [13]. However, these tools have some limitations, either because they are technology-specific (PacmonSTR and NanoSatellite), because they are not intended to be used genome-wide (NCRF, TideHunter and NanoSatellite) or because they require substantial preprocessing steps preventing their large-scale use (Tandem-genotypes). Some tools also lack genotyping capabilities (NCRF and TideHunter) and none of the aforementioned methods is capable to profile TRs *de novo* in regions that have previously not been annotated as harboring a TR.
TRiCoLOR addresses these shortcomings of existing, alignment-based tools by allowing users to rapidly identify and genotype TRs from haplotype-resolved long-read alignments. Once low-entropy repetitive regions have been identified in sequenced long reads, TRiCoLOR exploits partial order alignment (POA) [14] to compute haplotype-specific low-error consensus sequences [15] that are further processed by means of a fast regular expression (RegEx)-based approximate string matching algorithm to resolve repeat motif and multiplicity of the discovered TRs. Detected TRs can be interactively visualized within their haplotype-specific sequence context for manual exploration of expanded or contracted repeats. For trio sequencing studies, TRiCoLOR additionally allows to trace Mendelian inheritance patterns across TR genotypes.

Methods

TRiCoLOR (Tandem Repeats Caller for LOng Reads) requires haplotype-resolved long-read alignments as input (Supplementary Note S1). It then runs a series of modules to identify and genotype TRs as described in detail below. A manual containing an in-depth explanation of how to install TRiCoLOR and run its various modules is available at https://davidebolo1993.github.io/tricolordoc, including use case examples.

Identifying repetitive regions *de novo*

TRiCoLOR can identify repetitive regions in haplotype-resolved BAM files *de novo*. This is achieved using the SENSoR (Shannon ENtropy ScanneR) module, which uses the Shannon entropy of DNA sequences to identify candidate repetitive segments in genomic sequences [16]. TRiCoLOR SENSoR scans in parallel the haplotype-specific BAM files and computes, for each sequencing read, its Shannon entropy content in non-overlapping, sliding windows of a pre-trained size (20 bps, by default). Genomic coordinates of windows in which multiple reads ($\geq$5, by default) support an entropy drop ($\leq$ 1.23, by default) are stored and those nearby are merged (those falling within 100 bps intervals, by default). The default entropy treshold of 1.23 efficiently discriminates between repetitive and non-repetitive DNA sequences using syntethic ONT and PB reads as shown in Supplementary Figure S1 (see Supplementary Note S2). All candidate repetitive regions identified with this approach are eventually outputted in BED format. This pre-filtering of repetitive regions is fairly fast even in deep-coverage whole-genome data (see also *Findings*) and drastically reduces the computational time required for the subsequent TR profiling.

Profiling repetitive regions

TRiCoLOR can profile TRs in haplotype-resolved BAM files through the REFER (REpeats FindER) module. The input of REFER is a BED file generated by TRiCoLOR SENSoR. Alternatively, the BED file can be provided by the user based on prior knowledge of clinically relevant TRs, for instance.
For each region in the BED file, REFER first fetches from the haplotype-specific BAM files the sequencing reads spanning the selected region and trims them, so that the length of each read is approximately the size of the region. Let $R = [S, E]$ be a region from the BED file, ranging from a start coordinate S to an end coordinate E for a given chromosome. Each sequencing read entirely spanning R is fetched and trimmed so that the actual sequence REFER stores is that included between S and E, which significantly improves the runtime of the subsequent POA algorithm to generate a consensus sequence.
Once the sequencing reads of interest have been collected and trimmed, TRiCoLOR uses SPOA [17], a single-instruction multiple-data accelerated version of the robust POA framework, to compute highly accurate consensus sequences with an approximate error reduction of ~77% and ~88% for ONT and PB, respectively (Supplementary Note S3 and Supplementary Figure S2).
With the haplotype-specific consensus sequences at hand, REFER aligns these to the reference genome using minimap2 [18], which compared favorably to alternative aligners on syntethic data, both in terms of speed and mapping accuracy (Supplementary Note S4 and Supplementary Figure S3). The reference-aligned low-error consensus sequences are then screened by a RegEx-based approximate string matching algorithm, which has three processing steps: (1) identifying motifs (motifs of length $\leq$ 6, by default) that are perfectly repeated a minimum number of times (5, by default); (2) looking for approximate repetitions of the identified motifs to account for remaining consensus errors, that is imperfect repeated motifs up to a user-defined edit distance ($\leq$ 1, by default); (3) in case of multiple overlapping approximate repetitions, resolving these competing tandem repeat predictions using an N-gram model that favors the most frequently occurring perfect repeat motif.
Together with the haplotype-specific consensus sequences, the corresponding reference is screened in a similar manner, with few differences being noteworthies: (1) the algorithm assumes the reference does not contain errors and does not look for approximate repetitions of the motifs identified; (2) among overlapping repetitions, the longest repeat is taken.
TRs (those $\geq$ 50 bps, by default) varying between the haplotypes or the reference are eventually stored in BCF-compliant format. TRiCoLOR REFER also stores in the output folder several BED files describing the TRs identified (both for the reference and each haplotype) and haplotype-specific BAM files containing the aligned consensus sequences.

Visualizing identified repeats

The TRs profiled using TRiCoLOR REFER can be interactively visualized through the ApP (Alignment Plotter) module. This module takes as inputs the BED and the BAM files generated by TRiCoLOR REFER together with an additional BED file describing one or more regions to plot.
TRiCoLOR ApP produces a static HTML file illustrating the alignment between the reference and the individual's haplotypes at single base resolution, highlighting the TRs detected. (Supplementary Note S5 and Supplementary Figures S4A-S4C).

Tracing Mendelian inheritance patterns of identified repeats

In pedigree studies, assigned genotypes can be either Mendelian consistent or inconsistent. TRiCoLOR enables genotype consistency checks for TRs identified in the index child when haplotype-resolved long-read alignments for both parents are available. This is achieved through the SAGE (SAmple GEnotyper) module with special emphasis on the common situation that parents have been sequenced at low depth.
Using the same aforementioned TRiCoLOR REFER approach, SAGE computes haplotype-specific consensus alignments for each child TR in each parent. Next the module checks whether the parental TRs are more similar (*i.e.*, have a lower edit distance) to the reference or to the TR identified in the child and assigns them the most likely genotype. Knowing the genotype of both parents, the module eventually flags each TR as Mendelian consistent or inconsistent with the −*mendel* parameter enabled. The output of TRiCoLOR SAGE is a multi-sample BCF file that contains the genotypes for the index child and both parents.

Findings

We benchmarked TRiCoLOR using both synthetic data generated with VISOR [19] and real, publicly available data from the Human Genome Structural Variation Consortium (HGSVC) [8].

Benchmarking TRiCoLOR on synthetic data

We used the TR simulator VISOR to generate synthetic ONT and PB alignments containing TR contractions and expansions. First, we simulated haplotype-resolved ONT and PB BAM files (the average length of simulated reads was set to 8000 bps based on statistics derived from recent ONT sequencing runs [20]; the substitution:insertion:deletion ratio was set to ∼45:25:30 for the synthetic ONT reads and to ∼15:50:35 for the synthetic PB reads, in accordance with findings described in Supplementary Note S3) exhibiting variable error rates (accuracy of reads ∼0.85, ∼0.90 and ∼0.95) and depth of coverage (haplotype-specific depth of coverage 5X-10X and 10X-20X), with each BAM file harboring a heterozygous contraction or expansion of a known, randomly chosen, TR. At this stage, we simulated small TR contractions/expansions (contractions/expansions of 7 motifs on average) in order to evaluate the capability of our method to spot even minor changes in the TR multiplicity of the 2 haplotypes. For each group, we simulated 200 haplotype-resolved BAM files. Then, we evaluated the performances of TRiCoLOR in terms of precision (P), recall (R), and F1 score (F1) (Supplementary Note S6). In particular, P, R and F1 values were calculated allowing no discrepancies, 1 discrepancy or 2 discrepancies between the number of repeated motifs in the ground truth and the number of repeated motifs predicted by TRiCoLOR. Figure 1 shows these findings for synthetic TR contractions (panel A) and expansions (panel B). TRiCoLOR demonstrated high P and R in all the simulated groups: our method always achieved an F1 close to 1 when allowing a single-motif discrepancy between simulated and predicted TRs and hit P ∼1 and R ∼1 when allowing up to 2 motif discrepancies. For both contractions and expansions the F1 depends on the coverage and input read accuracy as expected. In all the simulated TR contractions and expansions, TRiCoLOR was also able to properly identify the correct repeated motif, few times shifted (*e.g.*, a repeated TG instead of a repeated GT). Supplementary Figure S5 illustrates these findings for the same simulated groups of Figure 1, averaged over the different accuracy levels.

Furthermore, as a proof of concept, we compared TRiCoLOR to a TR caller for long reads recently published, namely NCRF. Using the same approach described above, we simulated 100 ONT and 100 PB BAM files (accuracy of reads ∼0.90, depth of coverage for each haplotype 5X-10X), each harboring a small TR contraction/expansion and we run both TRiCoLOR and NCRF on these data. As NCRF cannot deal with BAM input, we slightly modified TRiCoLOR to store in FASTA format the sequences used for the consensus computation step, which could be processed through NCRF (Supplementary Note S7). Figure 2 shows the correlation results between the number of repeated motifs in the ground truth and the number of repeated motifs predicted by TRiCoLOR and NCRF for the simulated TR contractions (panel A) and expansions (panel B). For both TR contractions and expansions, TRiCoLOR got excellent R scores (R = 0.97 for contractions and R = 0.86 for expansions), outperforming NCRF (R = 0.87 for contractions and R = 0.74 for expansions). We next evaluated exceptionally long TR expansions because these have been implicated in several neurological disorders. For instance, the common Fragile-X Syndrome is related to a CGG-repeat usually consisting of ≤55 repeated motifs that expands to ≥ 200 repeated motifs. Following the simulation schema described above, we generated 100 ONT and 100 PB synthetic BAM files harboring TRs expanded by 200 motifs and we run both TRiCoLOR and NCRF on these data. Supplementary Figure S6 shows the correlation results between the number of repeated motifs in the ground truth and the number of repeated motifs predicted by TRiCoLOR and NCRF for the simulated long TR expansions. As above, TRiCoLOR achieved the best R score (R = 0.73), outperforming NCRF (R = 0.53).

Benchmarking TRiCoLOR on real data

We applied TRiCoLOR to call TRs *de novo* on publicly available ONT and PB human whole-genome sequencing data from the HGSVC project. In particular, we used the ONT sequencing data for HG00514 (Han Chinese), HG00733 (Puerto Rican) and NA19240 (Yoruban Nigerian) and the PB sequencing data for HG00731 (Puerto Rican, father), HG00732 (Puerto Rican, mother) and HG00733 (son).
We aligned the ONT FASTQ files to the human GRCh38 reference genome using minimap2 and we merged the chromosome-specific PB alignments using samtools [21]. We then split the ONT and PB alignments by haplotype with Alfred [22] using phased single-nucleotide variants from the HGSVC project.
We calculated the coverage of the initial and the haplotype-resolved BAM files using mosdepth [23]. For all the ONT samples, we identified an initial ∼20X coverage (HG00733 ∼21X, HG00514 ∼23X and NA19240 ∼24X), slightly reduced after splitting by haplotype due to some unassigned reads (HG00733 ∼8X, HG00514 ∼9X and NA19240 ∼10X for each haplotype). For the PB samples, we identified a ∼42X coverage for HG00733 and ∼21X coverage for HG00731 and HG00732, reduced after splitting the data by haplotype (HG00733 ∼14X, HG00731 and HG00732 ∼8X for each haplotype).
We then run TRiCoLOR SENSoR using the default parameter settings on the HG00733 (ONT and PB), HG00514 and NA19240 individuals. Using an Ubuntu 16.04.6 LTS desktop with Intel®Xeon®processors X5460 (clock rate 2.93 GHz), the module took ∼4 hours to scan the ONT samples and ∼8 hours to scan the PB sample, which reflects the higher coverage available for PB. For the HG00733, HG00514 and NA19240 ONT individuals the module identified ∼160000, ∼190000 and ∼260000 low-entropy regions (average length of the regions ∼900 bps), which were reduced to ∼70000, ∼100000 and ∼160000 respectively after filtering for regions with average coverage > 8.

For the HG00733 PB individual the module identified ~380000 low-entropy regions (average length of the regions ~850 bps), which were reduced to ~150000, after filtering for regions with average coverage > 10. For HG00733, ~97% of the low-entropy regions originally identified in the ONT individual overlapped those in the PB one; due to the different coverage distributions, this percentage was reduced to ~31% after filtering.

We run TRiCoLOR REFER on the samples processed by TRiCoLOR SENSoR using the default parameter settings. With 7 processors on our Ubuntu desktop, the module took ~10-12 hours to profile TRs on the ONT individuals and ~14 hours to profile TRs on the PB individual.

We calculated the number of TRs properly called by TRiCoLOR using an alignment-free validation approach. Current benchmarks for TR calling in human genomes are mainly based on short-read sequencing and are biased towards regions of the genome that are easy-to-call with such a technology [24]. It has been shown that it is often impossible to accurately map or even assemble short reads originating from repetitive regions [25], and as a consequence of this, some TRs are missing from the available TR callsets. Following the idea from Dolle *et al.* [26] we first built full-text searchable FM indexes [27] both for the GRCh38 human reference FASTA and the high-quality Illumina FASTQ files of the HG00733, HG00514 and NA19240 individuals. Then, for each individuals' variant identified by TRiCoLOR REFER, the validation algorithm: (1) checks if the variant sequence appears one or more times in the reference FM index: if so, using the consensus BAM files stored by TRiCoLOR REFER, the variant sequence is extended by 1 bp to the left and 1 bp to the right and step 1 is repeated;if not, the algorithm proceeds to the next step; (2) checks if the variant appears one or more times in the corresponding Illumina FM index: if so, the variant is considered a valid call; if not, the variant is considered an invalid call. Taking into account possible errors both in the consensus sequences generated by TRiCoLOR and in the Illumina sequences, we counted as valid calls also variants that are found in the Illumina FM indexes with up to 2 bp discrepancies (*i.e.* their edit distance is ≤ 2). Limited by the length of the available Illumina sequences, using this approach we could not validate variant TRs longer than 124 bps. Overall, we got high validation ratios (ratios between the valid calls and the number of calls that could be assessed using short reads): ~82% for HG00733 (ONT and PB), ~85% for HG00514 and ~86% for NA19240 (Supplementary Figure S7).

We eventually run TRiCoLOR SAGE on the Puerto Rican PB trio HG00731, HG00732 and HG00733, with the default parameter settings and the −*mendel* parameter enabled to check the Mendelian consistency of the TRs identified in HG00733. With 7 processors on our Ubuntu desktop, the module took ~2 hours to complete the analysis. Filtering for variants differing from the reference for at least 10 bps and for multi-allelic variants differing from each other by the same distance, we identified ~80% of Mendelian consistent TRs, which is low compared to trio-based single-nucleotide variant and InDel Mendelian consistency rates, but above reported genotype agreement rates for structural variants in repetitive regions [28].

Among the Mendelian consistent TRs called by TRiCoLOR on the HG00733 PB individual, we identified 32 long TRs (≥ 150 bps) that were absent in the HGSVC ground truth for the same individual. In order to identify the cause of these apparent discrepancies, we aligned the HG00733 phased contigs from HGSVC to the GRCh38 human reference genome with minimap2, using the assembly-to-reference alignment mode and the parameters suggested by QUAST-LG [29] and we manually inspected the discordant TRs in the aligned contigs using IGV [30]. As shown in Table 1, out of 58 non-reference TR alleles identified by TRiCoLOR, we could visually confirm 42 (~75%) of them in the HGSVC assembly, which means that both TRiCoLOR and the HGSVC predicted the same variant type (deletion or insertion) and the predicted variant size is roughly similar (*i.e.* the difference does not exceed 50 bps). However, for the other 16 variants (~25%), the HGSVC assembly either did not contain the allele predicted by TRiCoLOR or did not cover the investigated region, which suggests that mapping-based and assembly-based approaches can be complementary for TR detection using long reads.

Table 1. Comparison between TRiCoLOR's mapping-based and HGSVC's assembly-based approaches for Mendelian consistent long TRs identified by TRiCoLOR on the HG0733 PB individual.

chromosome	start	end	HGSVC assembly*	TRiCoLOR call*
chr1	23703657	23703893	DEL;INS	DEL;INS
chr1	223672571	223672681	INS;INS	INS;INS
chr10	69539376	69539572	INS;INS	INS;INS
chr11	79190887	79191145	**REF**;**REF**	**DEL**;**INS**
chr11	128436913	128437081	INS;INS	INS;INS
chr14	84276747	84276903	**REF**;DEL	**INS**;DEL
chr15	70364402	70364587	INS;**NA**	INS;**INS**
chr16	3529535	3529854	REF;**DEL**	LC;**INS**
chr17	27525992	27526118	INS;INS	INS;INS
chr18	44544809	44545037	INS;INS	INS;INS
chr18	59081301	59081379	INS;**INS**	INS;**INS**
chr18	71198388	71198450	REF;**NA**	REF;**INS**
chr2	160426201	160426342	INS;INS	INS;INS
chr2	211860947	211861156	DEL;**NA**	DEL;**INS**
chr21	35063465	35063588	INS;INS	INS;INS
chr22	46174187	46174274	REF;INS	REF;INS
chr3	13856835	13857013	DEL;INS	DEL;INS
chr4	13807826	13807982	REF;**REF**	REF;**INS**
chr4	18837113	18837320	INS;DEL	INS;DEL
chr4	81637241	81637408	DEL;DEL	DEL;DEL
chr5	54513584	54513735	**REF**;INS	**INS**;INS
chr6	25450910	25450975	REF;**INS**	REF;**INS**
chr6	55543085	55543393	INS;INS	INS;INS
chr6	106945844	106946002	DEL;**DEL**	DEL;**INS**
chr7	38610247	38610412	**NA**;DEL	**INS**;DEL
chr7	71847696	71847865	INS;INS	INS;INS
chr7	109663557	109663744	INS;DEL	INS;DEL
chr7	131933466	131933651	INS;INS	INS;INS
chr9	82850174	82850347	DEL;DEL	DEL;DEL
chr9	91622218	91622365	**NA**;NA	**INS**;REF
chr9	91634814	91634973	**NA**;**NA**	**DEL**;**INS**
chr9	116632126	116632280	INS;INS	INS;INS

* DEL indicates a deletion; INS indicates an insertion; REF indicates a reference allele; NA indicates that the region is not covered by the assembly or mis-assembled; LC indicates that TRiCoLOR could not generate a consensus sequence for the allele due to the low coverage in the region. The 2 alleles are separated by a semicolon.

Discussion

TRiCoLOR is a comprehensive TR caller for long reads that supports the *de novo* identification of TRs in whole-genome sequencing data. TRiCoLOR profiles TRs through an efficient POA algorithm combined with a RegEx-based string matching search, facilitating a robust and accurate discovery of the full spectrum of expanded and contracted TRs in personal genomes.

In comparison to previous tools, TRiCoLOR works with ONT and PB data seamlessly. TRiCoLOR also identifies TRs *de novo* and does not require *a priori* knowledge of annotated TR regions. The unique combination of features for genome-wide, *de novo* discovery and genotyping of TRs in ONT and PB data is to the best of our knowledge unmet by any other TR caller for long-read data. Besides the detection of TRs, TRiCoLOR visualizes TRs in their haplotype context and it can infer parental genotypes using low-coverage parental sequencing data.

TRiCoLOR has been designed for diploid organisms (Supplementary Note S8) and future work includes extending its fea-

ture set to polyploid species and haploid chromosomes (human Y chromosome). As a mapping-based approach, TRiCoLOR cannot identify repeats in unassembled regions of the genome (*e.g.*, human centromeres and telomeres). Furthermore, the entropy threshold and window size for the *de novo* identification of repetitive stretches that we empirically estimated is well-suited for short repeated motifs (2-3 bps) but may need adjustments for long motifs of higher nucleotide complexity. Lastly, by default TRiCoLOR profiles TRs with motif lengths $\leq$ 6 bps (also known as micro-satellites), excluding those with motif lengths $\geq$7 bps (also known as mini-satellites), which are less abundant in diploid organisms [31]. The RegEx algorithm can be also tuned to profile mini-satellites (*i.e.*, by extending the –*size* parameter) but TRiCoLOR has been extensively applied so far only to micro-satellites.
Given these limitations, future work will focus on extending TRiCoLOR to other ploidies, broadening the size spectrum of detectable repeat motif lengths and taking advantage of improved sequencing read accuracy (*e.g.*, high-fidelity long reads from PB). The latter directly improves the RegEx-based identification of repeats employed by TRiCoLOR and we thus believe TRiCoLOR is well-suited to characterize the TR landscape in present and future long-read data sets, making it an instrumental tool to robustly decipher the multiplicity of TRs in repeat-mediated clinical disorders.

Availability of source code and requirements

- Project name: TRiCoLOR (SciCrunch RRID: SCR_018801; bio.tools ID: tricolor)
- Project home page: https://github.com/davidebolo1993/TRiCoLOR. A dockerized version of TRiCoLOR is available at https://hub.docker.com/r/davidebolo1993/tricolor. Online documentation is available at https://davidebolo1993.github.io/tricolordoc.
- Operating system: Unix
- Programming languages: Python, Bash, C++
- Other requirements: Python 3.6 or higher, GCC 4.8 or higher and CMake 3.2 or higher.
- License: GNU Lesser General Public License 3.0

Availability of supporting data and materials

HGSVC whole-genome long-read sequencing data are available on the HGSVC website (https://www.internationalgenome.org/human-genome-structural-variation-consortium). Specifically:

- ONT FASTQ files: http://ftp.ebi.ac.uk/1000g/ftp/data_collections/hgsv_sv_discovery/working/20181210_ONT_rebasecalled
- PB alignments: http://ftp.ebi.ac.uk/1000g/ftp/data_collections/hgsv_sv_discovery/working/20180102_pacbio_blasr_reheader
- Phased single-nucleotide variants: http://ftp.ebi.ac.uk/1000g/ftp/data_collections/hgsv_sv_discovery/working/20170323_Strand-seq_phased_FB%2BGATK_VCFs
- Illumina FASTQ files: http://ftp.ebi.ac.uk/1000g/ftp/data_collections/hgsv_sv_discovery/illumina_wgs.sequence.index
- HG00733 phased contigs: http://ftp.ebi.ac.uk/1000g/ftp/data_collections/hgsv_sv_discovery/working/20180227_PhasedSVGenomes
- HG00733 ground truth of structural variant calls: http://ftp.ebi.ac.uk/1000g/ftp/data_collections/hgsv_sv_discovery/working/20180627_PanTechnologyIntegrationSet/HG00733.merged_nonredundant.vcf

The GRCh38 human reference genome used for alignments is available at http://ftp.ebi.ac.uk/1000g/ftp/technical/reference/GRCh38_reference_genome/GRCh38_full_analysis_set_plus_decoy_hla.fa. The corresponding annotated TRs can be accessed through the UCSC Table Browser tool (http://genome.ucsc.edu).
A whole-genome ONT FASTQ file of the *Arabisopsis thaliana* KBS-Mac-74 is available at ftp://ftp.sra.ebi.ac.uk/vol1/fastq/ERR217/003/ERR2173373/ERR2173373.fastq.gz. The TAIR10 reference genome for *Arabidopsis thaliana* can be downloaded through the Arabidopsis Information Resource database (https://www.arabidopsis.org/index.jsp) Several scripts used to perform the analyses described in this paper and the TR calls generated by TRiCoLOR for the HGSVC individuals and the *Arabisopsis thaliana* KBS-Mac-74 are available through the GitHub code repository of TRiCoLOR (https://github.com/davidebolo1993/TRiCoLOR). More in detail:

- the https://github.com/davidebolo1993/TRiCoLOR/tree/master/paper/data folder contains the BED file with annotated TRs from the GRCh38 human reference genome (`GRCh38.TRs.bed`), a bash script that illustrates how to haplotype-resolve a long-read alignment using phased single-nucleotide variants (`prepare.sh`), a python script used for the Shannon entropy simulations (`entropy.py`), a python script used to calculate precision, recall and F1 scores of TRiCoLOR on synthetic data (`pr.py`) and a couple of C++ source code files (`fmindex.cpp` and `validate.cpp`) for validating TRiCoLOR calls on real human data.
- the https://github.com/davidebolo1993/TRiCoLOR/tree/master/paper/samples folder contains TRiCoLOR calls for the HGSVC individuals and the *Arabisopsis thaliana* KBS-Mac-74 in standard BCF format.

A snapshot of the archival code is available in the *GigaScience* GigaDB database[32] .

Declarations

List of abbreviations

TR: tandem repeat; ONT: Oxford Nanopore Technologies; PB: Pacific Biosciences; POA: partial order alignment; RegEx: regular expression; HGSVC: Human Genome Structural Variation Consortium; P: precision; R: recall; F1: F1 score.

Competing Interests

The authors declare that they have no competing interests.

Funding

JOK is supported by GraphGenomes grant 031L0184C. AM is supported by AIRC grant 20307. The funders had no role in study design, data collection and analysis, decision to publish, or preparation of the manuscript.

Author's Contributions

DB and TR designed and benchmarked the software. DB wrote the code. TR supervised the work. DB and TR co-wrote the manuscript draft. AM, VB and JOK contributed to the interpretation of the results, provided critical feedback and helped to write the manuscript. All the authors read and approved the manuscript.

Acknowledgements

The authors thank HGSVC for data access, and EMBL GeneCore and IT for technical support.

References

1. de Koning APJ, Gu W, Castoe TA, Batzer MA, Pollock DD. Repetitive elements may comprise over Two-Thirds of the human genome. PLoS Genetics 2011;.
2. Hannan AJ, Tandem repeats mediating genetic plasticity in health and disease; 2018.
3. Paulson H. Repeat expansion diseases. In: Handbook of Clinical Neurology; 2018.
4. Gymrek M, Golan D, Rosset S, Erlich Y. lobSTR: A short tandem repeat profiler for personal genomes. Genome Research 2012;.
5. Highnam G, Franck C, Martin A, Stephens C, Puthige A, Mittelman D. Accurate human microsatellite genotypes from high-throughput resequencing data using informed error profiles. Nucleic Acids Research 2013;.
6. Doi K, Monjo T, Hoang PH, Yoshimura J, Yurino H, Mitsui J, et al. Rapid detection of expanded short tandem repeats in personal genomics using hybrid sequencing. Bioinformatics 2014;.
7. Velasco A, James BT, Wells VD, Girgis HZ, Elofsson A. Look4TRs: A de novo tool for detecting simple tandem repeats using self-supervised hidden Markov models. Bioinformatics 2020;.
8. Chaisson MJP, Sanders AD, Zhao X, Malhotra A, Porubsky D, Rausch T, et al. Multi-platform discovery of haplotype-resolved structural variation in human genomes. Nature Communications 2019;.
9. Ummat A, Bashir A. Resolving complex tandem repeats with long reads. Bioinformatics 2014;.
10. Harris RS, Cechova M, Makova KD, Birol I. Noise-cancelling repeat finder: Uncovering tandem repeats in error-prone long-read sequencing data. Bioinformatics 2019;.
11. Gao Y, Liu B, Wang Y, Xing Y. TideHunter: Efficient and sensitive tandem repeat detection from noisy long-reads using seed-and-chain. In: Bioinformatics; 2019. .
12. De Roeck A, De Coster W, Bossaerts L, Cacace R, De Pooter T, Van Dongen J, et al. NanoSatellite: Accurate characterization of expanded tandem repeat length and sequence through whole genome long-read sequencing on PromethION. Genome Biology 2019;.
13. Mitsuhashi S, Frith MC, Mizuguchi T, Miyatake S, Toyota T, Adachi H, et al. Tandem-genotypes: robust detection of tandem repeat expansions from long DNA reads. Genome Biology 2019;.
14. Lee C, Grasso C, Sharlow MF. Multiple sequence alignment using partial order graphs. Bioinformatics 2002;.
15. Lee C. Generating consensus sequences from partial order multiple sequence alignment graphs. Bioinformatics 2003;.
16. Tenreiro MacHado JA. Shannon entropy analysis of the genome code. Mathematical Problems in Engineering 2012;.
17. Vaser R, Sović I, Nagarajan N, Šikić M. Fast and accurate de novo genome assembly from long uncorrected reads. Genome Research 2017;.
18. Li H. Minimap2: Pairwise alignment for nucleotide sequences. Bioinformatics 2018;.
19. Bolognini D, Sanders A, Korbel JO, Magi A, Benes V, Rausch T. VISOR: A versatile haplotype-aware structural variant simulator for short-and long-read sequencing. Bioinformatics 2020;.
20. Bolognini D, Bartalucci N, Mingrino A, Vannucchi AM, Magi A. NANOR: A user-friendly R package to analyze and compare nanopore sequencing data. PLoS ONE 2019;.
21. Li H, Handsaker B, Wysoker A, Fennell T, Ruan J, Homer N, et al. The Sequence Alignment/Map format and SAMtools. Bioinformatics 2009;.
22. Rausch T, Hsi-Yang Fritz M, Korbel JO, Benes V. Alfred: Interactive multi-sample BAM alignment statistics, feature counting and feature annotation for long- and short-read sequencing. Bioinformatics 2019;.
23. Pedersen BS, Quinlan AR. Mosdepth: Quick coverage calculation for genomes and exomes. Bioinformatics 2018;.
24. Edge P, Bansal V. Longshot enables accurate variant calling in diploid genomes from single-molecule long read sequencing. Nature Communications 2019;.
25. Mantere T, Kersten S, Hoischen A, Long-read sequencing emerging in medical genetics; 2019.
26. Dolle DD, Liu Z, Cotten M, Simpson JT, Iqbal Z, Durbin R, et al. Using reference-free compressed data structures to analyze sequencing reads from thousands of human genomes. Genome Research 2017;.
27. Ferragina P, Manzini G. Opportunistic data structures with applications. In: Annual Symposium on Foundations of Computer Science - Proceedings; 2000. .
28. Giner-Delgado C, Villatoro S, Lerga-Jaso J, Gayà-Vidal M, Oliva M, Castellano D, et al. Evolutionary and functional impact of common polymorphic inversions in the human genome. Nature Communications 2019;.
29. Mikheenko A, Prjibelski A, Saveliev V, Antipov D, Gurevich A. Versatile genome assembly evaluation with QUAST-LG. In: Bioinformatics; 2018. .
30. Thorvaldsdóttir H, Robinson JT, Mesirov JP. Integrative Genomics Viewer (IGV): High-performance genomics data visualization and exploration. Briefings in Bioinformatics 2013;.
31. Richard GF, Kerrest A, Dujon B. Comparative Genomics and Molecular Dynamics of DNA Repeats in Eukaryotes. Microbiology and Molecular Biology Reviews 2008;.
32. Bolognini D, Magi A, Benes V, , Korbel JO, Rausch T. Supporting data for "TRiCoLOR: tandem repeat profiling using whole-genome long-read sequencing data". GigaScience Database 2020;http://dx.doi.org/10.5524/100790.

Figures

Figure 1. TRiCoLOR's P (x-axis), R (y-axis) and F1 (dashed lines) on syntethic TR contractions (**A**) and expansions (**B**). ONT and PB reads exhibit variable error rates (accuracy ~0.85, red; accuracy ~0.90, blue; accuracy ~0.95, green) and were simulated using variable haplotype-specific depth of coverage. P, R and F1 were calculated allowing no motif discrepancies (circle symbol), 1 motif discrepancy (triangle symbol) or 2 motif discrepancies (rhombus symbol) between TRiCoLOR's predictions and the number of repeated motifs in the ground truth.

Figure 2. Correlation results between the number of repeated motifs in the ground truth (x-axis) and the number of repeated motifs predicted by TRiCoLOR and NCRF (y-axis) for syntethic TR contractions (**A**) and expansions (**B**). Each dot represents the synthetic contraction/expansion of a single TR. R is the Pearson's correlation coefficient, p is the p-value of the linear regression analysis, m is the slope of the regression line and the dashed line is the bisector of the first quadrant angle that marks the perfect correspondence between expected and predicted number of TRs.

Manuscript
Click here to access/download;Manuscript;TRiCoLOR_reference.pdf

OXFORD (GIGA)n SCIENCE

GigaScience, 2020, 1–7

doi: xx.xxxx/xxxx
Manuscript in Preparation
Technical Note

TECHNICAL NOTE

TRiCoLOR: tandem repeat profiling using whole-genome long-read sequencing data

Davide Bolognini[1,3,*], Alberto Magi[2], Vladimir Benes[3], Jan O. Korbel[4] and Tobias Rausch[3,4]

[1]Department of Experimental and Clinical Medicine, University of Florence, Florence, 50134, Italy and [2]Department of Information Engineering, University of Florence, Florence, 50134, Italy and [3]European Molecular Biology Laboratory (EMBL), GeneCore, Heidelberg, 69117, Germany and [4]European Molecular Biology Laboratory (EMBL), Genome Biology Unit, Heidelberg, 69117, Germany

[*]davidebolognini7@gmail.com

Abstract

Background: Tandem repeat sequences are widespread in the human genome and their expansions cause multiple repeat-mediated disorders. Genome-wide discovery approaches are needed to fully understand their roles in health and disease but resolving tandem repeat variation accurately remains a very challenging task. While traditional mapping-based approaches using short-read data have severe limitations in the size and type of tandem repeats they can resolve, recent third-generation sequencing technologies exhibit substantially higher sequencing error rates which complicates repeat resolution.
Results: We developed TRiCoLOR, a freely-available tool for tandem repeat profiling using error-prone long reads from third-generation sequencing technologies. The method can identify repetitive regions in sequencing data without a prior knowledge of their motifs or locations and resolve repeats multiplicity and period size in a haplotype-specific manner. The tool includes methods to interactively visualize the identified repeats and to trace their Mendelian consistency in pedigrees.
Conclusions. TRiCoLOR demonstrates excellent performance and improved sensitivity and specificity compared to alternative tools on synthetic data. For real human whole-genome sequencing data, TRiCoLOR achieves high validation rates suggesting its suitability to identify tandem repeat variation in personal genomes.

Key words: long-read sequencing; tandem repeat variation; bioinformatics software

Background

Almost half of the human genome is estimated to be covered by repetitive sequences [1]. Among these, tandem repeats (TR) have been found to be involved in a range of functions such as DNA repair, chromatin organization, telomere maintenance, and regulation of gene expression [2]. Most importantly, more than 40 diseases, primarily neurological, are known to be related to TR expansions [3]. Despite their clinical importance, accurately resolving TRs remains challenging in sequencing data sets mainly because of insufficient read lengths failing to encompass entire expanded repeats or technological limitations, such as high sequencing error-rates.
Prior methods for TR profiling in short-read sequencing data sets can be broadly classified as reference-based [4, 5] or *de novo* [6, 7] approaches. While the former investigates only reads spanning known TRs, the latter can identify TRs regardless of whether their repeat motif is annotated or not in the reference. Short read methods are often inadequate to accurately resolve expanded TRs if the total repeat length is greater than the read length.
Long reads from third-generation sequencing technologies,

Compiled on: September 3, 2020.
Draft manuscript prepared by the author.

namely Oxford Nanopore Technologies (ONT) and Pacific Biosciences (PB), have proved already invaluable for the discovery of large structural variants [8] and are obvious candidates for broadening the scope of detectable TRs. However, long reads exhibit high sequencing error rates which make it difficult to accurately decipher TRs, especially in low complexity regions.
Few TR detection methods for long-read sequencing data have been developed so far. Examples include PacmonSTR [9], NCRF [10], TideHunter [11], NanoSatellite [12] and Tandem-genotypes [13]. However, these tools have some limitations, either because they are technology-specific (PacmonSTR and NanoSatellite), because they are not intended to be used genome-wide (NCRF, TideHunter and NanoSatellite) or because they require substantial preprocessing steps preventing their large-scale use (Tandem-genotypes). Some tools also lack genotyping capabilities (NCRF and TideHunter) and none of the aforementioned methods is capable to profile TRs *de novo* in regions that have previously not been annotated as harboring a TR.
TRiCoLOR addresses these shortcomings of existing, alignment-based tools by allowing users to rapidly identify and genotype TRs from haplotype-resolved long-read alignments. Once low-entropy repetitive regions have been identified in sequenced long reads, TRiCoLOR exploits partial order alignment (POA) [14] to compute haplotype-specific low-error consensus sequences [15] that are further processed by means of a fast regular expression (RegEx)-based approximate string matching algorithm to resolve repeat motif and multiplicity of the discovered TRs. Detected TRs can be interactively visualized within their haplotype-specific sequence context for manual exploration of expanded or contracted repeats. For trio sequencing studies, TRiCoLOR additionally allows to trace Mendelian inheritance patterns across TR genotypes.

Methods

TRiCoLOR (Tandem Repeats Caller for LOng Reads) requires haplotype-resolved long-read alignments as input (Supplementary Note S1). It then runs a series of modules to identify and genotype TRs as described in detail below. A manual containing an in-depth explanation of how to install TRiCoLOR and run its various modules is available at https://davidebolo1993.github.io/tricolordoc, including use case examples.

Identifying repetitive regions *de novo*

TRiCoLOR can identify repetitive regions in haplotype-resolved BAM files *de novo*. This is achieved using the SENSoR (Shannon ENtropy ScanneR) module, which uses the Shannon entropy of DNA sequences to identify candidate repetitive segments in genomic sequences [16]. TRiCoLOR SENSoR scans in parallel the haplotype-specific BAM files and computes, for each sequencing read, its Shannon entropy content in non-overlapping, sliding windows of a pre-trained size (20 bps, by default). Genomic coordinates of windows in which multiple reads ($\geq$5, by default) support an entropy drop ($\leq$ 1.23, by default) are stored and those nearby are merged (those falling within 100 bps intervals, by default). The default entropy treshold of 1.23 efficiently discriminates between repetitive and non-repetitive DNA sequences using syntethic ONT and PB reads as shown in Supplementary Figure S1 (see Supplementary Note S2). All candidate repetitive regions identified with this approach are eventually outputted in BED format. This pre-filtering of repetitive regions is fairly fast even in deep-coverage whole-genome data (see also *Findings*) and drastically reduces the computational time required for the subsequent TR profiling.

Profiling repetitive regions

TRiCoLOR can profile TRs in haplotype-resolved BAM files through the REFER (REpeats FindER) module. The input of REFER is a BED file generated by TRiCoLOR SENSoR. Alternatively, the BED file can be provided by the user based on prior knowledge of clinically relevant TRs, for instance.
For each region in the BED file, REFER first fetches from the haplotype-specific BAM files the sequencing reads spanning the selected region and trims them, so that the length of each read is approximately the size of the region. Let $R = [S, E]$ be a region from the BED file, ranging from a start coordinate S to an end coordinate E for a given chromosome. Each sequencing read entirely spanning R is fetched and trimmed so that the actual sequence REFER stores is that included between S and E, which significantly improves the runtime of the subsequent POA algorithm to generate a consensus sequence.
Once the sequencing reads of interest have been collected and trimmed, TRiCoLOR uses SPOA [17], a single-instruction multiple-data accelerated version of the robust POA framework, to compute highly accurate consensus sequences with an approximate error reduction of ~77% and ~88% for ONT and PB, respectively (Supplementary Note S3 and Supplementary Figure S2).
With the haplotype-specific consensus sequences at hand, REFER aligns these to the reference genome using minimap2 [18], which compared favorably to alternative aligners on syntethic data, both in terms of speed and mapping accuracy (Supplementary Note S4 and Supplementary Figure S3). The reference-aligned low-error consensus sequences are then screened by a RegEx-based approximate string matching algorithm, which has three processing steps: (1) identifying motifs (motifs of length $\leq$ 6, by default) that are perfectly repeated a minimum number of times (5, by default); (2) looking for approximate repetitions of the identified motifs to account for remaining consensus errors, that is imperfect repeated motifs up to a user-defined edit distance ($\leq$ 1, by default); (3) in case of multiple overlapping approximate repetitions, resolving these competing tandem repeat predictions using an N-gram model that favors the most frequently occurring perfect repeat motif.
Together with the haplotype-specific consensus sequences, the corresponding reference is screened in a similar manner, with few differences being noteworthies: (1) the algorithm assumes the reference does not contain errors and does not look for approximate repetitions of the motifs identified; (2) among overlapping repetitions, the longest repeat is taken.
TRs (those $\geq$ 50 bps, by default) varying between the haplotypes or the reference are eventually stored in BCF-compliant format. TRiCoLOR REFER also stores in the output folder several BED files describing the TRs identified (both for the reference and each haplotype) and haplotype-specific BAM files containing the aligned consensus sequences.

Visualizing identified repeats

The TRs profiled using TRiCoLOR REFER can be interactively visualized through the ApP (Alignment Plotter) module. This module takes as inputs the BED and the BAM files generated by TRiCoLOR REFER together with an additional BED file describing one or more regions to plot.
TRiCoLOR ApP produces a static HTML file illustrating the alignment between the reference and the individual's haplotypes at single base resolution, highlighting the TRs detected. (Supplementary Note S5 and Supplementary Figures S4A-S4C).

Tracing Mendelian inheritance patterns of identified repeats

In pedigree studies, assigned genotypes can be either Mendelian consistent or inconsistent. TRiCoLOR enables genotype consistency checks for TRs identified in the index child when haplotype-resolved long-read alignments for both parents are available. This is achieved through the SAGE (SAmple GEnotyper) module with special emphasis on the common situation that parents have been sequenced at low depth.
Using the same aforementioned TRiCoLOR REFER approach, SAGE computes haplotype-specific consensus alignments for each child TR in each parent. Next the module checks whether the parental TRs are more similar (*i.e.*, have a lower edit distance) to the reference or to the TR identified in the child and assigns them the most likely genotype. Knowing the genotype of both parents, the module eventually flags each TR as Mendelian consistent or inconsistent with the −*mendel* parameter enabled. The output of TRiCoLOR SAGE is a multi-sample BCF file that contains the genotypes for the index child and both parents.

Findings

We benchmarked TRiCoLOR using both synthetic data generated with VISOR [19] and real, publicly available data from the Human Genome Structural Variation Consortium (HGSVC) [8].

Benchmarking TRiCoLOR on synthetic data

We used the TR simulator VISOR to generate synthetic ONT and PB alignments containing TR contractions and expansions. First, we simulated haplotype-resolved ONT and PB BAM files (the average length of simulated reads was set to 8000 bps based on statistics derived from recent ONT sequencing runs [20]; the substitution:insertion:deletion ratio was set to ~45:25:30 for the synthetic ONT reads and to ~15:50:35 for the synthetic PB reads, in accordance with findings described in Supplementary Note S3) exhibiting variable error rates (accuracy of reads ~0.85, ~0.90 and ~0.95) and depth of coverage (haplotype-specific depth of coverage 5X-10X and 10X-20X),with each BAM file harboring a heterozygous contraction or expansion of a known, randomly chosen, TR. At this stage, we simulated small TR contractions/expansions (contractions/expansions of 7 motifs on average) in order to evaluate the capability of our method to spot even minor changes in the TR multiplicity of the 2 haplotypes. For each group, we simulated 200 haplotype-resolved BAM files. Then, we evaluated the performances of TRiCoLOR in terms of precision (P), recall (R), and F1 score (F1) (Supplementary Note S6). In particular, P, R and F1 values were calculated allowing no discrepancies, 1 discrepancy or 2 discrepancies between the number of repeated motifs in the ground truth and the number of repeated motifs predicted by TRiCoLOR. Figure 1 shows these findings for synthetic TR contractions (panel A) and expansions (panel B). TRiCoLOR demonstrated high P and R in all the simulated groups: our method always achieved an F1 close to 1 when allowing a single-motif discrepancy between simulated and predicted TRs and hit P ~1 and R ~1 when allowing up to 2 motif discrepancies. For both contractions and expansions the F1 depends on the coverage and input read accuracy as expected. In all the simulated TR contractions and expansions, TRiCoLOR was also able to properly identify the correct repeated motif, few times shifted (*e.g.*, a repeated TG instead of a repeated GT). Supplementary Figure S5 illustrates these findings for the same simulated groups of Figure 1, averaged over the different accuracy levels.

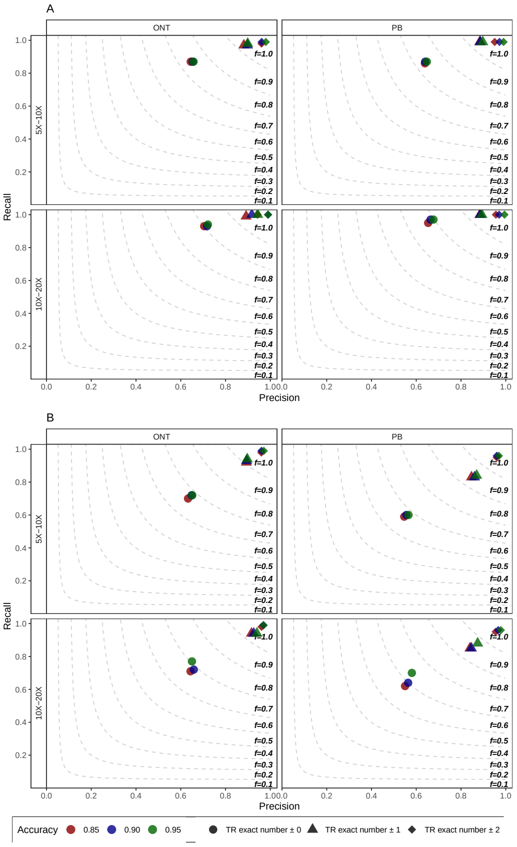

Figure 1. TRiCoLOR's P (x-axis), R (y-axis) and F1 (dashed lines) on syntethic TR contractions (**A**) and expansions (**B**). ONT and PB reads exhibit variable error rates (accuracy ~0.85, red; accuracy ~0.90, blue; accuracy ~0.95, green) and were simulated using variable haplotype-specific depth of coverage. P, R and F1 were calculated allowing no motif discrepancies (circle symbol), 1 motif discrepancy (triangle symbol) or 2 motif discrepancies (rhombus symbol) between TRiCoLOR's predictions and the number of repeated motifs in the ground truth.

Furthermore, as a proof of concept, we compared TRiCoLOR to a TR caller for long reads recently published, namely NCRF. Using the same approach described above, we simulated 100 ONT and 100 PB BAM files (accuracy of reads ~0.90, depth of coverage for each haplotype 5X-10X), each harboring a small TR contraction/expansion and we run both TRiCoLOR and NCRF on these data. As NCRF cannot deal with BAM input, we slightly modified TRiCoLOR to store in FASTA format the sequences used for the consensus computation step, which could be processed through NCRF (Supplementary Note S7). Figure 2 shows the correlation results between the number of repeated motifs in the ground truth and the number of repeated motifs predicted by TRiCoLOR and NCRF for the simulated TR contractions (panel A) and expansions (panel B). For both TR contractions and expansions, TRiCoLOR got excellent R scores (R = 0.97 for contractions and R = 0.86 for expansions), outperforming NCRF (R = 0.87 for contractions and R = 0.74 for expansions). We next evaluated exceptionally long TR expansions because these have been implicated in several neurological disorders. For instance, the common Fragile-X Syndrome is related to a CGG-repeat usually consisting of $\leq$55 repeated motifs that expands to $\geq$ 200 repeated motifs. Following the simulation schema described above, we generated 100 ONT and 100 PB synthetic BAM files harboring TRs expanded by 200 motifs and we run both TRiCoLOR and NCRF on these data. Supplementary

Figure S6 shows the correlation results between the number of repeated motifs in the ground truth and the number of repeated motifs predicted by TRiCoLOR and NCRF for the simulated long TR expansions. As above, TRiCoLOR achieved the best R score (R = 0.73), outperforming NCRF (R = 0.53).

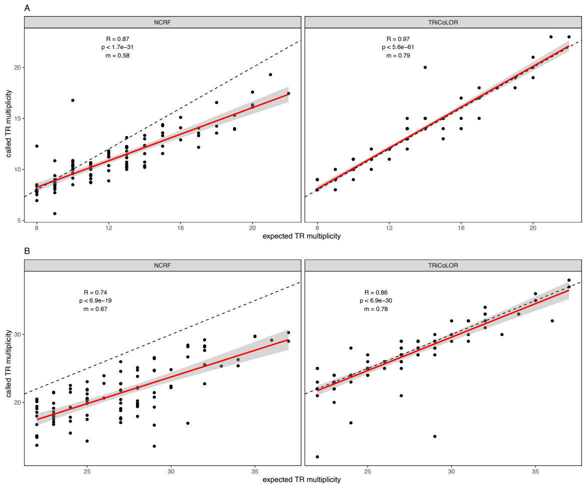

Figure 2. Correlation results between the number of repeated motifs in the ground truth (x-axis) and the number of repeated motifs predicted by TRiCoLOR and NCRF (y-axis) for syntethic TR contractions (**A**) and expansions (**B**). Each dot represents the synthetic contraction/expansion of a single TR. R is the Pearson's correlation coefficient, p is the p-value of the linear regression analysis, m is the slope of the regression line and the dashed line is the bisector of the first quadrant angle that marks the perfect correspondence between expected and predicted number of TRs.

Benchmarking TRiCoLOR on real data

We applied TRiCoLOR to call TRs *de novo* on publicly available ONT and PB human whole-genome sequencing data from the HGSVC project. In particular, we used the ONT sequencing data for HG00514 (Han Chinese), HG00733 (Puerto Rican) and NA19240 (Yoruban Nigerian) and the PB sequencing data for HG00731 (Puerto Rican, father), HG00732 (Puerto Rican, mother) and HG00733 (son).

We aligned the ONT FASTQ files to the human GRCh38 reference genome using minimap2 and we merged the chromosome-specific PB alignments using samtools [21]. We then split the ONT and PB alignments by haplotype with Alfred [22] using phased single-nucleotide variants from the HGSVC project. We calculated the coverage of the initial and the haplotype-resolved BAM files using mosdepth [23]. For all the ONT samples, we identified an initial ~20X coverage (HG00733 ~21X, HG00514 ~23X and NA19240 ~24X), slightly reduced after splitting by haplotype due to some unassigned reads (HG00733 ~8X, HG00514 ~9X and NA19240 ~10X for each haplotype). For the PB samples, we identified a ~42X coverage for HG00733 and ~21X coverage for HG00731 and HG00732, reduced after splitting the data by haplotype (HG00733 ~14X, HG00731 and HG00732 ~8X for each haplotype).

We then run TRiCoLOR SENSoR using the default parameter settings on the HG00733 (ONT and PB), HG00514 and NA19240 individuals. Using an Ubuntu 16.04.6 LTS desktop with Intel®Xeon®processors X5460 (clock rate 2.93 GHz), the module took ~4 hours to scan the ONT samples and ~8 hours to scan the PB sample, which reflects the higher coverage available for PB. For the HG00733, HG00514 and NA19240 ONT individuals the module identified ~160000, ~190000 and ~260000 low-entropy regions (average length of the regions ~900 bps), which were reduced to ~70000, ~100000 and ~160000 respectively after filtering for regions with average coverage > 8. For the HG00733 PB individual the module identified ~380000 low-entropy regions (average length of the regions ~850 bps), which were reduced to ~150000, after filtering for regions with average coverage > 10. For HG00733, ~97% of the low-entropy regions originally identified in the ONT individual overlapped those in the PB one; due to the different coverage distributions, this percentage was reduced to ~31% after filtering.

We run TRiCoLOR REFER on the samples processed by TRiCoLOR SENSoR using the default parameter settings. With 7 processors on our Ubuntu desktop, the module took ~10-12 hours to profile TRs on the ONT individuals and ~14 hours to profile TRs on the PB individual.

We calculated the number of TRs properly called by TRiCoLOR using an alignment-free validation approach. Current benchmarks for TR calling in human genomes are mainly based on short-read sequencing and are biased towards regions of the genome that are easy-to-call with such a technology [24]. It has been shown that it is often impossible to accurately map or even assemble short reads originating from repetitive regions [25], and as a consequence of this, some TRs are missing from the available TR callsets. Following the idea from Dolle *et al.* [26] we first built full-text searchable FM indexes [27] both for the GRCh38 human reference FASTA and the high-quality Illumina FASTQ files of the HG00733, HG00514 and NA19240 individuals. Then, for each individuals' variant identified by TRiCoLOR REFER, the validation algorithm: (1) checks if the variant sequence appears one or more times in the reference FM index: if so, using the consensus BAM files stored by TRiCoLOR REFER, the variant sequence is extended by 1 bp to the left and 1 bp to the right and step 1 is repeated; if not, the algorithm proceeds to the next step; (2) checks if the variant appears one or more times in the corresponding Illumina FM index: if so, the variant is considered a valid call; if not, the variant is considered an invalid call. Taking into account possible errors both in the consensus sequences generated by TRiCoLOR and in the Illumina sequences, we counted as valid calls also variants that are found in the Illumina FM indexes with up to 2 bp discrepancies (*i.e.* their edit distance is $\leq$ 2). Limited by the length of the available Illumina sequences, using this approach we could not validate variant TRs longer than 124 bps. Overall, we got high validation ratios (ratios between the valid calls and the number of calls that could be assessed using short reads): ~82% for HG00733 (ONT and PB), ~85% for HG00514 and ~86% for NA19240 (Supplementary Figure S7).

We eventually run TRiCoLOR SAGE on the Puerto Rican PB trio HG00731, HG00732 and HG00733, with the default parameter settings and the *−mendel* parameter enabled to check the Mendelian consistency of the TRs identified in HG00733. With 7 processors on our Ubuntu desktop, the module took ~2 hours to complete the analysis. Filtering for variants differing from the reference for at least 10 bps and for multi-allelic variants differing from each other by the same distance, we identified ~80% of Mendelian consistent TRs, which is low compared to trio-based single-nucleotide variant and InDel Mendelian consistency rates, but above reported genotype agreement rates for structural variants in repetitive regions [28].

Among the Mendelian consistent TRs called by TRiCoLOR on the HG00733 PB individual, we identified 32 long TRs ($\geq$ 150 bps) that were absent in the HGSVC ground truth for the same individual. In order to identify the cause of these apparent discrepancies, we aligned the HG00733 phased contigs from HGSVC to the GRCh38 human reference genome with minimap2, using the assembly-to-reference alignment mode and the parameters suggested by QUAST-LG [29] and we manually inspected the discordant TRs in the aligned contigs using IGV

Table 1. Comparison between TRiCoLOR's mapping-based and HGSVC's assembly-based approaches for Mendelian consistent long TRs identified by TRiCoLOR on the HG0733 PB individual.

chromosome	start	end	HGSVC assembly*	TRiCoLOR call*
chr1	23703657	23703893	DEL;INS	DEL;INS
chr1	223672571	223672681	INS;INS	INS;INS
chr10	69539376	69539572	INS;INS	INS;INS
chr11	79190887	79191145	**REF**;**REF**	**DEL**;**INS**
chr11	128436913	128437081	INS;INS	INS;INS
chr14	84276747	84276903	**REF**;DEL	**INS**;DEL
chr15	70364402	70364587	INS;**NA**	INS;**INS**
chr16	3529535	3529854	REF;**DEL**	LC;**INS**
chr17	27525992	27526118	INS;INS	INS;INS
chr18	44544809	44545037	INS;INS	INS;INS
chr18	59081301	59081379	INS;**INS**	INS;**INS**
chr18	71198388	71198450	REF;**NA**	REF;**INS**
chr2	160426201	160426342	INS;INS	INS;INS
chr2	211860947	211861156	DEL;**NA**	DEL;**INS**
chr21	35063465	35063588	INS;INS	INS;INS
chr22	46174187	46174274	REF;INS	REF;INS
chr3	13856835	13857013	DEL;INS	DEL;INS
chr4	13807826	13807982	REF;**REF**	REF;**INS**
chr4	18837113	18837320	INS;DEL	INS;DEL
chr4	81637241	81637408	DEL;DEL	DEL;DEL
chr5	54513584	54513735	**REF**;INS	**INS**;INS
chr6	25450910	25450975	REF;**INS**	REF;**INS**
chr6	55543085	55543393	INS;INS	INS;INS
chr6	106945844	106946002	DEL;**DEL**	DEL;**INS**
chr7	38610247	38610412	**NA**;DEL	**INS**;DEL
chr7	71847696	71847865	INS;INS	INS;INS
chr7	109663557	109663744	INS;DEL	INS;DEL
chr7	131933466	131933651	INS;INS	INS;INS
chr9	82850174	82850347	DEL;DEL	DEL;DEL
chr9	91622218	91622365	**NA**;NA	**INS**;REF
chr9	91634814	91634973	**NA**;**NA**	**DEL**;**INS**
chr9	116632126	116632280	INS;INS	INS;INS

* DEL indicates a deletion; INS indicates an insertion; REF indicates a reference allele; NA indicates that the region is not covered by the assembly or mis-assembled; LC indicates that TRiCoLOR could not generate a consensus sequence for the allele due to the low coverage in the region. The 2 alleles are separated by a semicolon.

[30]. As shown in Table 1, out of 58 non-reference TR alleles identified by TRiCoLOR, we could visually confirm 42 (~75%) of them in the HGSVC assembly, which means that both TRiCoLOR and the HGSVC predicted the same variant type (deletion or insertion) and the predicted variant size is roughly similar (*i.e.* the difference does not exceed 50 bps). However, for the other 16 variants (~25%), the HGSVC assembly either did not contain the allele predicted by TRiCoLOR or did not cover the investigated region, which suggests that mapping-based and assembly-based approaches can be complementary for TR detection using long reads.

Discussion

TRiCoLOR is a comprehensive TR caller for long reads that supports the *de novo* identification of TRs in whole-genome sequencing data. TRiCoLOR profiles TRs through an efficient POA algorithm combined with a RegEx-based string matching search, facilitating a robust and accurate discovery of the full spectrum of expanded and contracted TRs in personal genomes.

In comparison to previous tools, TRiCoLOR works with ONT and PB data seamlessly. TRiCoLOR also identifies TRs *de novo* and does not require *a priori* knowledge of annotated TR regions. The unique combination of features for genome-wide, *de novo* discovery and genotyping of TRs in ONT and PB data is to the best of our knowledge unmet by any other TR caller for long-read data. Besides the detection of TRs, TRiCoLOR visualizes TRs in their haplotype context and it can infer parental genotypes using low-coverage parental sequencing data.

TRiCoLOR has been designed for diploid organisms (Supplementary Note S8) and future work includes extending its feature set to polyploid species and haploid chromosomes (human Y chromosome). As a mapping-based approach, TRiCoLOR cannot identify repeats in unassembled regions of the genome (*e.g.*, human centromeres and telomeres). Furthermore, the entropy threshold and window size for the *de novo* identification of repetitive stretches that we empirically estimated is well-suited for short repeated motifs (2-3 bps) but may need adjustments for long motifs of higher nucleotide complexity. Lastly, by default TRiCoLOR profiles TRs with motif lengths $\leq$ 6 bps (also known as micro-satellites), excluding those with motif lengths $\geq$7 bps (also known as mini-satellites), which are less abundant in diploid organisms [31]. The RegEx algorithm can be also tuned to profile mini-satellites (*i.e.*, by extending the –*size* parameter) but TRiCoLOR has been extensively applied so far only to micro-satellites.

Given these limitations, future work will focus on extending TRiCoLOR to other ploidies, broadening the size spectrum of detectable repeat motif lengths and taking advantage of improved sequencing read accuracy (*e.g.*, high-fidelity long reads from PB). The latter directly improves the RegEx-based identification of repeats employed by TRiCoLOR and we thus believe TRiCoLOR is well-suited to characterize the TR landscape in present and future long-read data sets, making it an instrumental tool to robustly decipher the multiplicity of TRs in repeat-mediated clinical disorders.

Availability of source code and requirements

- Project name: TRiCoLOR (SciCrunch RRID: SCR_018801; bio.tools ID: tricolor)
- Project home page: https://github.com/davidebolo1993/TRiCoLOR. A dockerized version of TRiCoLOR is available at https://hub.docker.com/r/davidebolo1993/tricolor. Online documentation is available at https://davidebolo1993.github.io/tricolordoc.
- Operating system: Unix
- Programming languages: Python, Bash, C++
- Other requirements: Python 3.6 or higher, GCC 4.8 or higher and CMake 3.2 or higher.
- License: GNU Lesser General Public License 3.0

Availability of supporting data and materials

HGSVC whole-genome long-read sequencing data are available on the HGSVC website (https://www.internationalgenome.org/human-genome-structural-variation-consortium). Specifically:

- ONT FASTQ files: http://ftp.ebi.ac.uk/1000g/ftp/data_collections/hgsv_sv_discovery/working/20181210_ONT_rebasecalled
- PB alignments: http://ftp.ebi.ac.uk/1000g/ftp/data_collections/hgsv_sv_discovery/working/20180102_pacbio_blasr_reheader
- Phased single-nucleotide variants: http://ftp.ebi.ac.uk/1000g/ftp/data_collections/hgsv_sv_discovery/working/20170323_Strand-seq_phased_FB%2BGATK_VCFs
- Illumina FASTQ files: http://ftp.ebi.ac.uk/1000g/ftp/data_collections/hgsv_sv_discovery/illumina_wgs.sequence.index
- HG00733 phased contigs: http://ftp.ebi.ac.uk/1000g/ftp/data_collections/hgsv_sv_discovery/working/20180227_PhasedSVGenomes
- HG00733 ground truth of structural variant calls:

http://ftp.ebi.ac.uk/1000g/ftp/data_collections/hgsv_sv_discovery/working/20180627_PanTechnologyIntegrationSet/HG00733.merged_nonredundant.vcf

The GRCh38 human reference genome used for alignments is available at http://ftp.ebi.ac.uk/1000g/ftp/technical/reference/GRCh38_reference_genome/GRCh38_full_analysis_set_plus_decoy_hla.fa. The corresponding annotated TRs can be accessed through the UCSC Table Browser tool (http://genome.ucsc.edu).
A whole-genome ONT FASTQ file of the *Arabisopsis thaliana* KBS-Mac-74 is available at ftp://ftp.sra.ebi.ac.uk/vol1/fastq/ERR217/003/ERR2173373/ERR2173373.fastq.gz. The TAIR10 reference genome for *Arabidopsis thaliana* can be downloaded through the Arabidopsis Information Resource database (https://www.arabidopsis.org/index.jsp) Several scripts used to perform the analyses described in this paper and the TR calls generated by TRiCoLOR for the HGSVC individuals and the *Arabisopsis thaliana* KBS-Mac-74 are available through the GitHub code repository of TRiCoLOR (https://github.com/davidebolo1993/TRiCoLOR). More in detail:

- the https://github.com/davidebolo1993/TRiCoLOR/tree/master/paper/data folder contains the BED file with annotated TRs from the GRCh38 human reference genome (`GRCh38.TRs.bed`), a bash script that illustrates how to haplotype-resolve a long-read alignment using phased single-nucleotide variants (`prepare.sh`), a python script used for the Shannon entropy simulations (`entropy.py`), a python script used to calculate precision, recall and F1 scores of TRiCoLOR on synthetic data (`pr.py`) and a couple of C++ source code files (`fmindex.cpp` and `validate.cpp`) for validating TRiCoLOR calls on real human data.
- the https://github.com/davidebolo1993/TRiCoLOR/tree/master/paper/samples folder contains TRiCoLOR calls for the HGSVC individuals and the *Arabisopsis thaliana* KBS-Mac-74 in standard BCF format.

A snapshot of the archival code is available in the *GigaScience* GigaDB database [32].

Declarations

List of abbreviations

TR: tandem repeat; ONT: Oxford Nanopore Technologies; PB: Pacific Biosciences; POA: partial order alignment; RegEx: regular expression; HGSVC: Human Genome Structural Variation Consortium; P: precision; R: recall; F1: F1 score.

Competing Interests

The authors declare that they have no competing interests.

Funding

JOK is supported by GraphGenomes grant 031L0184C. AM is supported by AIRC grant 20307. The funders had no role in study design, data collection and analysis, decision to publish, or preparation of the manuscript.

Author's Contributions

DB and TR designed and benchmarked the software. DB wrote the code. TR supervised the work. DB and TR co-wrote the manuscript draft. AM, VB and JOK contributed to the interpretation of the results, provided critical feedback and helped to write the manuscript. All the authors read and approved the manuscript.

Acknowledgements

The authors thank HGSVC for data access, and EMBL GeneCore and IT for technical support.

References

1. de Koning APJ, Gu W, Castoe TA, Batzer MA, Pollock DD. Repetitive elements may comprise over Two-Thirds of the human genome. PLoS Genetics 2011;.
2. Hannan AJ, Tandem repeats mediating genetic plasticity in health and disease; 2018.
3. Paulson H. Repeat expansion diseases. In: Handbook of Clinical Neurology; 2018.
4. Gymrek M, Golan D, Rosset S, Erlich Y. lobSTR: A short tandem repeat profiler for personal genomes. Genome Research 2012;.
5. Highnam G, Franck C, Martin A, Stephens C, Puthige A, Mittelman D. Accurate human microsatellite genotypes from high-throughput resequencing data using informed error profiles. Nucleic Acids Research 2013;.
6. Doi K, Monjo T, Hoang PH, Yoshimura J, Yurino H, Mitsui J, et al. Rapid detection of expanded short tandem repeats in personal genomics using hybrid sequencing. Bioinformatics 2014;.
7. Velasco A, James BT, Wells VD, Girgis HZ, Elofsson A. Look4TRs: A de novo tool for detecting simple tandem repeats using self-supervised hidden Markov models. Bioinformatics 2020;.
8. Chaisson MJP, Sanders AD, Zhao X, Malhotra A, Porubsky D, Rausch T, et al. Multi-platform discovery of haplotype-resolved structural variation in human genomes. Nature Communications 2019;.
9. Ummat A, Bashir A. Resolving complex tandem repeats with long reads. Bioinformatics 2014;.
10. Harris RS, Cechova M, Makova KD, Birol I. Noise-cancelling repeat finder: Uncovering tandem repeats in error-prone long-read sequencing data. Bioinformatics 2019;.
11. Gao Y, Liu B, Wang Y, Xing Y. TideHunter: Efficient and sensitive tandem repeat detection from noisy long-reads using seed-and-chain. In: Bioinformatics; 2019. .
12. De Roeck A, De Coster W, Bossaerts L, Cacace R, De Pooter T, Van Dongen J, et al. NanoSatellite: Accurate characterization of expanded tandem repeat length and sequence through whole genome long-read sequencing on PromethION. Genome Biology 2019;.
13. Mitsuhashi S, Frith MC, Mizuguchi T, Miyatake S, Toyota T, Adachi H, et al. Tandem-genotypes: robust detection of tandem repeat expansions from long DNA reads. Genome Biology 2019;.
14. Lee C, Grasso C, Sharlow MF. Multiple sequence alignment using partial order graphs. Bioinformatics 2002;.
15. Lee C. Generating consensus sequences from partial order multiple sequence alignment graphs. Bioinformatics 2003;.
16. Tenreiro MacHado JA. Shannon entropy analysis of the genome code. Mathematical Problems in Engineering 2012;.
17. Vaser R, Sović I, Nagarajan N, Šikić M. Fast and accurate de novo genome assembly from long uncorrected reads. Genome Research 2017;.

18. Li H. Minimap2: Pairwise alignment for nucleotide sequences. Bioinformatics 2018;.
19. Bolognini D, Sanders A, Korbel JO, Magi A, Benes V, Rausch T. VISOR: A versatile haplotype-aware structural variant simulator for short-and long-read sequencing. Bioinformatics 2020;.
20. Bolognini D, Bartalucci N, Mingrino A, Vannucchi AM, Magi A. NANOR: A user-friendly R package to analyze and compare nanopore sequencing data. PLoS ONE 2019;.
21. Li H, Handsaker B, Wysoker A, Fennell T, Ruan J, Homer N, et al. The Sequence Alignment/Map format and SAMtools. Bioinformatics 2009;.
22. Rausch T, Hsi-Yang Fritz M, Korbel JO, Benes V. Alfred: Interactive multi-sample BAM alignment statistics, feature counting and feature annotation for long- and short-read sequencing. Bioinformatics 2019;.
23. Pedersen BS, Quinlan AR. Mosdepth: Quick coverage calculation for genomes and exomes. Bioinformatics 2018;.
24. Edge P, Bansal V. Longshot enables accurate variant calling in diploid genomes from single-molecule long read sequencing. Nature Communications 2019;.
25. Mantere T, Kersten S, Hoischen A, Long-read sequencing emerging in medical genetics; 2019.
26. Dolle DD, Liu Z, Cotten M, Simpson JT, Iqbal Z, Durbin R, et al. Using reference-free compressed data structures to analyze sequencing reads from thousands of human genomes. Genome Research 2017;.
27. Ferragina P, Manzini G. Opportunistic data structures with applications. In: Annual Symposium on Foundations of Computer Science - Proceedings; 2000. .
28. Giner-Delgado C, Villatoro S, Lerga-Jaso J, Gayà-Vidal M, Oliva M, Castellano D, et al. Evolutionary and functional impact of common polymorphic inversions in the human genome. Nature Communications 2019;.
29. Mikheenko A, Prjibelski A, Saveliev V, Antipov D, Gurevich A. Versatile genome assembly evaluation with QUAST-LG. In: Bioinformatics; 2018. .
30. Thorvaldsdóttir H, Robinson JT, Mesirov JP. Integrative Genomics Viewer (IGV): High-performance genomics data visualization and exploration. Briefings in Bioinformatics 2013;.
31. Richard GF, Kerrest A, Dujon B. Comparative Genomics and Molecular Dynamics of DNA Repeats in Eukaryotes. Microbiology and Molecular Biology Reviews 2008;.
32. Bolognini D, Magi A, Benes V, , Korbel JO, Rausch T. Supporting data for "TRiCoLOR: tandem repeat profiling using whole-genome long-read sequencing data". GigaScience Database 2020;http://dx.doi.org/10.5524/100790.

Figure 1

Click here to access/download;Figure;Fig1.pdf

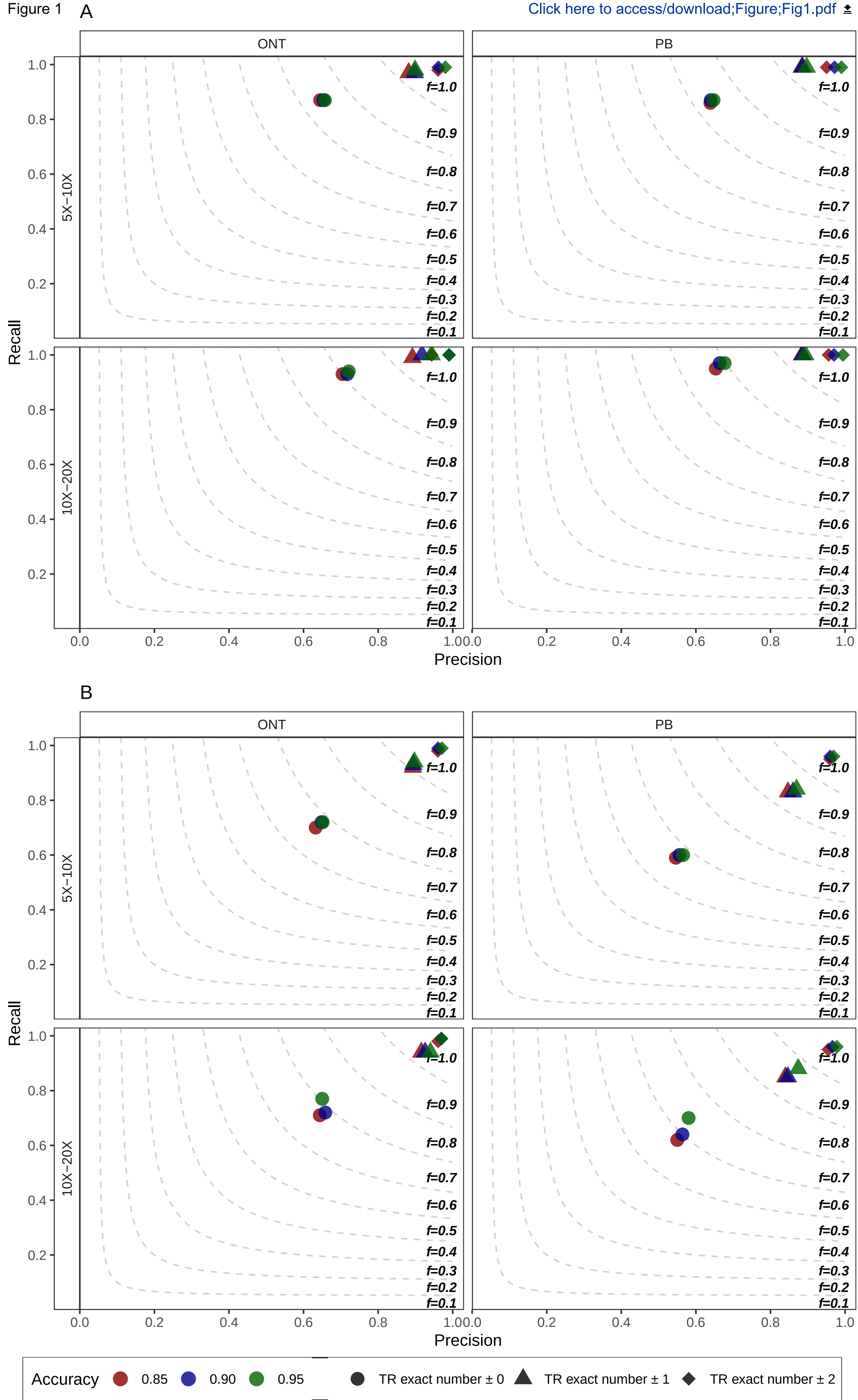

Figure 2

Click here to access/download;Figure;Fig2.pdf

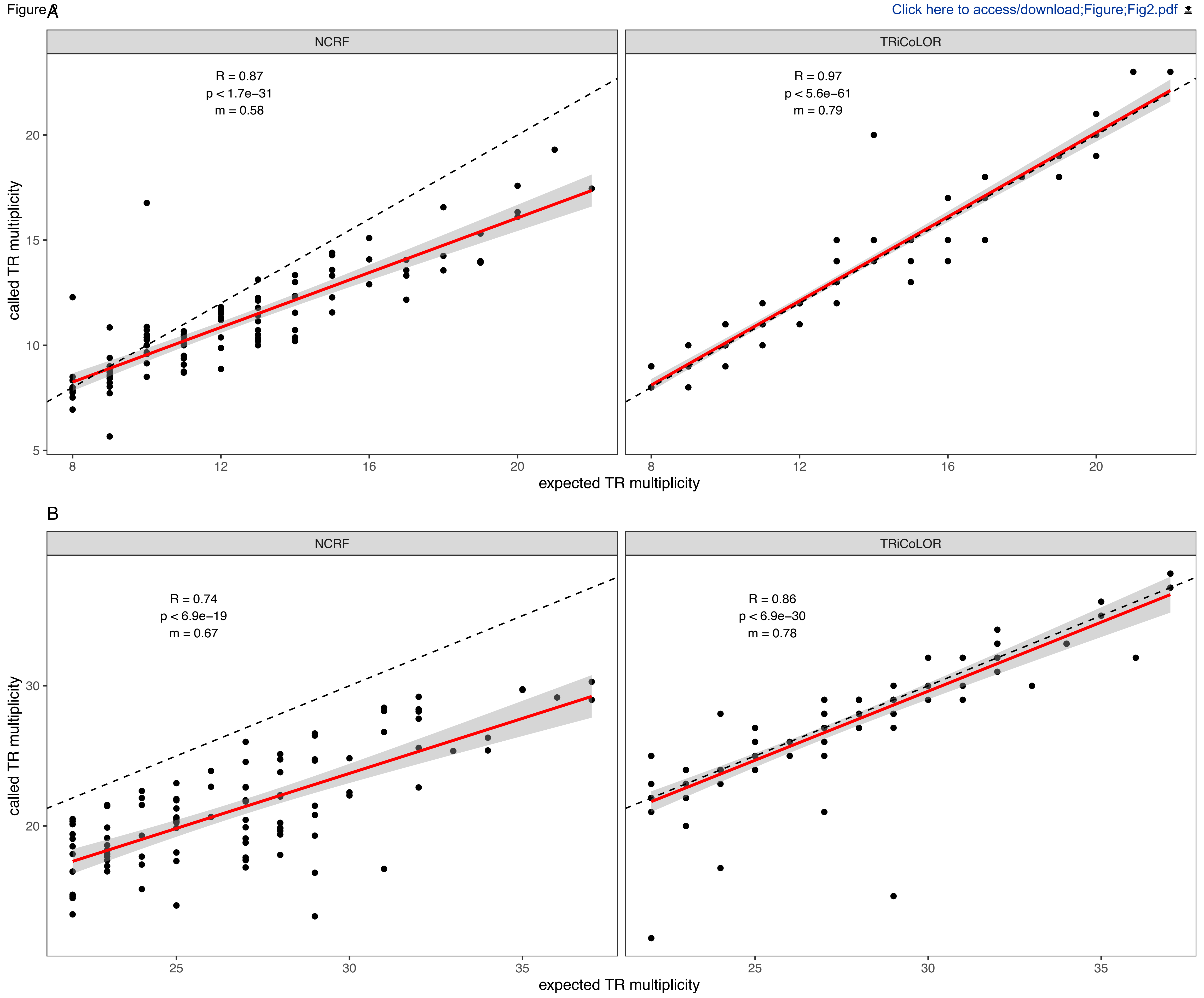

Click here to access/download
Supplementary Material
TRiCoLOR_supplementary.pdf

Personal Cover
Click here to access/download;Personal Cover;TRiCoLOR_cover.pdf

Date: September 3, 2020

Manuscript: GIGA-D-20-00168R2

Dear Editor,

please find enclosed the revised version of our manuscript ”TRiCoLOR: tandem repeat profiling using whole-genome long-read sequencing data” for your kind consideration as a *GigaScience* Technical Note.
We have addressed the minor edits required to meet *GigaScience*’s formatting and reproducibility guidelines, as from the decision letter. Thank you for publishing our revised manuscript in *GigaScience*.

Best regards and on behalf of the authors,

Davide Bolognini
