# Supplementary material for: TRiCoLOR: tandem repeat profiling using whole-genome long-read sequencing data
Source: Gigascience. 2020 Oct 7;9(10):giaa101. doi: 10.1093/gigascience/giaa101 (PMC7539535; doi:10.1093/gigascience/giaa101)
Supplement: giaa101_Supplemental_File [file giaa101_supplemental_file.pdf]

Supplementary Material
TRiCoLOR: tandem repeat profiling using whole-genome long-read sequencing data

Davide Bolognini, Alberto Magi, Vladimir Benes,
Jan O. Korbel, Tobias Rausch

Supplementary Notes

Note S1

The ability of TRiCoLOR (Tandem Repeats Caller for LOng Reads) to determine the phase and genotype of tandem repeats (TRs) in diploid samples strictly relies on the *a priori* knowledge of the hapolotype that aligned reads belong to, *i.e.* their phase. The term *phasing* refers to assigning individual's haplotypes by identifying short variants (single-nucleotide variants and indels) that lie near each other on the same chromosome and are inherited together [1]. Short variants can be reliably detected using whole-genome short-read sequencing [2] but resolving haplotypes with such a technology has limitations because two adjacent heterozygous variants are usually not spanned by a single sequenced fragment to allow a so-called read-backed phasing procedure. Read-backed phasing assembles haplotypes using overlaps between reads that span multiple heterozygous variants but since the heterozygosity ratio of the human genome is comparatively low [3] the average nucleotide distance of heterozygous markers exceeds the short read length. Long reads have been reported valuable to overcome these limitations and resolve individual's haplotypes either alone or in combination with other sequencing technologies.
As a proof of concept, we evaluated the performances of different frameworks for generating haplotype-resolved alignments prior to TRiCoLOR. First, we exploited VISOR [4] to insert phased single-nucleotide variants from the 1000 Genomes Project (HG00732 sample) on chr20 of the GRCh38 human reference genome and to simulate a final BAM file (∼40X coverage) mirroring Oxford Nanopore Technologies (ONT) data (see the **Findings** section in the main text). Then, we applied WhatsHap (version 1.0) [5] and LongShot (version 0.4.1) [6] to directly identify candidate single nucleotide variants from the synthetic BAM file, and phase them. We run WhatsHap's *find_snv_candidates* module with the *–nanopore* parameter enabled, the *genotype* module, the *phase* module and the *haplotag* module sequentially, using the default parameter settings. We run

LongShot with the default parameter settings as well. WhatsHap and LongShot could assign respectively $\sim 68\%$ and $\sim 71\%$ of the synthetic ONT reads to one of the 2 haplotypes and $\sim 89\%$ of all sequenced bases because most unassigned reads are relatively short (as expected). We measured the amount of phasing inconsistencies between the phased single-nucleotide variants from the 1000 Genomes Project and those from WhatsHap and Longshot by calculating their switch error rates [7] using vcftools [8]. The calculated switch error rate of WhatsHap was $\sim 1.2\%$, while the switch error rate of LongShot was $\sim 0.9\%$ We also evaluated the capability of HapCUT2 (version 1.2) [9] and WhatsHap to phase the synthetic ONT BAM file using complementary single-nucleotide variant calls generated by bcftools [10] from a short-read alignment (~40X coverage) simulated with VISOR for the same sample (these calls from bcftools should represent a set of variant calls one can be reasonably confident in). We run the *extractHAIRS* and *hapcut2* commands from the HapCUT2 package and the *phase* and the *haplotag* modules from WhatsHap, using the default parameter settings. Because HapCUT2 does not provide utilities to either tag or split reads by haplotype, we subsequently resolved the 2 haplotypes of the ground truth BAM file using Alfred (version 0.1.18) [11], giving the phased single-nucleotide variants from HapCUT2 as input. WhatsHap and Alfred could assign $\sim 71\%$ of the synthetic ONT reads to one of the 2 haplotypes ($\sim 92\%$ of sequenced bases). Using this experimental setting, the calculated switch error rate of WhatsHap was $\sim 0.7\%$ while the switch error rate of HapCut2 was $\sim 0.6\%$, reflecting the higher-quality of input single-nucleotide variants.
Overall the local phase accuracy of long-read phasing algorithms is high. However, long-read technologies alone are sub-optimal for chromosomal-level phasing, for which they have to be used in combination with a chromosome-scale technology such as Strand-seq or Hi-C, as for the HGSVC data [12] that we used in this paper. TRiCoLOR can be applied to read-backed phased data as well as chromosome-length haplotypes because it evaluates each tandem repeat locally in a surrounding window, where results are only expected to deteriorate if a rare switch error occurred within a given tandem repeat window. A bash script that briefly illustrates the pipeline to generate haplotype-resolved alignments from phased variants is available in the GitHub code repository of TRiCoLOR (see **Availability of supporting data and materials** in the main text).

Note S2

Shannon entropy (H) was originally devised by Claude Shannon to study the amount of information in a transmitted message and can be defined as

$$H = -\sum_{x \in X} p_x \log_2 p_x$$

where x is any letter (DNA base) from the transmitted message (DNA sequence). p_x is the probability that $x \in X$ occurs (the frequency of the DNA base in the DNA string). Given this formula, a fully random sequence results

in the maximal H whereas a repetitive sequence "overuses" certain nucleotides which causes a low H, with perfect homopolymer runs having $H = 0$.
In order to identify the optimal H score (Ho) to discriminate between repetitive and non repetitive regions in error-prone long reads, we used VISOR to simulate 1000 long-read BAM files, with half of the BAM files modelling current sequencing error rates from Oxford Nanopore Technologies and the other half from Pacific Biosciences (PB), illustrated in panels A and B of Figure S2. We simulated ∼8000 bps reads at ∼10x coverage in small regions (∼20000 bps) around known, randomly chosen, TRs from the GRCh38 human reference genome. We then computed H for all aligned reads in non-overlapping, sliding windows of 20 bps. Given that the mean length of the known TRs for the GRCh38 genome is ∼40 bps, we chose a window size of 20 bps as this allows to have at least one window encompassed by the TRs picked during the simulations. We empirically set the Ho to be the 2nd percentile of the H distribution. Figure S1 shows the negatively-skewed H distributions for the simulated ONT (panel A) and PB (panel B) BAM files: we identified $Ho \sim 1.23$ for both of the simulated groups; these Ho value allowed to exclude $\sim 98\%$ of the entire alignment information screened, in accordance with the results presented by Gymrek *et al.* [13].
Figure S1 also illustrates the read-specific H in non-overlapping, sliding windows of 20 bps for a simulated ONT (panel C) and PB (panel D) BAM file including the TR ranging from 48941985 and 48942028 on chr19 of the GRCh38 human reference genome. All the simulated reads have at least one window where H is below the Ho in the region containing the TR (highlighted in green) while H is confirmed above the Ho threshold for the other regions.

Note S3

We evaluated the capability of SPOA[14], a single-instruction multiple-data version of the partial order alignment (POA) framework [15, 16], to reduce the error rate of long-read alignments using data from HGSVC. In particular, we applied Alfred to haplotype-resolved BAM files and calculated the error rate of the HG00733, HG00514 and NA19240 individuals, sequenced using platforms from ONT as well as from PB. We then applied SPOA to generate consensus sequences from a region on chr20 (18000000-20000000) of the HG00733 individual, using windows of 2000 bps (1000 windows in total); the computed consensus sequences were aligned to the GRCh38 human reference genome and we derived their error rate as for the original BAM file using Alfred. For the consensus generation, we exploited the "global" mode of SPOA with default penalties (matches: +5; mismatches: -4; gap opening: -8; gap extending: -6), which resulted in the lowest consensus error rates.
Figure S2 shows the error profiles of the ONT (panel A) and PB (panel B) alignments for the HG00733, HG00514 and NA19240 individuals, which are also haplotype-resolved (*e.g.* HG00733 is haplotype-resolved in HG00733.h1 and HG00733.h2). Before error correction, the mean error rate of ONT alignments

is ∼11% and the substitution:insertion:deletion ratio is ∼45:25:30; for PB alignments, the initial mean error rate is ∼13% and the substitution:insertion:deletion ratio is ∼15:50:35. Figure S2 also illustrates how much of the initial error rate of the ONT (panel C) and PB (panel D) alignments SPOA can correct. For the regions investigated, after the SPOA-mediated correction, the mean error rate of the generated alignments is drastically reduced: ∼2.5% (substitution:insertion:deletion ratio ∼31:37:32) for the ONT reads and ∼1.5% (substitution:insertion:deletion ratio ∼17:70:13) for the PB reads.

Note S4

In order to identify the best aligner for long sequences, we evaluated the performances of the widely-used mappers minimap2 (version 2.17-r941) [17] and NGMLR (version 0.2.7) [18]. We run minimap2 using the presets *-x map-ont* for the ONT simulations and *-x map-pb* for the PB simulations; we run NGMLR using the presets *-x ont* for the ONT simulations and *-x pb* for the PB simulations.

First, we evaluated the speed of the chosen aligners when aligning an increasing number of long sequences (coverage ∼1X, ∼5x, ∼10X, ∼15X, ∼20x, ∼25X, ∼30X; substitution:insertion:deletion ratio ∼10:60:30; average length of reads ∼8000 bps; accuracy of reads ∼0.90), simulated from a region on chr20 (32000000-62000000) of the GRCh38 human reference genome using PBSIM (version 1.0.3) [19] For each simulation (one for each coverage level), we repeated the alignment step 5 times using 6 Intel®Xeon®processors X5460 (clock rate 2.93 GHz) on an Ubuntu 16.04.6 LTS desktop. As illustrated in panel A of figure S3, minimap2 proved to be ∼6 times faster than NGMLR. Results are shown as mean ± standard deviation.

We further evaluated the accuracy of the chosen aligners when mapping long sequences of increasing length (∼500 bps, ∼1000 bps, ∼5000 bps, ∼10000 bps) and increasing accuracy (∼0.85, ∼0.90, ∼0.95), simulated from the same region on chr20 (32000000-62000000) of the GRCh38 human reference genome with PBSIM.

Panel B and panel C of figure S3 show these findings for simulated ONT (substitution:insertion:deletion ratio ∼45:25:30) and PB (substitution:insertion:deletion ratio ∼15:50:35) alignments respectively. We used the ratio between the number of reads mapped in the region chosen for simulating and the total number of reads mapped as a measure of accuracy. The accuracy of both minimap2 and NGMLR increases as the length of the simulated reads increases, approaching ∼1.0 when the length of these reads is $\geq$ 5000 bps. For shorter reads, minimap2 demonstrates an accuracy higher than NGMLR on the ONT simulations and slightly lower on the PB simulations.

These results led us to choose minimap2 as the default aligner for TRiCoLOR, as it outperformed NGMLR in terms of speed without loosing the comparison in terms of mapping accuracy. We furthermore investigated which preset of the minimap2 aligner performed best for mapping the consensus sequences

generated through SPOA (see also Note S3) to the reference genome. Using the simulation schema described above, we generated synthtetic ONT and PB alignments from a region on chr1 (100000000-110000000) of the GRCh38 human reference genome, and we exploited SPOA to generate consensus sequences using windows of 1000 bps (10000 windows in total). We aligned the consensus sequences formed back to the chr1 reference sequence using the presets for noisy reads (*i.e.* *-x map-ont* for the ONT alignments and *-x map-pb* for the PB alignments) as well as the presets for the assembly-to-reference alignment (*i.e.* *-x asm5*, *-x asm10*, *-x asm20*). For all the presets used, minimap2 was able to properly map all the generated consensus sequences to their original location (*e.g.* a consensus sequence generated from the 11th window was properly mapped to the original region chr1:100010000-100011000). Given that the presets evaluated did not influence the mapping accuracy of minimap2, we decided to call the aligner from within TRiCoLOR using the presets for noisy reads.

Note S5

As a proof of concept, Figure S4 presents how to browse the HTML file generated by TRiCoLOR ApP for a TR (dinucleotide TG repeated 15 times) on chromosome 17 (64240234-64240263) of the GRCh38 human reference genome for which we simulated a small expansion (dinucleotide TG repeated 22 times) on haplotype 1 using VISOR.
Figure S4A shows the home screen of the HTML. Top left buttons allow users to highlight repetitions found in the reference and in the individuals' haplotypes. In addition to the TR of interest, we found in the browsed region a stretch of 28 As (64239876-64239903), which is also highlighted in the screenshot. To further resolve a TR, users can zoom into a certain area (*e.g.*, the area we selected with the red rectangle).
Figure S4B shows the sequences of the reference and the individual's haplotypes at higher magnification. Each dot corresponds to a single nucleotide and users can check the base composition of each molecule by simply scrolling across the alignment. Deletions of one or more bases in the haplotypes can be seen as gaps in their sequences while insertions are represented by multiple dots sharing the same coordinate (thus, closer than expected). We highlighted with a red rectangle the insertion of TG bases found on haplotype 1.
Figure S4C shows a further magnification of the extended TG repetition (TG repeated 7 times more, the area we selected with the red rectangle) that we simulated on haplotype 1. The inserted TGs share the same coordinate and are missing in the reference sequence and in haplotype 2.

Note S6

Precision (P), recall (R), and F1 score (F1) are defined as:

$$P = \frac{TP}{TP + FP}$$

$$R = \frac{TP}{TP + FN}$$

$$F1 = 2 * \frac{P * R}{P + R}$$

A true positive (TP) is a call for which the number of repetitions identified by TRiCoLOR matches the number of repetitions in the haplotype containing the TR contraction/expansion.
A false positive (FP) is a call for which the number of repetitions identified by TRiCoLOR does not match the number of repetitions in the unaltered haplotype.
A false negative (FN) is a call for which the number of repetitions identified by TRiCoLOR does not match the number of repetitions in the haplotype containing the TR contraction/expansion.

Note S7

We run TRiCoLOR REFER with the *-m* parameter set to the length of the repeated motif (*e.g.*, *-m 2* for a GT repetition), the *–precisemotif* parameter enabled and the *–readstype* parameter set to *ONT* for ONT simulations and to *PB* for the PB simulations.
We run NCRF (version 1.01.02) [20] using the authors' README suggestions with - -*scoring* parameter set to *nanopore* for ONT simulations and to *pacbio* for PB simulations; we adjusted the *–minlength* parameter accordingly to the minimum length of the TRs simulated. We averaged the number of repetitions found by NCRF in each FASTA.

Note S8

As a proof of concept, we applied TRiCoLOR to call TRs genome-wide on a publicly available whole-genome ONT sequencing dataset for the *Arabidospsis thaliana* KBS-Mac-74 model organism [21]. We aligned the ONT FASTQ file to the TAIR10 reference genome using minimap2. Because a matching Strand-seq data generated from the same *Arabidospsis* species as the ONT data is not available, we were not able to produce chromosome-length haplotypes for this sample as we did for the HGSVC individuals (see **Findings** in the main text). For chromosomes 1 to 5 of the *Arabidospsis thaliana* KBS-Mac-74, we therefore resolved haplotype blocks using the read-backed phasing strategy from WhatsHap (see Note S1). We calculated the coverage of the initial and the haplotype-resolved BAM files using mosdepth [22], identifying an initial ∼27X coverage, reduced

to $\sim$9X for each haplotype after splitting, due to some unassigned reads. We then run TRiCoLOR SENSoR and TRiCoLOR REFER sequentially, using the default parameter settings. The SENSoR module took $\sim$ 10 minutes to scan the *Arabidospsis thaliana* haplotypes and identified $\sim$ 2000 low-entropy regions (average length of the regions $\sim$800 bps). With 7 processors on our Ubuntu desktop, TRiCoLOR REFER completed the TR profiling step in $\sim$ 15 minutes. Raw TR calls in BCF format generated by TRiCoLOR for the *Arabidospsis thaliana* KBS-Mac-74 are available in the GitHub code repository of TRiCoLOR (see **Availability of supporting data and materials** in the main text).

Supplementary Figures

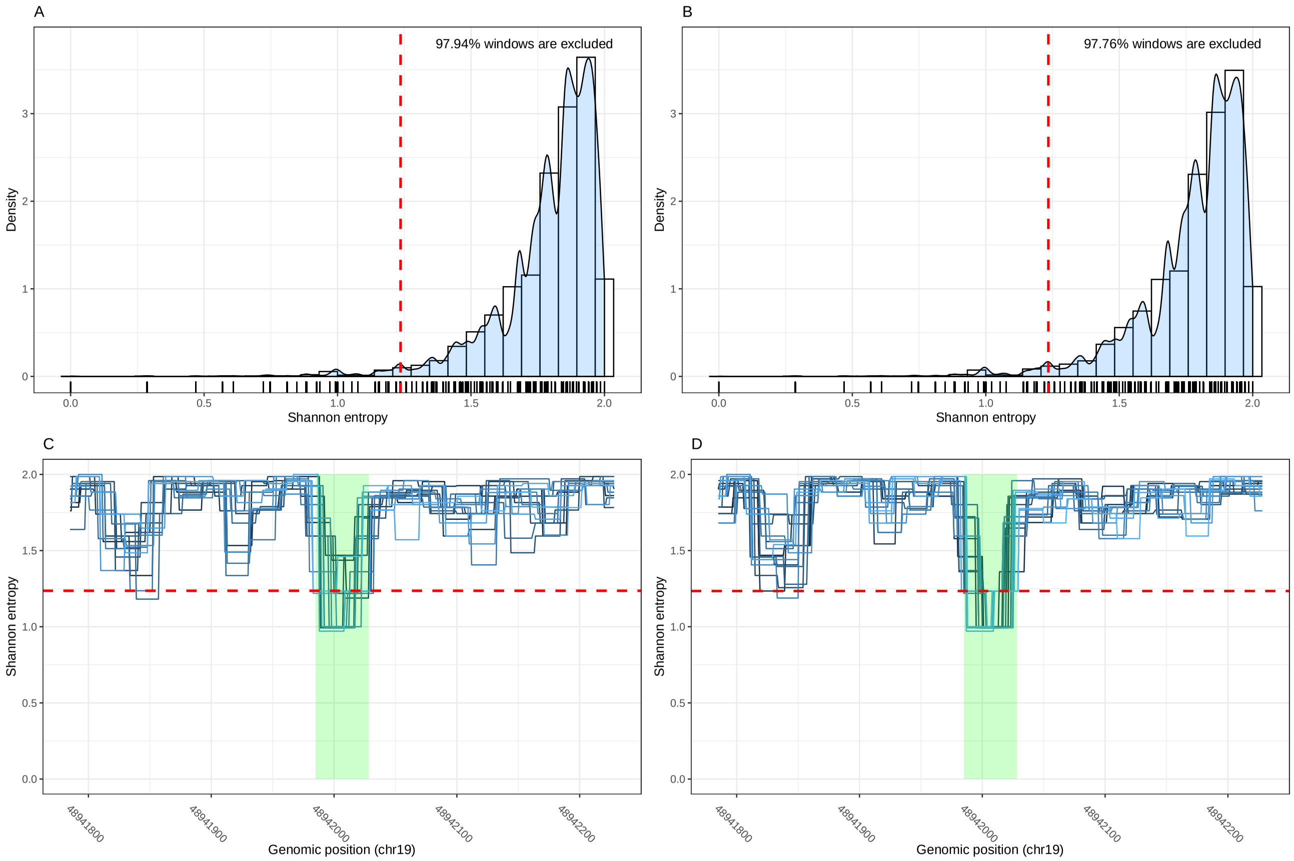

A
B
C
D
97.94% windows are excluded
97.76% windows are excluded
Density
Shannon entropy
Genomic position (chr19)
48941800
48941900
48942000
48942100
48942200

Figure S1

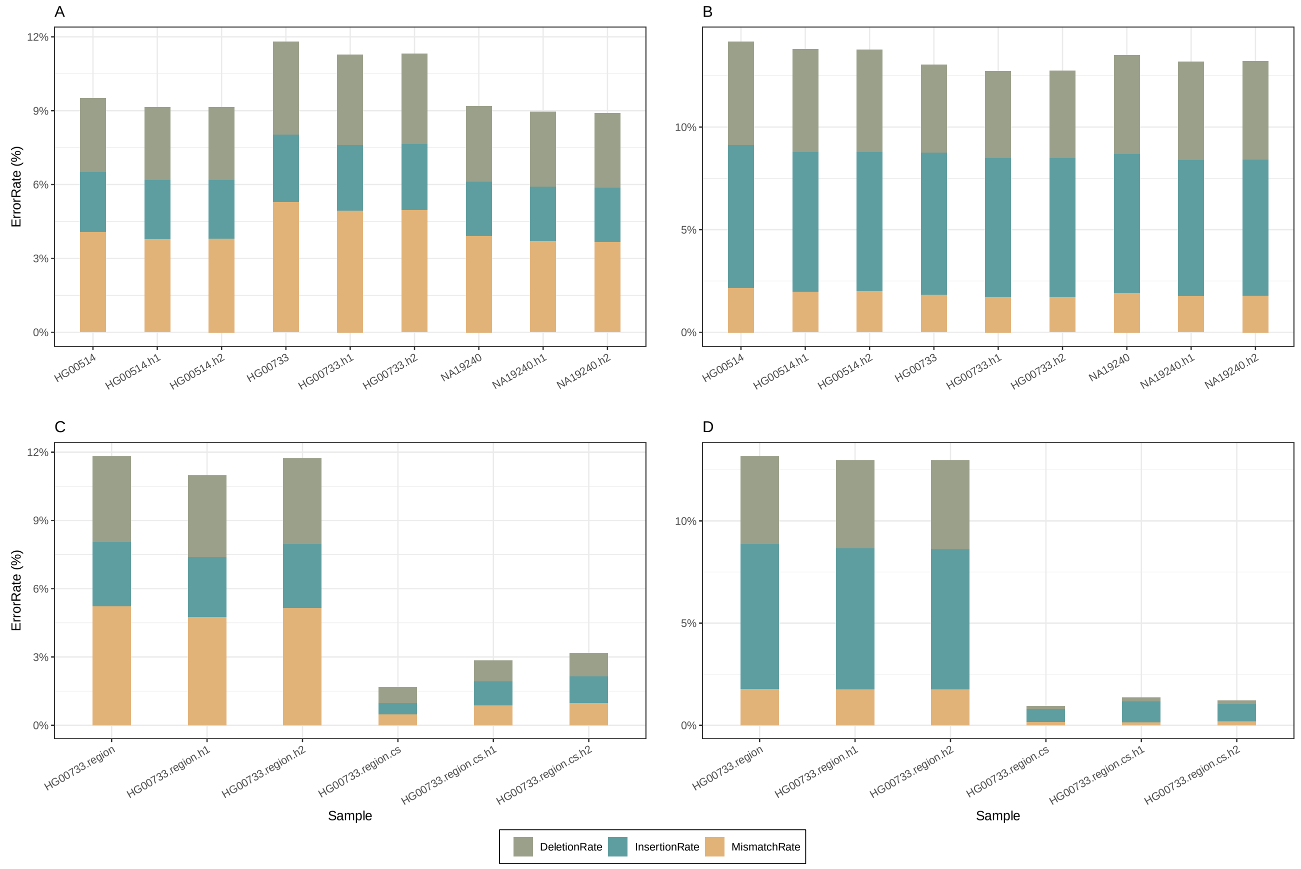
A
B
C
D
ErrorRate (%)
12%
9%
6%
3%
0%
10%
5%
HG00514
HG00514.h1
HG00514.h2
HG00733
HG00733.h1
HG00733.h2
NA19240
NA19240.h1
NA19240.h2
HG00733.region
HG00733.region.h1
HG00733.region.h2
HG00733.region.cs
HG00733.region.cs.h1
HG00733.region.cs.h2
Sample
DeletionRate
InsertionRate
MismatchRate

Figure S2

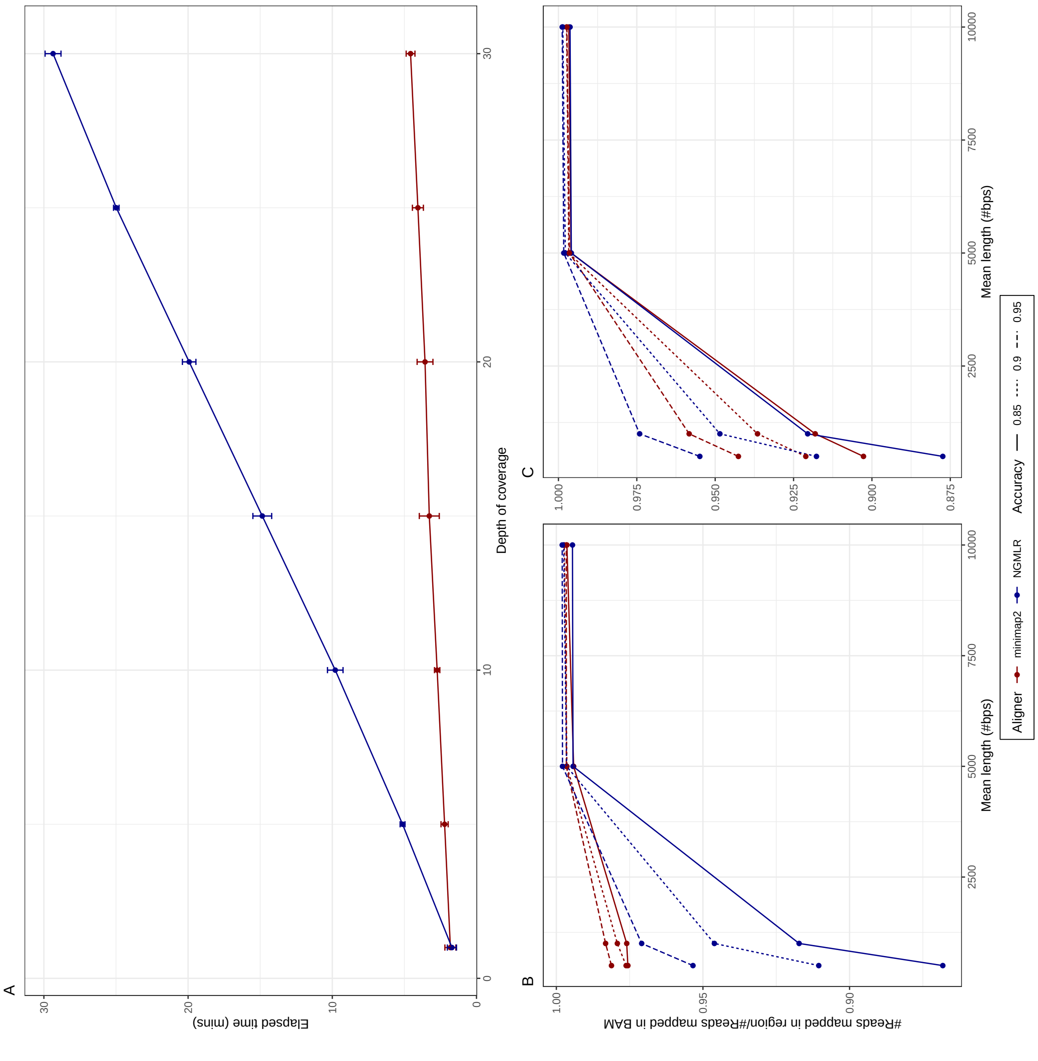
A
Elapsed time (mins)
0
10
20
30
Depth of coverage
B
C
#Reads mapped in region/#Reads mapped in BAM
1.00
0.95
0.90
1.000
0.975
0.950
0.925
0.900
0.875
2500
5000
7500
10000
Mean length (#bps)
Aligner
minimap2
NGMLR
Accuracy
0.85
0.9
0.95

Figure S3

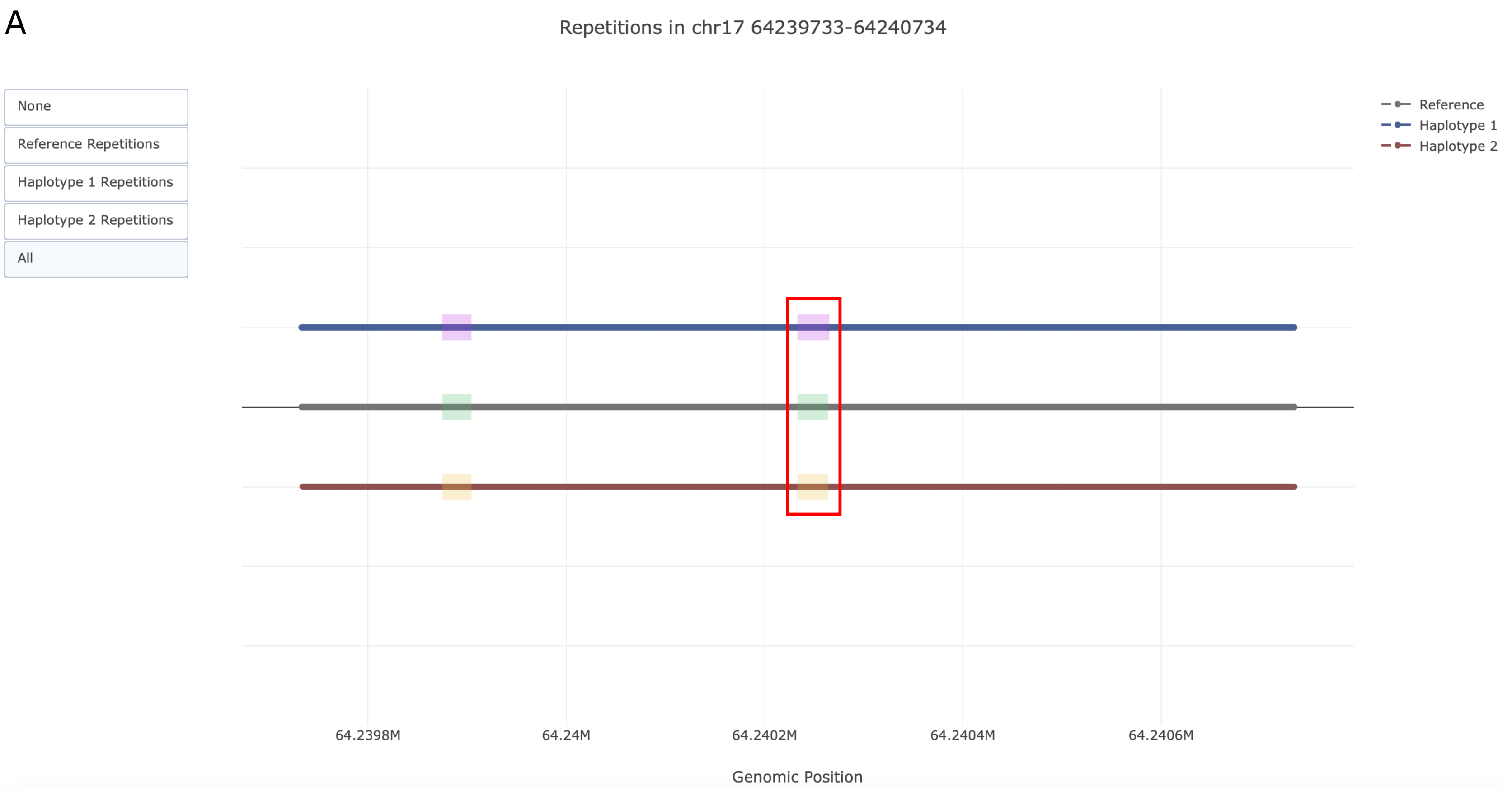
A
Repetitions in chr17 64239733-64240734
None
Reference Repetitions
Haplotype 1 Repetitions
Haplotype 2 Repetitions
All
Reference
Haplotype 1
Haplotype 2
64.2398M
64.24M
64.2402M
64.2404M
64.2406M
Genomic Position

Figure S4A

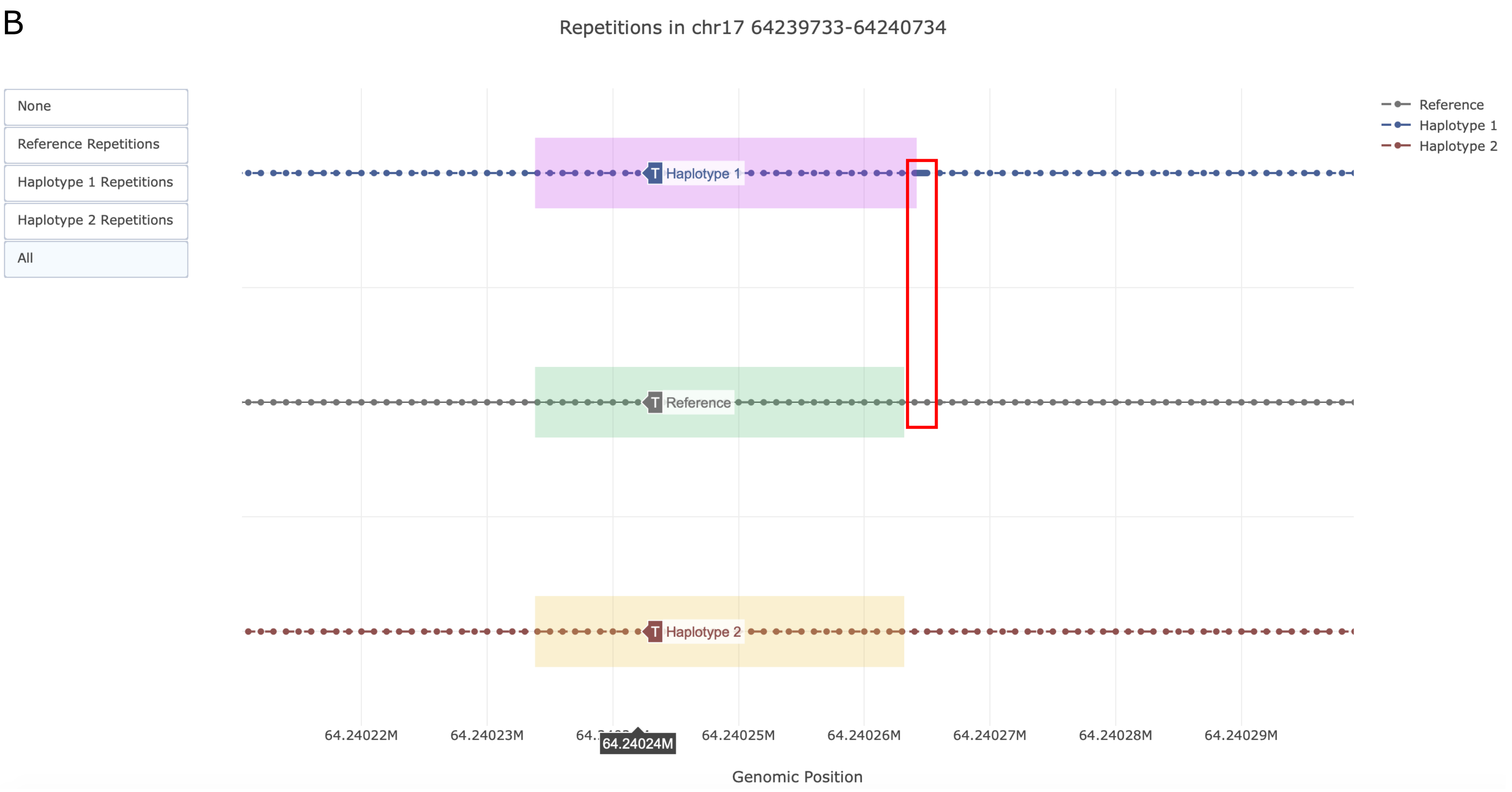
B
Repetitions in chr17 64239733-64240734
None
Reference Repetitions
Haplotype 1 Repetitions
Haplotype 2 Repetitions
All
Haplotype 1
Reference
Haplotype 2
Reference
Haplotype 1
Haplotype 2
64.24022M
64.24023M
64.24024M
64.24025M
64.24026M
64.24027M
64.24028M
64.24029M
Genomic Position

Figure S4B

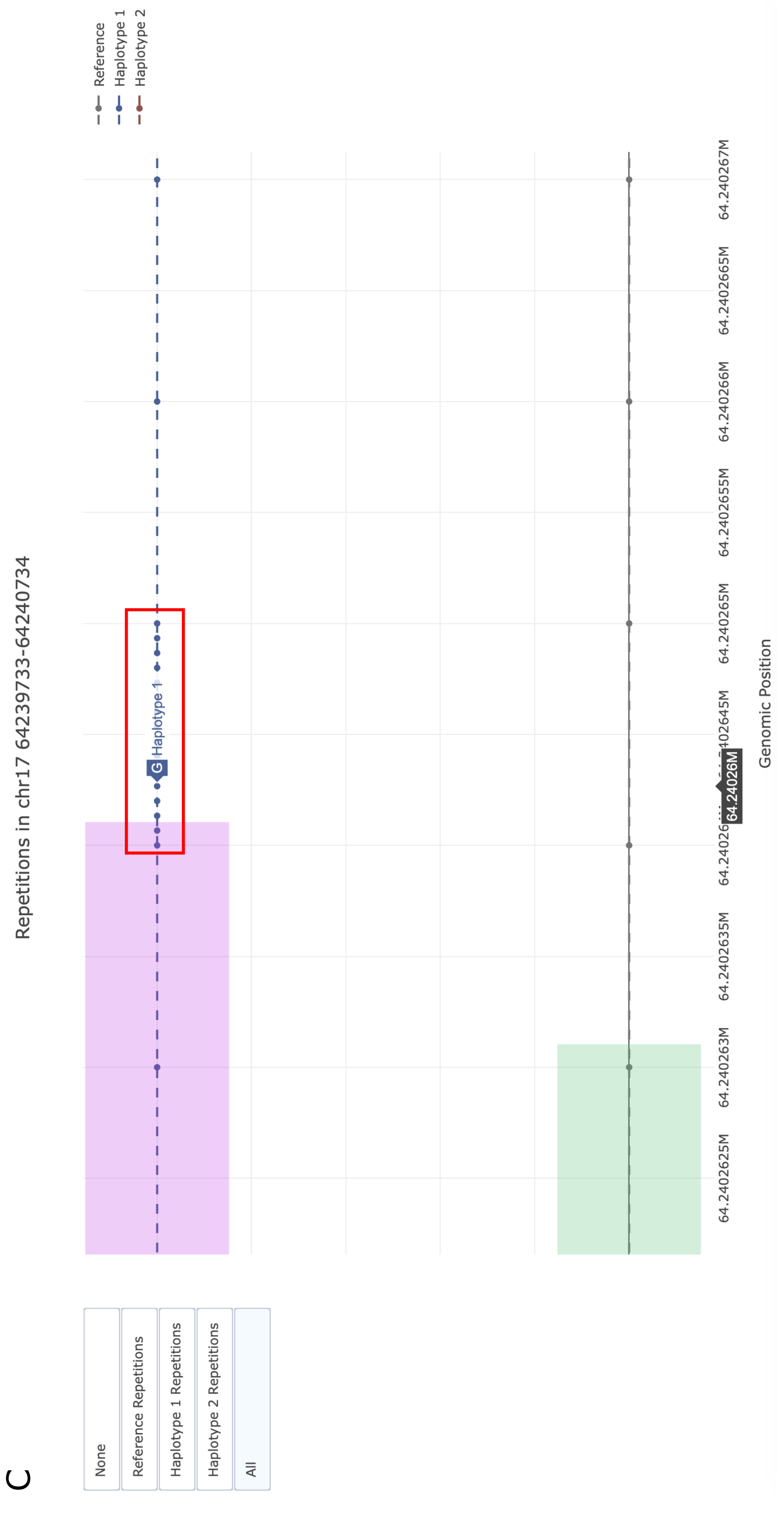
C
Repetitions in chr17 64239733-64240734
Reference
Haplotype 1
Haplotype 2
None
Reference Repetitions
Haplotype 1 Repetitions
Haplotype 2 Repetitions
All
Haplotype 1
G
64.24026M
64.2402625M
64.240263M
64.2402635M
64.240265M
64.2402655M
64.240266M
64.2402665M
64.240267M
Genomic Position

Figure S4C

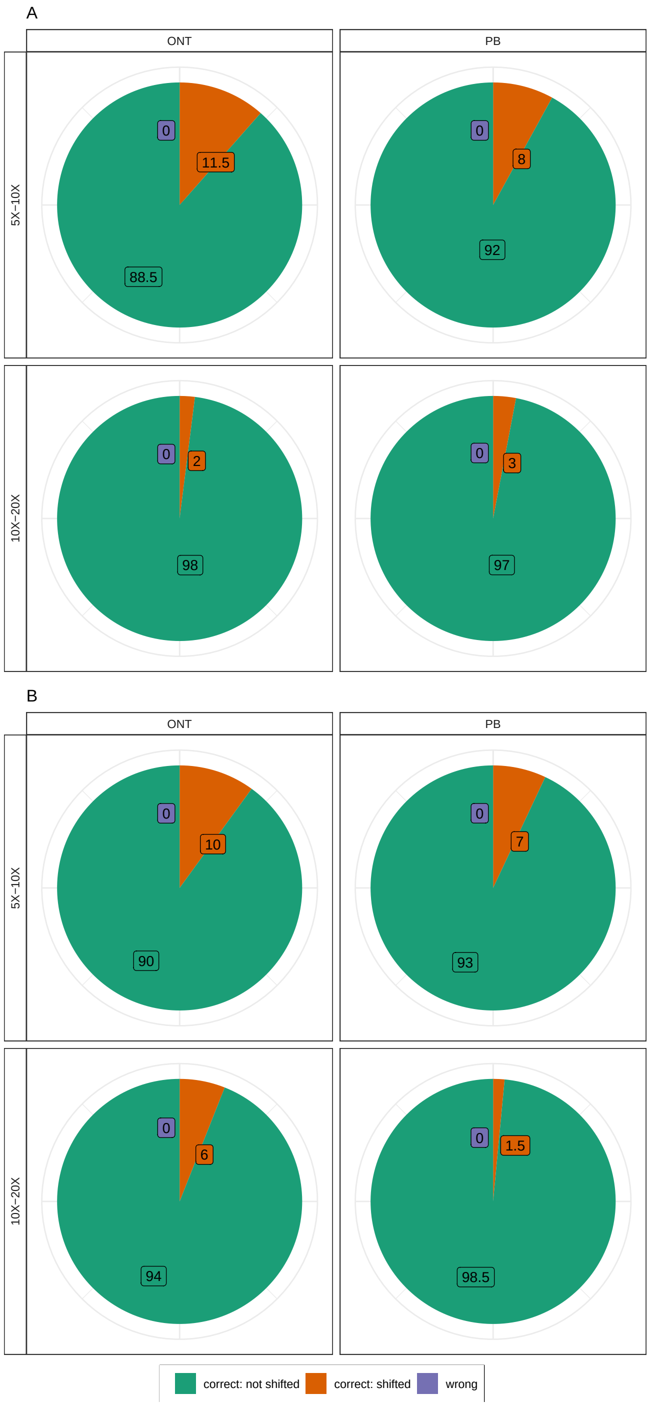
A
ONT
PB
5X–10X
10X–20X
0
11.5
88.5
0
8
92
0
2
98
0
3
97
B
ONT
PB
5X–10X
10X–20X
0
10
90
0
7
93
0
6
94
0
1.5
98.5
correct: not shifted
correct: shifted
wrong

Figure S5

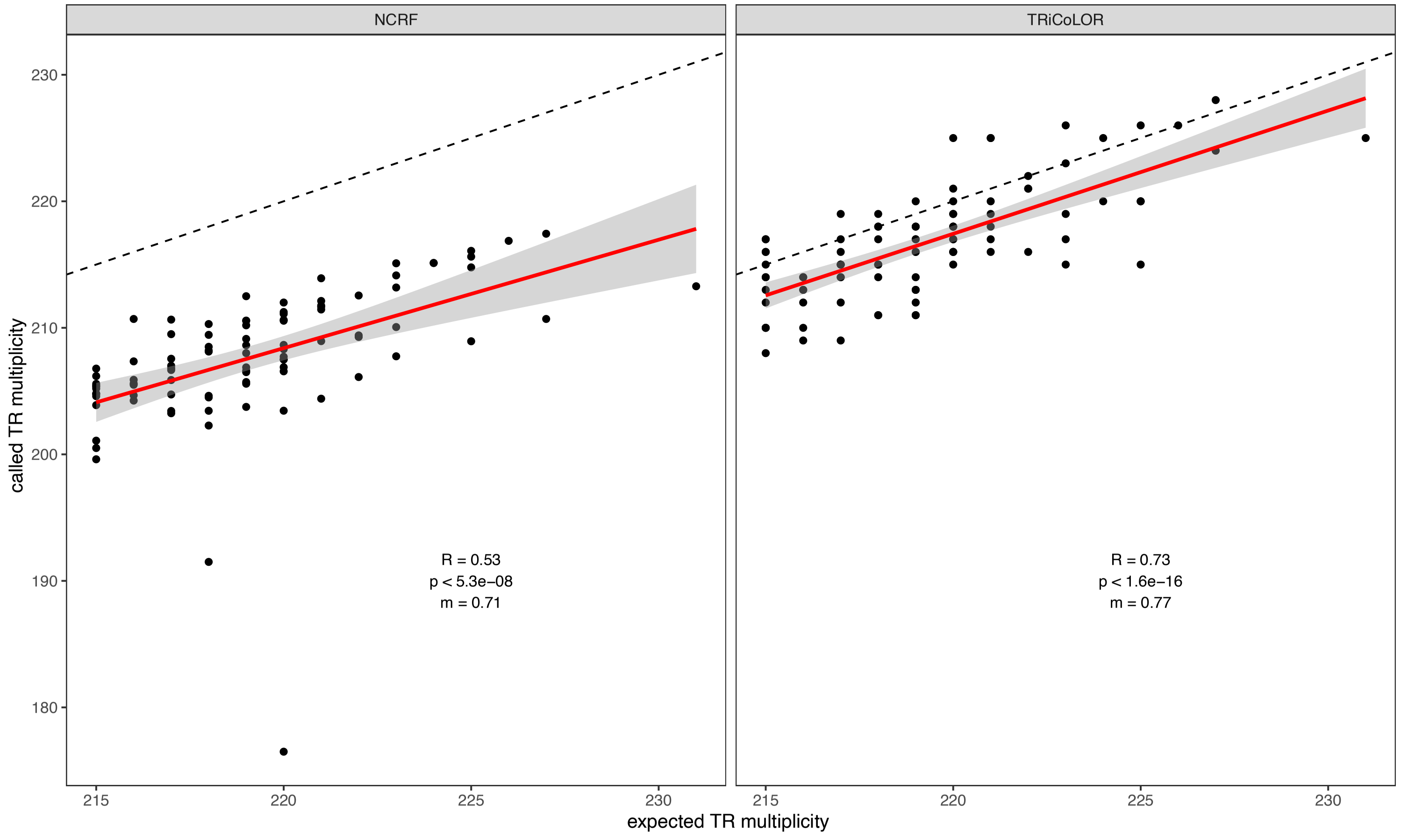

NCRF
TRiCoLOR
called TR multiplicity
230
220
210
200
190
180
expected TR multiplicity
215
220
225
230
R = 0.53
p < 5.3e−08
m = 0.71
R = 0.73
p < 1.6e−16
m = 0.77

Figure S6

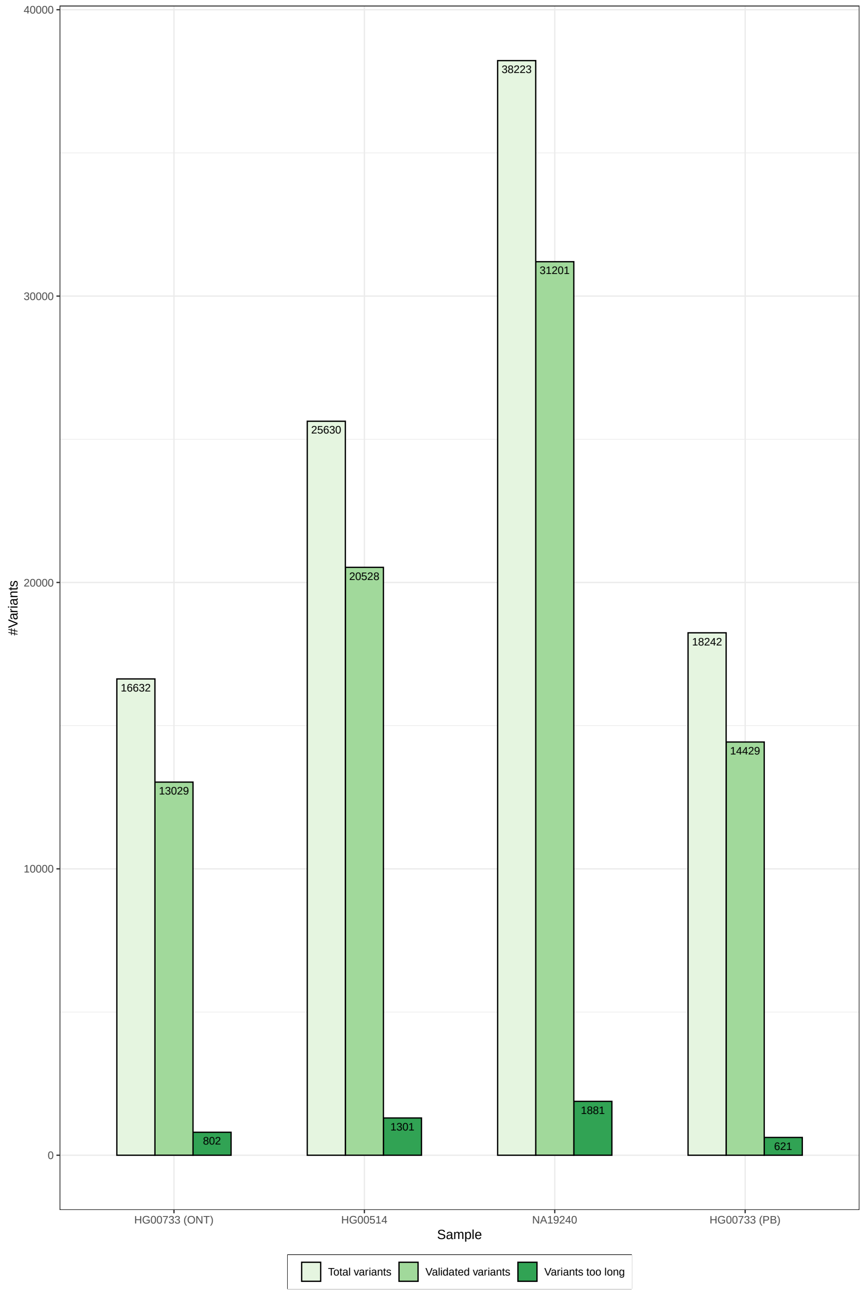
40000
30000
20000
10000
0
#Variants
38223
31201
1881
25630
20528
1301
16632
13029
802
18242
14429
621
HG00733 (ONT)
HG00514
NA19240
HG00733 (PB)
Sample
Total variants
Validated variants
Variants too long

Figure S7

References

[1] Ebler J, Haukness M, Pesout T, Marschall T, Paten B. Haplotype-aware diplotyping from noisy long reads. Genome Biol. 2019 Jun 3;20(1):116.

[2] Nielsen R, Paul JS, Albrechtsen A, Song YS. Genotype and SNP calling from next-generation sequencing data. Nat Rev Genet. 2011 Jun;12(6):443-51.

[3] Bryc K, Patterson N, Reich D. A novel approach to estimating heterozygosity from low-coverage genome sequence. Genetics. 2013 Oct;195(2):553-61.

[4] Bolognini D, Sanders A, Korbel JO, Magi A, Benes V, Rausch T. VISOR: a versatile haplotype-aware structural variant simulator for short and long read sequencing. Bioinformatics. 2019 Oct 7. pii: btz719.

[5] Patterson M, Marschall T, Pisanti N, van Iersel L, Stougie L, Klau GW, Schönhuth A. WhatsHap: Weighted Haplotype Assembly for Future-Generation Sequencing Reads. J Comput Biol. 2015 Jun;22(6):498-509.

[6] Edge P, Bansal V. Longshot enables accurate variant calling in diploid genomes from single-molecule long read sequencing. Nat Commun. 2019 Oct 11;10(1):4660.

[7] Choi Y, Chan AP, Kirkness E, Telenti A, Schork NJ. Comparison of phasing strategies for whole human genomes. PLoS Genet. 2018;14(4):e1007308.

[8] Danecek P, Auton A, Abecasis G, Albers CA, Banks E, DePristo MA, Handsaker RE, Lunter G, Marth GT, Sherry ST, McVean G, Durbin R; 1000 Genomes Project Analysis Group. The variant call format and VCFtools. Bioinformatics. 2011 Aug 1;27(15):2156-8.

[9] Edge P, Bafna V, Bansal V. HapCUT2: robust and accurate haplotype assembly for diverse sequencing technologies. Genome Res. 2017 May;27(5):801-812.

[10] Li H. A statistical framework for SNP calling, mutation discovery, association mapping and population genetical parameter estimation from sequencing data. Bioinformatics. 2011 Nov 1; 27(21): 2987–2993.

[11] Rausch T, Hsi-Yang Fritz M, Korbel JO, Benes V. Alfred: interactive multi-sample BAM alignment statistics, feature counting and feature annotation for long- and short-read sequencing. Bioinformatics. 2019 Jul 15;35(14):2489-2491.

[12] Chaisson MJP, Sanders AD, Zhao X, Malhotra A, Porubsky D, Rausch T, Gardner EJ et al. Multi-platform discovery of haplotype-resolved structural variation in human genomes. Nat Commun. 2019 Apr 16;10(1):1784.

[13] Gymrek M, Golan D, Rosset S, Erlich Y. lobSTR: A short tandem repeat profiler for personal genomes. Genome Res. 2012 Jun;22(6):1154-62.

[14] Vaser R, Sović I, Nagarajan N, Šikić M. Fast and accurate de novo genome assembly from long uncorrected reads. Genome Res. 2017 May;27(5):737-746.

[15] Lee C, Grasso C, Sharlow MF. Multiple sequence alignment using partial order graphs. Bioinformatics. 2002 Mar;18(3):452-64.

[16] Lee C. Generating consensus sequences from partial order multiple sequence alignment graphs. Bioinformatics. 2003 May 22;19(8):999-1008.

[17] Li H. Minimap2: pairwise alignment for nucleotide sequences. Bioinformatics. 2018 Sep 15;34(18):3094-3100.

[18] Sedlazeck FJ, Rescheneder P, Smolka M, Fang H, Nattestad M, von Haeseler A, Schatz AC. Accurate detection of complex structural variations using single molecule sequencing. Nat Methods. 2018 Jun; 15(6): 461–468.

[19] Ono Y, Asai K, Hamada M. PBSIM: PacBio reads simulator–toward accurate genome assembly. Bioinformatics. 2013 Jan 1;29(1):119-21.

[20] Harris RS, Cechova M, Makova KD, Birol I. Noise-cancelling repeat finder: Uncovering tandem repeats in error-prone long-read sequencing data. Bioinformatics. 2019 Nov 1;35(22):4809-4811.

[21] Michael TP, Jupe F, Bemm F, Motley ST, Sandoval JP, Lanz C, Loudet O, Weigel D, Ecker, JR. High contiguity Arabidopsis thaliana genome assembly with a single nanopore flow cell. Nat Commun. 2018; 9: 541.

[22] Pedersen, BS, Quinlan, AR. Mosdepth: Quick coverage calculation for genomes and exomes. Bioinformatics. 2018 Mar 1;34(5):867-868.
