# Supplementary material for: TRiCoLOR: tandem repeat profiling using whole-genome long-read sequencing data
Source: Gigascience. 2020 Oct 7;9(10):giaa101. doi: 10.1093/gigascience/giaa101 (PMC7539535; doi:10.1093/gigascience/giaa101)
Supplement: giaa101_Response_to_Reviewer_Comments_Original_Submission [file giaa101_response_to_reviewer_comments_original_submission.pdf]

Author's Response To Reviewer Comments

Close

Bolognini et al., point-by-point response to reviewers

We would like to thank all the Reviewers and the Editor for their constructive input and careful assessment of our manuscript. Based on their comments, our revised TRiCoLOR method and manuscript now includes:

- A tandem repeat callset generated by TRiCoLOR for the Arabidopsis thaliana KBS-Mac-74 model organism
- Additional benchmarks of tools for haplotype phasing to create haplotype-tagged input files for TRiCoLOR
- Several clarifications in the main text and in the supplementary information

TRiCoLOR has also been added to SciCrunch.org (RID: SCR_018801) and to bio.tools (bio.tools ID: tricolor).

Reviewer #1

Remarks to the authors: The authors present TRiCoLOR, a tool for genotyping tandem repeats and identifying tandem repeat expansions. While long-read sequencing holds the promise to elucidate this challenging type of variant, the software ecosystem is sparse and has not fully matured. As such TRiCoLOR is a valuable development, especially given the feature of de novo detection of tandem repeats. Both the manuscript and the software documentation are well written and clear. Please find my minor comments below.

Response: We thank the reviewer for the positive assessment of our manuscript.

Comment 1: Could the authors add recommendations to either the documentation or the manuscript on how to create the phased bam input files? I see Alfred is used in the manuscript, but given that multiple tools exist (also HapCut, WhatsHap, LongShot) it would be interesting to know if a comparison has been performed to find the best tool prior to TRiCoLOR. In addition, it could be useful for users to have recommendations on how to obtain a phased SNV vcf for their data. I expect this preparation step to be rather critical to the performance of TRiCoLOR.

Response: We agree with the Reviewer that a benchmark of the available tools for long-read phasing could be interesting, as this will help readers identifying a proper strategy for preparing their data for TRiCoLOR. We have now included in our supplementary informations (Supplementary Note S1) a detailed benchmark of widely-used methods for haplotype phasing (WhatsHap, LongShot and HapCUT2). Alfred is solely used for haplotype-splitting based on phased SNVs.

Comment 2: I see the authors missed or omit tandem-genotypes (https://genomebiology.biomedcentral.com/articles/10.1186/s13059-019-1667-6), an alternative tool for sizing tandem repeats, however without haplotype-specific genotyping. Including this tool in the comparison could be informative, however, it requires the slow and cumbersome LAST alignment prior to genotyping and I am glad to see your tool is compatible with more commonly used aligners.

Response: We apologise for missing tandem-genotypes in the initial draft. We have now included tandem-genotypes in the comparison (Background section). As the Reviewer anticipated, we noticed that the required preprocessing steps (last-train and lastal) are unwieldy when it comes to generate whole-genome alignments (30 to 40 times slower than minimap2 for aligning the HGSVC ONT samples) which prevent its application at large scale.

Comment 3: The installation of the tool was without issues, but I noticed that running the executable

without arguments raises an AttributeError where I would expect it to display help information. It would additionally be useful if the submodules' names could be made case-insensitive as this makes usage easier to remember.

Response: Thanks for noticing this bug. Now TRiCoLOR prints a help message when called without arguments. Following the Reviewer's suggestion, now TRiCoLOR's submodules (SENSoR, REFER, SAGE, ApP) are case-insensitive (for instance, the SENSoR module can be run as TRiCoLOR SENSoR or TRiCoLOR sensor interchangeably).

Reviewer #2

Remarks to the authors: Overall my impression is very positive. I believe the package addresses a practical use-case, and the evaluation uses reasonable experiments. However, in its current form there are several shortcomings in clarity that must be addressed. I think these can all be addressed without performing additional experiments. The following list is roughly in order of decreasing importance.

Response: We thank the reviewer for the generally positive assessment of our work. We have made several revisions based on the comments raised, as detailed below.

Comment 1: (Abstract, "The method can identify repetitive regions in sequencing data de novo ..."). I initially misinterpreted this, and it wasn't until the beginning of Methods that I figured it out. First, the use of the term de novo is easy to misunderstand, giving the reader the expectation that only sequenced reads are required. However, an assembled reference genome is required and the sequencing data has to be processed (e.g. by a haplotype-resolving aligner, not part of the package). I believe (as is mentioned at the end of Background, paragraph 4) the authors mean that the repeats themselves are discovered without any external definition of repeat motifs or locations. These distinctions should be made clearer in the abstract, and near the top of the Discussion section.

Response: We agree that the term "de novo" might create misunderstandings. We therefore now rephrased the abstract accordingly and the term is now introduced (and clarified) in the Background section (paragraph 5 and paragraph 13).

Comment 2: (Discussion "TRiCoLOR was primarily designed to profile ... microsatellites). This should be indicated in the abstract, as it will help clarify the intended use case.

Response: We have briefly reworded the Discussion section to clarify that TRiCoLOR profiles micro-satellites by default. We would like to clarify, however, that the regular expression algorithm can be tuned to profile mini-satellites as well (for instance, by setting the --size parameter to 10)

Comment 3: (Discussion, paragraph 2, "TRiCoLOR is technology agnostic and works with PB and ONT data"). Looking at the source code as well as the manuscript, the "technology agnostic" claim is a stretch. There are command line parameters that tune performance for either PB or ONT (e.g REFER readstype as mentioned in Note S7), and the evalution describes many settings to specify a technology. No evidence is presented to address anything other than PB or ONT technologies, hypothetical or real. The agnosticism claim should be justified or removed.

Response: We apologise for this inaccuracy and removed the agnosticism claim. We simply wanted to point out that TRiCoLOR works with both Oxford Nanopore Techonologies and Pacific Biosciences data.

Comment 3: (Benchmarking TRiCoLOR on real data, "...We calculated the number of TRs properly called by TRiCoLOR using a reference-free validation approach") A few sentences later it is revealed that a searchable FM-index of the human reference is used. I think the authors mean that it doesn't require a TR-annotated reference. But as it stands, the term reference-free is not how most readers would interpret it. This should be reworded to more accurately convey the concept.

Response: We thank the Reviewer for pointing this out, as it could have potentially been a source of misunderstanding for the readers. By "reference-free" we meant that the validation approach we used did not require the alignment of sequenced reads to an assembled reference genome (as this is potentially a source of biases for short-read sequencing datasets). We therefore reworded "reference-free" to "alignment-free", as this should help readers getting a clearer idea of our validation strategy.

Comment 4: (Discussion, paragraph 2, "... it can only deal with sequencing data from diploid individuals ...") It's not clear to me why this limitation exists, but a consequence of this is that it cannot identify repeats on human chromosome Y. This should be mentioned.

Response: We added this limitation in the Discussion section. Extending TRiCoLOR to non-diploid organisms is future work.

Comment 5: (Discussion) Because an assembled reference genome is required, the tool cannot identify repeats in genomic regions that are not assembled, such as chromosomes or telemeres. This should be mentioned with the other shortcomings.

Response: We thank the Reviewer for pointing out this oversight, we added this limitation in the Discussion section.

Comment 6: (Benchmarking TRiCoLOR on synthetic data, "... contractions/expansions of 7 motifs on average... [classification performance was] calculated allowing no discrepancies, 1 discrepancy or 2 discrepancies between the number of TRs in the ground truth and the number of TRs predicted by TRiCoLOR). It is not clear to me what a discrepancy is in this context. If the ground truth contained a TR with 10 copies of CAT, and TRiCoLOR reported a 12-copy CAT TR, is that "2 discrepancies"? My confusion may be because I think the authors are using TR and motif interchangebly here. But I have no alternative interpretation that is consistent with measuring the difference in the length of a particular TR. This description needs to be clarified.

Response: In this context we use the term "discrepancy" to indicate a different number of repeated motifs between the ground truth and TRiCoLOR's prediction. We changed "number of TRs" to "number of repeated motifs" in the text, which should help clarifying the results of our experiments.

Comment 7: (Benchmarking TRiCoLOR on real data, description of the process starting with "checks if the variant sequence appears at any position in the reference FM index") First, I think "the variant sequence is unique" is intended to mean "the variant sequence is unique in the reference". But I don't believe that is necessariy true. My interterpretation of "unique" is that the sequence occurs at one and only one position. If x is the variant sequence and it extends to, say, AxG, and AxG is not in the reference, this doesn't imply that Cx or xT is not in the reference. Nor does it imply that x occurs at only one position in the reference (though I believe the occurence count is something FM can give you).

Response: We would like to clarify a potential misunderstanding: The FM-Index is used to search all illumina reads efficiently (alignment-free). The additional comparison to the reference is only carried out to ensure that the tandem-repeat tagging sequence is long enough to not have any spurious matches in the reference. If such a tandem repeat tagging sequence does not occur in the reference but is supported by the illumina data we counted such events as true positives. This validation approach is of course bounded by the short read length, a limitation we already stated in our original submission.

Comment 8: (continuing) Second, in "up to 2 discrepancies", what is a discrepancy? Is this an edit distance of 2? In the Benchmarking on synthetic data section a discrepancy seemed to be the number of copies of the motif (I could have been wrong about that). I don't see any way to fit that meaning of discrepancy into the current context.

Response: We apologise for the misunderstanding and we indeed meant to use edit distance. We clarified this in the text and in the caption of Figure 1.

Comment 9: (Benchmarking TRiCoLOR on synthetic data, "... we compared TRiCoLOR to ... NCRF" and Figures 2 and S6). I have the same confusion here as in the previous paragraph. Figure 2 seems to make sense if the horizontal axis is the number of repeated motifs in a TR, and the vertical axis is the number of repeated motifs in the corresponding TR reported by the tool. It's difficult to evaluate this experiment as it is written. For example, I can't figure out if each of the 100 PB BAM files that contain expanded TRs contains only one TR or several.

Response: In all the simulations each BAM file harbours only one tandem repeat modification. We have highlighted this in the text and in the caption of Figure 2.

Comment 10. (Profiling repetitive regions, "... screened by a RegEx-based approximate string matching

algorithm ...") I didn't find any detail about the regular expression-based approximate string matching algorithm. It's not clear whether this algorithm will find only perfectly repeated motifs or if it allows some error (a la Wu and Manber's 1992 agrep). If it only finds perfect repeats a phrase indicating that should be added to the manuscript. If it allows for errors, this should be described either in this paragraph or in a supplementary section.

Response: Thanks, we further clarified the RegEx-based approximate string matching algorithm in the Methods section.

Comment 11: (Benchmarking TRiCoLOR on synthetic data, error ratios 45:25:20 and 15:50:35). These ratios were derived from real sequencing data, as described in Note S3. A sentence or short phrase should be added, telling the reader these numbers are justified by real data and point her to S3.

Response: We have added the required informations in the text, thanks for the suggestion.

Comment 12: (Benchmarking TRiCoLOR on synthetic data, ~8000 bps). The 8K average read length is used in several simulations here, but I see no justification given. Figures S1 and S2 of Ono's 2013 paper suggest a much shorter mean length, and indeed the default for pbsim is 3K. Of course 2013 is ancient history and PB technology (probably) gives longer lengths now. I suspect the authors derived the 8K number by examining lengths in some real dataset; if so that should be mentioned in a supplementary note. (I suspect the authors may have derived it from the same data mentioned in note S3.) Moreover, one might expect ONT to given different lengths. I assume the use of 8K for both technologies is to eliminate a possible source of bias. But the authors should give some argument why the 8K value is reasonable to ONT.

Response: We derived the mean length of Oxford Nanopore Technology reads using real datasets from one of our previous works (Bolognini et al., PLoS One. 2019, Figure 2), which we have now added as a citation in the text. We indeed used the same length for ONT and PacBio to not introduce any length bias.

Comment 13: (Profiling repetitive regions, "With the haplotype-specific consensus sequences at hand ... Supplementary Note S4 ...)" Note S4 describes an evaluation of aligning raw reads to a reference, as opposed to aligning consensus sequences to reference. The conclusion that minimap2 is the winner may be true even for consensus sequences. But I'm concerned about what paramterization of minimap2 was used to map consensus to reference. I expect it should be either asm5 or asm10, and in particular the presets used for PB or ONT reads shouldn't be expected to give the best alignments for the consensuses. Consider adding a paragraph to S4, or a separate short supplemental note describing the minimap2 parameters used to align consensus to reference.

Response: We have now included in Supplementary Note S4 a benchmark of several of minimap2's presets of parameters (map-ont/map-pb, asm5, asm10 and asm20) that we used to align SPOA-generated consensus sequences to the reference genome. Although the assembly-to-reference parameters (asm5, asm10, asm20) are ideally well-suited to align consensus sequences to the reference genome, we did not notice any differences in terms of mapping accuracy between these parameters and those tuned for aligning noisy reads (map-ont/map-pb). For the time being, we are calling minimap2 from within TRiCoLOR using the map-ont/map-pb presets.

Comment 14: (Benchmarking TRiCoLOR on real data, "... the module identified ~160000, ~190000 and ~260000 low-entropy regions ...) Are these regions the same size as the SENSOR windows (i.e. 20 bp)? Or have neighboring windows been joined into longer intervals? If it is the former it would be clearer to use "window" instead of "region". If it's the latter, a statement about the average region length, or sum of regions lengths, would be appropriate. It really depends on what the author's want to convey by these numbers. If they are evidence that filtering for entropy and depth reduces the workload, the fraction of the genome excluded by these steps would be worth knowing. If instead this is intended as a result of biological significance, the fraction of the genome covered by these regions would be interesting.

Response: Filtering on coverage depth improves the false discovery rate of TRiCoLOR and it also reduces the workload of the subsequent tandem repeat profiling step. As suggested by the reviewer, we included in the text the average length of the low-entropy regions identified by TRiCoLOR SENSoR (which resulted from merging nearby low-entropy windows).

Comment 15: (Benchmarking TRiCoLOR on real data, "...often impossible to accurately map or even assemble short reads originating from repetitive regions, thus generating errors in the available TR callsets"). "false negatives" would be clearer than "errors". Or something like "thus some TRs are missing from the available TR callsets."

Response: We thank the Reviewer of his suggestion and we changed the text accordingly.

Comment 16: (Background, paragraph 3, "... accurately deciphering TRs from long reads remains a considerable challenge due to their high error rates ...") This is ambiguous -- it's not clear (as written) whether it's long reads that have high error rates, or TRs, unless the reader has previous knowledge. Please reword it to make it clearer

Response: We reworded the text.

Comment 17: (Benchmarking TRiCoLOR on real data, "Using an Ubuntu 16.04.6 LTS desktop with Intel Xeon processors X5460, the module took ~4 hours ...") I'm used to seeing clock rates as part of the CPU specs in benchmarks like this. Is the CPU speed inherent to the processor model number?

Response: We have added the clock rate as part of our setting specification, both in the main text and in the supplementary informations.

Comment 18: (Benchmarking TRiCoLOR on real data, "we could visually confirm 42 of them in the assembly). It wasn't immediately clear to me what "assembly" means. Is the the human reference, or is it a separate assembly from HGSVC, prehaps an assembly of HG00733?

Response: We apologise for the misunderstanding. We were referring to the HGSVC de novo assembly for the HG00733 individual. We have now clarified this in the text.

Comment 19: (Note S4) PBSIM was designed to simulate PacBio error profiles. How confident should we be that just by changing the --accuracy-mean and --difference-ratio parameters that it will do a decent job of modelling ONT reads? For example, what model_qc setting is appropriate for ONT? But answering that would be beyond the scope of this paper.

Response: We independently evaluated simulated and real Oxford Nanopore Technology data sets and these settings yielded comparable insertion, deletion and mismatch error rates. Currently unknown ONT biases we may fail to model adequately but this is indeed beyond the scope of this manuscript, we believe.

Comment 20: (Note S3, "For ONT alignments, the mean error rate is ~11% and ...") It should be made clear that these are the error rates and ratios before correction. It it would also be interesting to know what the ratios are after correcton, if the authors have that information handy.

Response: We have added this information to Supplementary Note S3.

Reviewer #3

Remarks to the authors: The paper described a new method TRiCoLOR for discovering tandem repeat in the human genome by using long reads generated by PacBio or ONT. The authors demonstrated that it outperformed other alternative programs. The concern this reviewer has is that its application is rather limited. The application of this method could be valuable, if the authors can do the following:

Comment 1. Make it work for organisms other than human;

Response: TRiCoLOR is well suited to profile tandem repeats in any diploid organism. We agree with the Reviewer that a tandem repeat callset for an organism other than human is a valuable addition to the manuscript to convince readers of the wide applicability of our method. We therefore used TRiCoLOR for one Arabidopsis thaliana sample recently sequenced with ONT (https://www.nature.com/articles/s41467-018-03016-2). Further details of this tandem repeat callset generated by TRiCoLOR have been added in the supplementary information (Supplementary Note S8).

Comment 2. Compare its performance against assembly-alignment-based approach. Users can assemble PacBio or ONT reads into contigs, and identify tandem repeats via aligning contigs against reference genome sequences;

Response: We would like to clarify that such a comparison was part of our original manuscript, please see Table 1 in the main manuscript.

Comment 3. PacBio HIFI reads are of much improved accuracy and lengths, which may be of great value in identifying tandem repeats directly, limiting the value of TRiCoLOR.

Reponse: PacBio HIFI reads are expected to improve de novo assemblies at an albeit much higher cost per base. TRiCoLOR's RegEx-based algorithm likewise benefits directly from highly accurate reads. In addition, mapping-based approaches such as TRiCoLOR are expected to work at lower coverages in case of highly accurate reads, possibly providing cost-advantages compared to haplotype-specific assemblies that may require deeper coverage and additional data for long-range phasing information (HiC or Strand-Seq).

Close
