# Supplementary material for: TRiCoLOR: tandem repeat profiling using whole-genome long-read sequencing data
Source: Gigascience. 2020 Oct 7;9(10):giaa101. doi: 10.1093/gigascience/giaa101 (PMC7539535; doi:10.1093/gigascience/giaa101)
Supplement: giaa101_Response_to_Reviewer_Comments_Revision_1 [file giaa101_response_to_reviewer_comments_revision_1.pdf]

# Author's Response To Reviewer Comments

Close

Bolognini et al., point-by-point response to reviewers.

Reviewer #1

Comment 1: I thank the authors for addressing my comments, making subcommands case insensitive and adding the help message when executed without subcommand.

Response: Thanks.

Comment 2: The evaluation of the available tools for long-read phasing is informative, however, if I interpret the findings correctly the performance is rather sobering, with WhatsHap and LongShot only assigning < ⅔ of the reads, and only correctly assigning to the right haplotype for just above half of those assigned? But also Alfred, for splitting reads using variants from short read sequencing phased by HapCut2, performs poorly. It is only when an already phased VCF from short read sequencing is used that high phasing accuracy is obtained. As such I have serious reservations about the validity of any approach requiring phased reads in the absence of high quality variants identified from short read sequencing. Very few projects will have the ideal setup with short reads for high quality variant calling, Strand-Seq data to phase variants and long reads. Have the authors evaluated the impact of poor phasing performance on their tool? Could the authors consider a way to use TRiCoLOR without having to phase reads, potentially by per repeat locus clustering of reads? While TRiCoLOR itself is not intended to solve the phasing problem, the absence of good solutions are a serious limitation and may in the current state limit the usability of your tool, probably warranting more than a supplementary note to this manuscript.

Response: Thanks for pointing out this issue that could easily create misunderstandings. First of all, we would like to clarify that read-backed phasing works fine for TRiCoLOR, the only difference is that the phasing is fragmented in blocks and doesn't extend across entire chromosomes. Within each phased block (N50 block lengths of ~80 kbps for Longshot and ~55 kbps for WhatsHap when we used them to directly identify and phase single-nucleotide variants from our simulated long-read alignments) the haplotype assignments are consistent and it is primarily in-between haplotype blocks where switch errors may occur (i.e., an incorrect assignment of phase between two adjacent heterozygous variants). Unfortunately, our previous evaluation compared haplotype assignments to the original simulated haplotypes across the entire chromosome but for read-backed phasing, haplotype assignments might switch from one phased block to the next (as explained above) and we did not take care of that issue in our previous analysis. We have now corrected this error and evaluated the haplotype assignment for each phased block, yielding ~70% haplotype-assigned reads and ~90% haplotype-assigned bases because most unassigned reads are short (as expected). Locally these haplotype assignments are thus correct and TRiCoLOR correctly clusters reads per haplotype in each repeat locus (as suggested by the reviewer). The additional benefit of Hi-C or Strand-Seq sequencing data is the ability to correct remaining switch errors (estimated to be ~1% in our experiment) to provide chromosome-level phasing information which can be informative for studying recombination or long-range cis-effects but for TRiCoLOR phased blocks are sufficient. We updated the Supplement accordingly and now evaluate the haplotype assignment for each phased block, apologies for the oversight in the first revision.

Comment 3: While probably out of scope and not necessarily relevant for a Technical Note, I wonder how recently developed tools such as ExpansionHunter Denovo on PCR-free short read WGS correlate to the findings with TRiCoLOR on long reads.

Response: We indeed felt that the manuscript already contains a wealth of benchmarking information for long read alignments, phasing, haplotype assignments and tandem repeat calling (assembly-based and mapping-based). Given the lack of a gold standard of tandem repeats we did not want to overload the manuscript with yet another comparison to short-read tandem repeat discovery methods, especially

since these are greatly limited by the read length. We do consider the integration of short- and long-read tandem repeat calls out of the scope of this manuscript.

Close
