# Supplementary material for: TRiCoLOR: tandem repeat profiling using whole-genome long-read sequencing data
Source: Gigascience. 2020 Oct 7;9(10):giaa101. doi: 10.1093/gigascience/giaa101 (PMC7539535; doi:10.1093/gigascience/giaa101)
Supplement: giaa101_Response_to_Reviewer_Comments_Revision_2 [file giaa101_response_to_reviewer_comments_revision_2.pdf]

Author's Response To Reviewer Comments

Close

Dear Editor,

thanks for Your editorial work with our manuscript. We have addressed the minor edits required to meet GigaScience’s formatting and reproducibility guidelines, as from Your decision letter. Thank You for publishing our revised manuscript in GigaScience.

Close
