# Supplementary material for: TRiCoLOR: tandem repeat profiling using whole-genome long-read sequencing data
Source: Gigascience. 2020 Oct 7;9(10):giaa101. doi: 10.1093/gigascience/giaa101 (PMC7539535; doi:10.1093/gigascience/giaa101)
Supplement: giaa101_Reviewer_1_Report_Original_Submission — Wouter De Coster -- 6/23/2020 Reviewed [file giaa101_reviewer_1_report_original_submission.pdf]

Reviewer Report

Title: TRiCoLOR: tandem repeat profiling using whole-genome long-read sequencing data

Version: Original Submission **Date:** 6/23/2020

Reviewer name: Wouter De Coster

Reviewer Comments to Author:

The authors present TRiCoLOR, a tool for genotyping tandem repeats and identifying tandem repeat expansions. While long-read sequencing holds the promise to elucidate this challenging type of variant, the software ecosystem is sparse and has not fully matured. As such TRiCoLOR is a valuable development, especially given the feature of de novo detection of tandem repeats. Both the manuscript and the software documentation are well written and clear.
Please find my minor comments below.
Sincerely,
Wouter De Coster
Could the authors add recommendations to either the documentation or the manuscript on how to create the phased bam input files? I see Alfred is used in the manuscript, but given that multiple tools exist (also HapCut, WhatsHap, LongShot) it would be interesting to know if a comparison has been performed to find the best tool prior to TRiCoLOR. In addition, it could be useful for users to have recommendations on how to obtain a phased SNV vcf for their data. I expect this preparation step to be rather critical to the performance of TRiCoLOR?
I see the authors missed or omit tandem-genotypes (https://genomebiology.biomedcentral.com/articles/10.1186/s13059-019-1667-6), an alternative tool for sizing tandem repeats, however without haplotype-specific genotyping. Including this tool in the comparison could be informative, however, it requires the slow and cumbersome LAST alignment prior to genotyping and I am glad to see your tool is compatible with more commonly used aligners.
The installation of the tool was without issues, but I noticed that running the executable without arguments raises an AttributeError where I would expect it to display help information. It would additionally be useful if the submodules' names could be made case-insensitive as this makes usage easier to remember.

Level of Interest

Please indicate how interesting you found the manuscript: Choose an item.

Quality of Written English

Please indicate the quality of language in the manuscript: Choose an item.

Declaration of Competing Interests

Please complete a declaration of competing interests, considering the following questions:

- Have you in the past five years received reimbursements, fees, funding, or salary from an organisation that may in any way gain or lose financially from the publication of this manuscript, either now or in the future?
- Do you hold any stocks or shares in an organisation that may in any way gain or lose financially from the publication of this manuscript, either now or in the future?
- Do you hold or are you currently applying for any patents relating to the content of the manuscript?
- Have you received reimbursements, fees, funding, or salary from an organization that holds or has applied for patents relating to the content of the manuscript?
- Do you have any other financial competing interests?
- Do you have any non-financial competing interests in relation to this paper?

If you can answer no to all of the above, write 'I declare that I have no competing interests' below. If your reply is yes to any, please give details below.

I have received travel reimbursements and free consumables from Oxford Nanopore Technologies, but do not consider this to affect my judgment regarding this manuscript.

I agree to the open peer review policy of the journal. I understand that my name will be included on my report to the authors and, if the manuscript is accepted for publication, my named report including any attachments I upload will be posted on the website along with the authors' responses. I agree for my report to be made available under an Open Access Creative Commons CC-BY license (http://creativecommons.org/licenses/by/4.0/). I understand that any comments which I do not wish to be included in my named report can be included as confidential comments to the editors, which will not be published.

Choose an item.

To further support our reviewers, we have joined with Publons, where you can gain additional credit to further highlight your hard work (see: https://publons.com/journal/530/gigascience). On publication of this paper, your review will be automatically added to Publons, you can then choose whether or not to claim your Publons credit. I understand this statement.

Yes Choose an item.
