# Supplementary material for: TRiCoLOR: tandem repeat profiling using whole-genome long-read sequencing data
Source: Gigascience. 2020 Oct 7;9(10):giaa101. doi: 10.1093/gigascience/giaa101 (PMC7539535; doi:10.1093/gigascience/giaa101)
Supplement: giaa101_Reviewer_1_Report_Revision_1 — Wouter De Coster -- 8/14/2020 Reviewed [file giaa101_reviewer_1_report_revision_1.pdf]

Reviewer Report

Title: TRiCoLOR: tandem repeat profiling using whole-genome long-read sequencing data

Version: Revision 1 **Date:** 8/14/2020

Reviewer name: Wouter De Coster

Reviewer Comments to Author:

I thank the authors for addressing my comments, making subcommands case insensitive and adding the help message when executed without subcommand. The evaluation of the available tools for long-read phasing is informative, however, if I interpret the findings correctly the performance is rather sobering, with WhatsHap and LongShot only assigning < â…” of the reads, and only correctly assigning to the right haplotype for just above half of those assigned? But also Alfred, for splitting reads using variants from short read sequencing phased by HapCut2, performs poorly. It is only when an already phased VCF from short read sequencing is used that high phasing accuracy is obtained. As such I have serious reservations about the validity of any approach requiring phased reads in the absence of high quality variants identified from short read sequencing. Very few projects will have the ideal setup with short reads for high quality variant calling, Strand-Seq data to phase variants and long reads. Have the authors evaluated the impact of poor phasing performance on their tool? Could the authors consider a way to use TRiCoLOR without having to phase reads, potentially by per repeat locus clustering of reads? While TRiCoLOR itself is not intended to solve the phasing problem, the absence of good solutions are a serious limitation and may in the current state limit the usability of your tool, probably warranting more than a supplementary note to this manuscript.
While probably out of scope and not necessarily relevant for a Technical Note, I wonder how recently developed tools such as ExpansionHunter Denovo on PCR-free short read WGS correlate to the findings with TRiCoLOR on long reads.

Level of Interest

Please indicate how interesting you found the manuscript: Choose an item.

Quality of Written English

Please indicate the quality of language in the manuscript: Choose an item.

Declaration of Competing Interests

Please complete a declaration of competing interests, considering the following questions:

- Have you in the past five years received reimbursements, fees, funding, or salary from an organisation that may in any way gain or lose financially from the publication of this manuscript, either now or in the future?

- Do you hold any stocks or shares in an organisation that may in any way gain or lose financially from the publication of this manuscript, either now or in the future?
- Do you hold or are you currently applying for any patents relating to the content of the manuscript?
- Have you received reimbursements, fees, funding, or salary from an organization that holds or has applied for patents relating to the content of the manuscript?
- Do you have any other financial competing interests?
- Do you have any non-financial competing interests in relation to this paper?

If you can answer no to all of the above, write 'I declare that I have no competing interests' below. If your reply is yes to any, please give details below.

I have received travel reimbursements and free consumables from Oxford Nanopore Technologies, but do not consider this to affect my judgment regarding this manuscript.

I agree to the open peer review policy of the journal. I understand that my name will be included on my report to the authors and, if the manuscript is accepted for publication, my named report including any attachments I upload will be posted on the website along with the authors' responses. I agree for my report to be made available under an Open Access Creative Commons CC-BY license (http://creativecommons.org/licenses/by/4.0/). I understand that any comments which I do not wish to be included in my named report can be included as confidential comments to the editors, which will not be published.

Choose an item.

To further support our reviewers, we have joined with Publons, where you can gain additional credit to further highlight your hard work (see: https://publons.com/journal/530/gigascience). On publication of this paper, your review will be automatically added to Publons, you can then choose whether or not to claim your Publons credit. I understand this statement.

Yes Choose an item.
