# Supplementary material for: TRiCoLOR: tandem repeat profiling using whole-genome long-read sequencing data
Source: Gigascience. 2020 Oct 7;9(10):giaa101. doi: 10.1093/gigascience/giaa101 (PMC7539535; doi:10.1093/gigascience/giaa101)
Supplement: giaa101_Reviewer_2_Report_Original_Submission — Robert S Harris -- 6/28/2020 Reviewed [file giaa101_reviewer_2_report_original_submission.pdf]

Reviewer Report

Title: TRiCoLOR: tandem repeat profiling using whole-genome long-read sequencing data

Version: Original Submission **Date:** 6/28/2020

Reviewer name: Robert S Harris

Reviewer Comments to Author:

Overall my impression is very positive. I believe the package addresses a practical use-case, and the evaluation uses reasonable experiments. However, in its current form there are several shortcomings in clarity that must be addressed. I think these can all be addressed without performing additional experiements.
The following list is roughly in order of decreasing importance.
(Abstract, "The method can identify repetitive regions in sequencing data de novo ..."). I initially misinterpreted this, and it wasn't until the beginning of Methods that I figured it out. First, the use of the term de novo is easy to misunderstand, giving the reader the expectation that only sequenced reads are required. However, an assembled reference genome is required and the sequencing data has to be processed (e.g. by a haplotype-resolving aligner, not part of the package). I believe (as is mentioned at the end of Background, paragraph 4) the authors mean that the repeats themselves are discovered without any external definition of repeat motifs or locations. These distinctions should be made clearer in the abstract, and near the top of the Discussion section.
(Discussion "TRiCoLOR was primarily designed to profile ... microsatellites). This should be indicated in the abstract, as it will help clarify the intended use case.
(Discussion, paragraph 2, "TRiCoLOR is technology agnostic and works with PB and ONT data"). Looking at the source code as well as the manuscript, the "technology agnostic" claim is a stretch. There are command line parameters that tune performance for either PB or ONT (e.g REFER readstype as mentioned in Note S7), and the evalution describes many settings to specify a technology. No evidence is presented to address anything other than PB or ONT technologies, hypothetical or real. The agnosticism claim should be justified or removed.
(Benchmarking TRiCoLOR on real data, "...We calculated the number of TRs properly called by TRiCoLOR using a reference-free validation approach") A few sentences later it is revealed that a searchable FM-index of the human reference is used. I think the authors mean that it doesn't require a TR-annotated reference. But as it stands, the term reference-free is not how most readers would interpret it. This should be reworded to more accurately convey the concept.
(Discussion, paragraph 2, "... it can only deal with sequencing data from diploid individuals ...") It's not clear to me why this limitation exists, but a consequence of this is that it cannot identify repeats on human chromosome Y. This should be mentioned.
(Discussion) Because an assembled reference genome is required, the tool cannot identify repeats in genomic regions that are not assembled, such as chromosomes or telemeres. This should be mentioned with the other shortcomings.
(Benchmarking TRiCoLOR on synthetic data, "... contractions/expansions of 7 motifs on average...

[classification performance was] calculated allowing no discrepancies, 1 discrepancy or 2 discrepancies between the number of TRs in the ground truth and the number of TRs predicted by TRiCoLOR). It is not clear to me what a discrepancy is in this context. If the ground truth contained a TR with 10 copies of CAT, and TRiCoLOR reported a 12-copy CAT TR, is that "2 discrepancies"? My confusion may be because I think the authors are using TR and motif interchangebly here. But I have no alternative interpretation that is consistent with measuring the difference in the length of a particular TR. This description needs to be clarified.

(Benchmarking TRiCoLOR on real data, description of the process starting with "checks if the variant sequence appears at any position in the reference FM index") First, I think "the variant sequence is unique" is intended to mean "the variant sequence is unique in the reference". But I don't believe that is necessariy true. My interterpretation of "unique" is that the sequence occurs at one and only one position. If x is the variant sequence and it extends to, say, AxG, and AxG is not in the reference, this doesn't imply that Cx or xT is not in the reference. Nor does it imply that x occurs at only one position in the reference (though I believe the occurence count is something FM can give you).

(continuing) Second, in "up to 2 discrepancies", what is a discrepancy? Is this an edit distance of 2? In the Benchmarking on synthetic data section a discrepancy seemed to be the number of copies of the motif (I could have been wrong about that). I don't see any way to fit that meaning of discrepancy into the current context.

(Benchmarking TRiCoLOR on synthetic data, "... we compared TRiCoLOR to ... NCRF" and Figures 2 and S6). I have the same confusion here as in the previous paragraph. Figure 2 seems to make sense if the horizontal axis is the number of repeated motifs in a TR, and the vertical axis is the number of repeated motifs in the corresponding TR reported by the tool. It's difficult to evaluate this experiment as it is written. For example, I can't figure out if each of the 100 PB BAM files that contain expanded TRs contains only one TR or several.

(Profiling repetitive regions, "... screened by a RegEx-based approximate string matching algorithm ...") I didn't find any detail about the regular expression-based approximate string matching algorithm. It's not clear whether this algorithm will find only perfectly repeated motifs or if it allows some error (a la Wu and Manber's 1992 agrep). If it only finds perfect repeats a phrase indicating that should be added to the manuscript. If it allows for errors, this should be described either in this paragraph or in a supplementary section.

(Benchmarking TRiCoLOR on synthetic data, error ratios 45:25:20 and 15:50:35). These ratios were derived from real sequencing data, as described in Note S3. A sentence or short phrase should be added, telling the reader these numbers are justified by real data and point her to S3.

(Benchmarking TRiCoLOR on synthetic data, ~8000 bps). The 8K average read length is used in several simulations here, but I see no justification given. Figures S1 and S2 of Ono's 2013 paper suggest a much shorter mean length, and indeed the default for pbsim is 3K. Of course 2013 is ancient history and PB technology (probably) gives longer lengths now. I suspect the authors derived the 8K number by examining lengths in some real dataset; if so that should be mentioned in a supplementary note. (I suspect the authors may have derived it from the same data mentioned in note S3.) Moreover, one might expect ONT to given different lengths. I assume the use of 8K for both technologies is to eliminate a possible source of bias. But the authors should give some argument why the 8K value is reasonable to ONT.

(Profiling repetitive regions, "With the haplotype-specific consensus sequences at hand ... Supplementary Note S4 ...)" Note S4 describes an evaluation of aligning raw reads to a reference, as opposed to aligning consensus sequences to reference. The conclusion that minimap2 is the winner may be true even for consensus sequences. But I'm concerned about what paramterization of minimap2 was used to map consensus to reference. I expect it should be either asm5 or asm10, and in particular the presets used for PB or ONT reads shouldn't be expected to give the best alignments for the consensuses. Consider adding a paragraph to S4, or a separate short supplemental note describing the minimap2 parameters used to align consensus to reference.
(Benchmarking TRiCoLOR on real data, "... the module identified âˆ¼160000, âˆ¼190000 and âˆ¼260000 low-entropy regions ...) Are these regions the same size as the SENSOR windows (i.e. 20 bp)? Or have neighboring windows been joined into longer intervals? If it is the former it would be clearer to use "window" instead of "region". If it's the latter, a statement about the average region length, or sum of regions lengths, would be appropriate. It really depends on what the author's want to convey by these numbers. If they are evidence that filtering for entropy and depth reduces the workload, the fraction of the genome excluded by these steps would be worth knowing. If instead this is intended as a result of biological significance, the fraction of the genome covered by these regions would be interesting.
(Benchmarking TRiCoLOR on real data, "...often impossible to accurately map or even assemble short reads originating from repetitive regions, thus generating errors in the available TR callsets"). "false negatives" would be clearer than "errors". Or something like "thus some TRs are missing from the available TR callsets."
(Background, paragraph 3, "... accurately deciphering TRs from long reads remains a considerable challenge due to their high error rates ...") This is ambiguous -- it's not clear (as written) whether it's long reads that have high error rates, or TRs, unless the reader has previous knowledge. Please reword it to make it clearer.
(Benchmarking TRiCoLOR on real data, "Using an Ubuntu 16.04.6 LTS desktop with Intel Xeon processors X5460, the module took âˆ¼4 hours ...") I'm used to seeing clock rates as part of the CPU specs in benchmarks like this. Is the CPU speed inherent to the processor model number?
(Benchmarking TRiCoLOR on real data, "we could visually confirm 42 of them in the assembly). It wasn't immediately clear to me what "assembly" means. Is the the human reference, or is it a separate assembly from HGSVC, prehaps an assembly of HG00733?
(Note S4) PBSIM was designed to simulate PacBio error profiles. How confident should we be that just by changing the --accuracy-mean and --difference-ratio parameters that it will do a decent job of modelling ONT reads? For example, what model_qc setting is appropriate for ONT? But answering that would be beyond the scope of this paper.
(Note S3, "For ONT alignments, the mean error rate is âˆ¼11% and ...") It should be made clear that these are the error rates and ratios before correction. It it would also be interesting to know what the ratios are after correcton, if the authors have that information handy.
(Note S2) (I'm not asking for any change or clarification.) But I'm surprised that simulated reads were used instead of real reads.

Level of Interest

Please indicate how interesting you found the manuscript: Choose an item.

Quality of Written English

Please indicate the quality of language in the manuscript: Choose an item.

Declaration of Competing Interests

Please complete a declaration of competing interests, considering the following questions:

- Have you in the past five years received reimbursements, fees, funding, or salary from an organisation that may in any way gain or lose financially from the publication of this manuscript, either now or in the future?
- Do you hold any stocks or shares in an organisation that may in any way gain or lose financially from the publication of this manuscript, either now or in the future?
- Do you hold or are you currently applying for any patents relating to the content of the manuscript?
- Have you received reimbursements, fees, funding, or salary from an organization that holds or has applied for patents relating to the content of the manuscript?
- Do you have any other financial competing interests?
- Do you have any non-financial competing interests in relation to this paper?

If you can answer no to all of the above, write 'I declare that I have no competing interests' below. If your reply is yes to any, please give details below.

I declare that I have no competing interests

I agree to the open peer review policy of the journal. I understand that my name will be included on my report to the authors and, if the manuscript is accepted for publication, my named report including any attachments I upload will be posted on the website along with the authors' responses. I agree for my report to be made available under an Open Access Creative Commons CC-BY license (http://creativecommons.org/licenses/by/4.0/). I understand that any comments which I do not wish to be included in my named report can be included as confidential comments to the editors, which will not be published.

Choose an item.

To further support our reviewers, we have joined with Publons, where you can gain additional credit to further highlight your hard work (see: https://publons.com/journal/530/gigascience). On publication of this paper, your review will be automatically added to Publons, you can then choose whether or not to claim your Publons credit. I understand this statement.

Yes Choose an item.
